# NeoPHOX – a structurally tunable ligand system for asymmetric catalysis

**DOI:** 10.3762/bjoc.12.114

**Published:** 2016-06-13

**Authors:** Jaroslav Padevět, Marcus G Schrems, Robin Scheil, Andreas Pfaltz

**Affiliations:** 1 Department of Chemistry, University of Basel, St. Johanns-Ring 19, CH-4056 Basel, Switzerland

**Keywords:** allylic substitution, asymmetric hydrogenation, iridium, N,P ligand, palladium

## Abstract

A synthesis of new NeoPHOX ligands derived from serine or threonine has been developed. The central intermediate is a NeoPHOX derivative bearing a methoxycarbonyl group at the stereogenic center next to the oxazoline N atom. The addition of methylmagnesium chloride leads to a tertiary alcohol, which can be acylated or silylated to produce NeoPHOX ligands with different sterical demand. The new NeoPHOX ligands were tested in the iridium-catalyzed asymmetric hydrogenation and palladium-catalyzed allylic substitution. In both reactions high enantioselectivities were achieved, that were comparable to the enantioselectivities obtained with the up to now best NeoPHOX ligand derived from expensive *tert*-leucine.

## Introduction

Since their introduction and first successful application in enantioselective palladium-catalyzed allylic substitution in 1993 [1–3], chiral phosphinooxazolines (PHOX ligands) have emerged as a widely used privileged ligand class [4–12]. One of the major areas of application of PHOX and related N,P ligands is the iridium-catalyzed asymmetric hydrogenation [13–16]. Compared to rhodium and ruthenium catalysts, iridium complexes derived from chiral N,P ligands show a much broader substrate scope in the hydrogenation of olefins, as they do not require any coordinating groups in the substrate.

Although high enantioselectivities were achieved with the initially developed Ir-PHOX catalysts, the range of olefins that gave satisfactory results was limited. Consequently, a variety of other ligand classes derived from oxazolines or other nitrogen heterocycles were explored and eventually, several highly effective ligand systems were found that have considerably enhanced the range of functionalized and unfunctionalized olefins that can be hydrogenated with high efficiency and excellent enantioselectivity. Among the many oxazoline-derived ligands that have been reported in the literature, SimplePHOX [17] and ThrePHOX [18] have emerged as some of the most versatile and most easily accessible ligand classes. However, although iridium complexes derived from these ligands are air and moisture-stable, the free ligands are prone to hydrolysis and oxidation. Especially SimplePHOX ligands, although they are readily prepared from amino alcohols in just two steps, suffer from these problems resulting in low yields and difficult purification steps.

**Figure 1:**
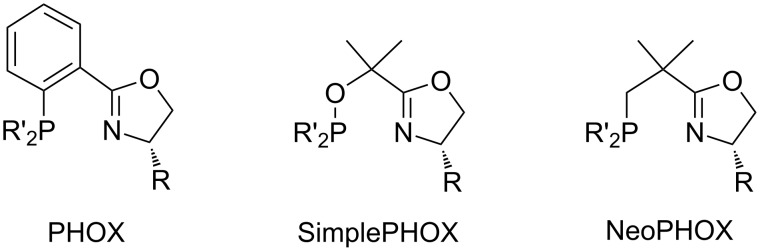
Structural motifs of phospinooxazoline ligands.

We therefore decided to explore structural analogs, in which the P–O bond had been replaced by a P–C bond [19]. We hoped that such ligands, which we named NeoPHOX because of the neopentyl backbone, would be as easily accessible as SimplePHOX but more stable against air and moisture and fulfill the criteria for a scalable high yielding synthesis (Figure 1).

## Results and Discussion

### 1st Generation NeoPHOX ligands

**Scheme 1:**
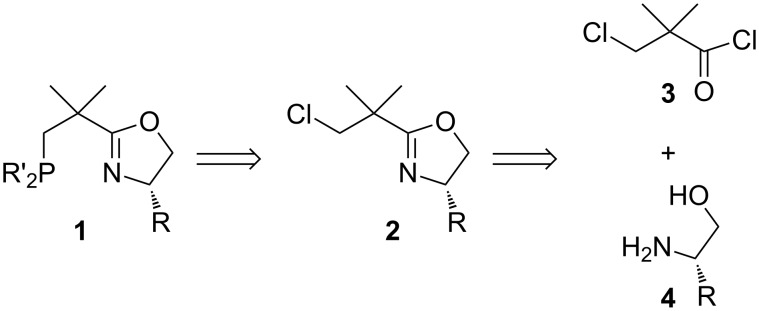
Retrosynthetic analysis for NeoPHOX ligands.

A potential very short route to NeoPHOX ligands starting from commercially available 3-chloropivaloyl chloride (**3**) is shown in Scheme 1. Although nucleophilic substitutions at the neopentyl carbon atoms are known to be difficult, literature precedence indicated that the introduction of the phosphine group might be possible by the reaction of a diarylphosphide anion with chloroalkyloxazoline **2**.

Ashby et al. [20] investigated the reactivity of neopentyl halide systems with various metal diphenylphosphides and found that the reaction indeed took place. While for iodides evidence for a radical single electron transfer process as the major pathway was found, the corresponding bromides and chlorides seemed to prefer an S_N_2 mechanism. Rossi et al. [21] showed that substitution products can be obtained in good yields from neopentyl chloride derivatives and sodium diphenylphosphide in liquid ammonia by an S_NR_2 reaction induced by UV irradiation. Huttner and co-workers [22] on the other hand achieved high yields in nucleophilic substitutions of diarylphosphines with MeC(CH_2_Cl)_3_ in DMSO using aqueous KOH as base at elevated temperature but without irradiation.

**Scheme 2:**
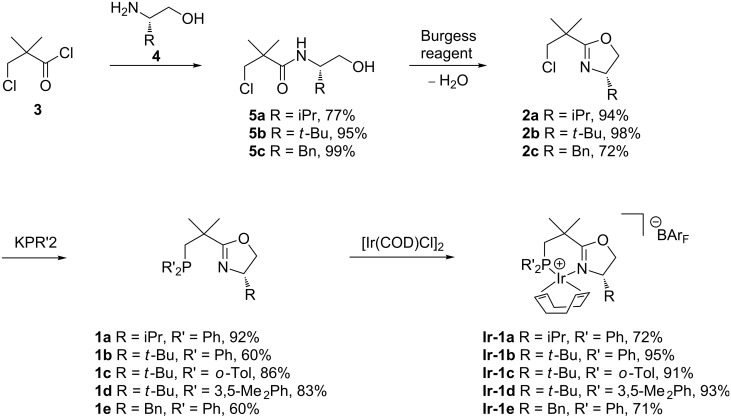
Synthesis of 1st generation NeoPHOX Ir-complexes [19].

In initial model studies using neopentyl chloride and a commercially available solution of KPPh_2_ in THF we obtained high yields of neopentyldiphenylphosphine under reflux conditions [19]. Encouraged by these results, a series of chloroalkyl-substituted oxazolines **2** were prepared from 3-chloropivaloyl chloride (**3**) by amide formation and subsequent cyclization with the Burgess reagent (methyl *N*-(triethylammoniumsulfonyl)carbamate) using standard procedures (Scheme 2). Subsequent nucleophilic substitution with KPP_2_ in refluxing THF proved to be much faster than the analogous reaction with neopentyl chloride. After 6 hours, the KPPh_2_ was fully converted to the desired phosphinooxazoline, as evidenced by the ^31^P NMR spectrum, which showed only the product signal. The reaction also proceeded well with other diarylphosphides such as KP(*o*-Tol)_2_ or KP(3,5-MePh)_2_ prepared in situ from the secondary phosphines by deprotonation with KH in refluxing THF. The NeoPHOX ligands, which were obtained by this route in high overall yield, proved to be air and moisture stable and could be obtained in analytically pure form by simple filtration through silica gel [19]. Complexation with [Ir(COD)Cl]_2_ and subsequent anion exchange with NaBAr_F_ (sodium tetrakis[3,5-bis(trifluoromethyl)phenyl]borate) led to the corresponding iridium precatalysts in high yields. These complexes as well showed high stability against oxygen and moisture and could be stored in air for several months without notable decomposition.

**Figure 2:**
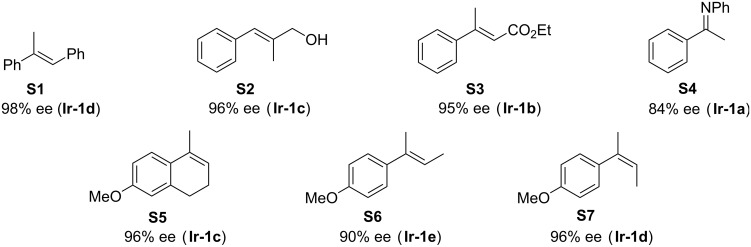
Asymmetric hydrogenation with iridium-NeoPHOX catalysts [19].

As originally assumed, the NeoPHOX complexes **Ir-1a–e** turned out to be highly efficient catalysts. They induced similar and in some cases even higher enantioselectivities compared to the analogous SimplePHOX complexes, as shown by the results obtained in the hydrogenation of a range of test substrates (Figure 2) [19]. Considering the more practicable synthesis, which is well suited for large-scale preparation, and the higher stability of the free ligands, NeoPHOX ligands provide a superior alternative to the SimplePHOX ligands. The only drawback these compounds share with many state-of-the-art oxazoline-based ligands is the high cost of *tert*-leucinol as starting material, which reduces their potential for industrial-scale application. We therefore thought of ways to replace the *tert*-butyloxazoline ring by an equally effective oxazoline unit derived from a less expensive precursor.

**Figure 3:**
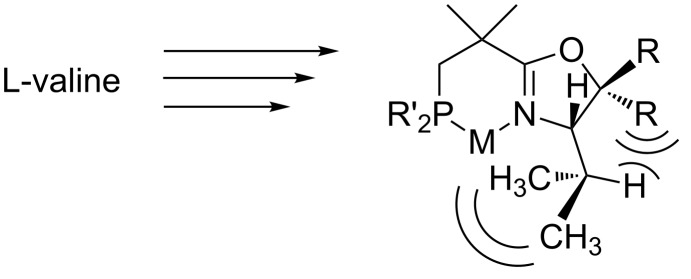
Employing L-valine as a starting material for C5 substituted oxazoline.

An option, which had been previously used in the development of PHOX ligands [23–27], is to replace the *tert*-butyl group by an isopropyl group and at the same time introducing two bulky substituents at C(5) (see Figure 3). Ligands of this type are accessible from valine, which is much less expensive than *tert*-leucine. The steric hindrance exerted by the geminal substituents at C(5) is expected to direct the isopropyl methyl groups toward the coordination sphere, creating a steric environment which resembles that of a *tert*-butyloxazoline ligand.

**Scheme 3:**
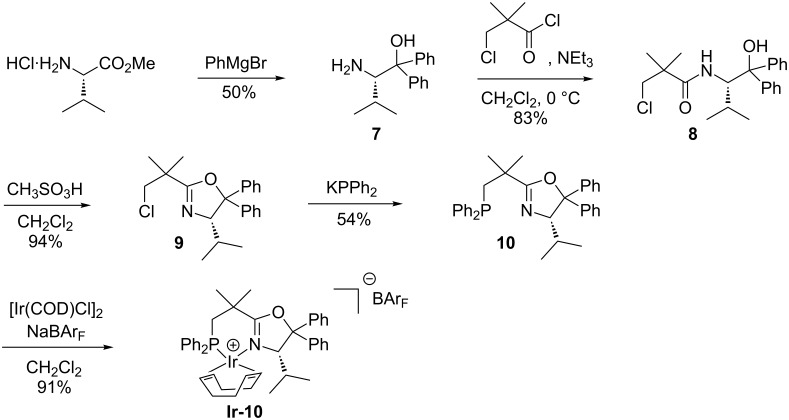
Synthesis of a C(5)-disubstituted NeoPHOX-Ir complex.

To test the viability of this approach, we synthesized the valine-derived NeoPHOX ligand **10** with two geminal phenyl substituents at C(5) according to the route shown in Scheme 3.

Unfortunately, the iridium complex **Ir-10** prepared from this ligand proved to be an inefficient catalyst. The enantioselectivities in the hydrogenation of various test substrates were much lower than those induced by the *tert-*butyloxazoline analog with the exception of the result obtained with the allylic alcohol **S2** (Table 1). Surprisingly, the presence of two geminal phenyl substituents at C(5) had a negative impact on the enantioselectivity, as shown by the substantially higher ee values induced by the analogous catalyst **Ir-1a** [28] lacking substituents at C(5).

**Table 1:** Hydrogenation results employing 1st generation NeoPHOX catalysts.^a^

	**S1** ee [%] yield [%]	**S5** ee [%] yield [%]	**S2** ee [%] yield [%]	**S3** ee [%] yield [%]

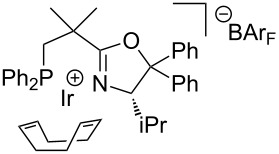 **Ir-10**	19 (*R*) 93	35 (*S*) >99	84 (–) >99	63 (*R*) 41
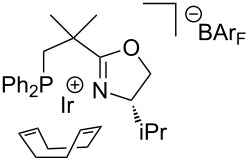 **Ir-1a**	74 (*R*) >99	53 (*S*) 89	88 (–) >99	85 (*R*) >99
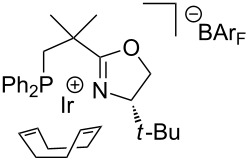 **Ir-1b**	97 (*R*) >99	92 (*S*) >99	83 (–) >99	95 (*R*) >99

^a^Reaction conditions: 50 bar, 2 h, 1 mol % catalyst, 0.1 mmol substrate, 0.5 mL CH_2_Cl_2_.

**Figure 4:**
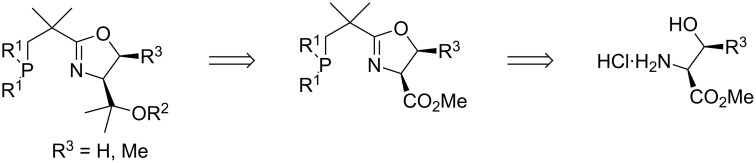
Retrosynthetic analysis for NeoPHOX ligands derived from serine and threonine.

Based on these negative results we chose a new approach based on serine or threonine as starting materials (Figure 4). The carboxyl group of these amino acids serves as surrogate for the *tert*-butyl group of *tert*-leucine, which can be converted to a sterically demanding substituent by double addition of Grignard or alkyllithium reagents and subsequent protection of the resulting tertiary alcohol. An attractive feature of this approach is that by proper choice of the alkylmetal reagent and the protecting group, the steric properties of the ligand can be optimized for a specific application. PHOX ligands of this type have been previously prepared from serine and successfully used in iridium-catalyzed hydrogenation [29–30].

### Synthesis of serine- and threonine-derived NeoPHOX ligands

**Scheme 4:**
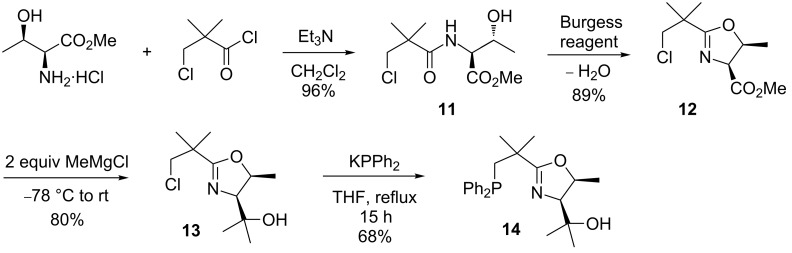
Revisited synthetic strategy for the preparation of a threonine-based NeoPHOX ligand.

The synthesis that led to the NeoPHOX ligand **14** in 46% overall yield is shown in Scheme 4. In the first step threonine methyl ester hydrochloride was converted to the desired amide **11** in 96% yield. Subsequent cyclization with the Burgess reagent afforded the oxazoline **12** in 89% yield with clean inversion of configuration at the hydroxy-substituted carbon atom. Double addition of methylmagnesium chloride and subsequent introduction of the diphenylphosphine unit proceeded well in 68% yield following the procedures worked out for the 1st generation NeoPHOX ligands.

**Scheme 5:**
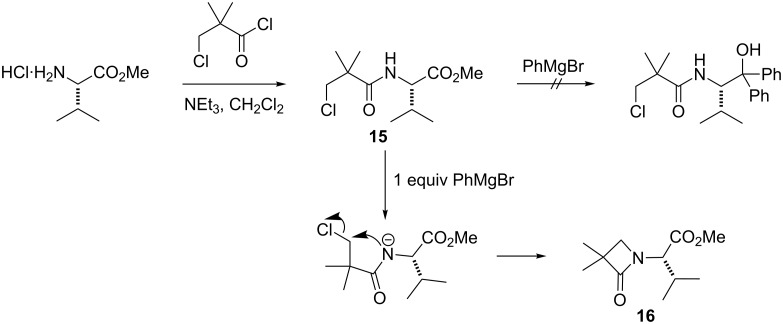
Undesired β-lactam formation.

Alternative strategies involving Grignard addition to the threonine methyl ester or N-protected derivatives were also briefly investigated, but failed. Grignard addition to the chloro amide **11** was not attempted because earlier studies with chloro amide **15** had shown that an undesired β-lactam **16** was formed upon treatment with Grignard reagents (Scheme 5) [28].

**Scheme 6:**
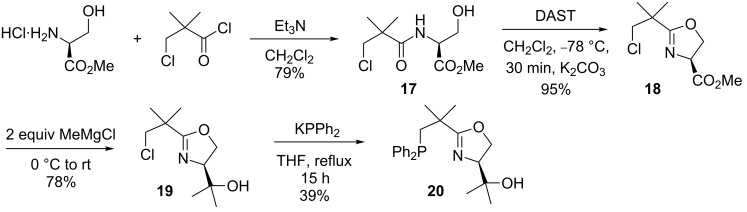
Synthetic strategy for the synthesis of the serine-derived NeoPHOX ligand.

All attempts to prepare analogous serine-derived NeoPHOX ligands by the established route for the synthesis of the threonine-derived NeoPHOX ligand **14** led to unexpected problems (Scheme 6). Oxazoline formation with the Burgess reagent did not produce the desired oxazoline (mixture of unidentified products). However, eventually an alternative method using diethylaminosulfurtrifluoride (DAST) [31] was found that afforded the chloroalkyloxazoline **18** in 95% yield after short reaction times at −78 °C.

Treatment of this product with methylmagnesium chloride under the same conditions used for the threonine derivative **12** (Scheme 4), led to an unidentified mixture of products. Surprisingly, when the Grignard reagent was added at 0 °C instead of −78 °C and reaction mixture subsequently warmed up to room temperature, the desired product **19** was formed in 78% yield. After having resolved these issues, the desired serine-based NeoPHOX ligand **20** was obtained in 23% overall yield from serine methylester hydrochloride.

**Scheme 7:**
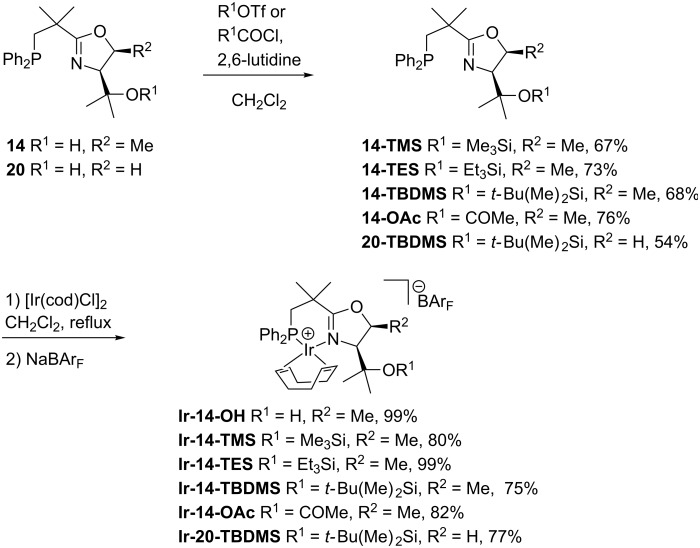
Derivatization of the 2nd generation NeoPHOX ligands and formation of their iridium complexes.

Next we converted the tertiary alcohols **14** and **20** to a range of silylated and acetylated derivatives in order to evaluate the influence of sterically and electronically different substituents on the enantioselectivity and catalytic activity of the corresponding iridium complexes. Initial attempts to introduce a silyl or acetyl group by deprotonation with potassium hydride and subsequent reaction with silyl triflates or acetyl chloride failed. The NeoPHOX ligand **14** showed no reaction under these conditions, although this method had been successfully used for the alkylation and acylation of the tertiary alcohol function of analogous serine derived PHOX ligands [30]. Among various amines, 2,6-lutidine was finally identified as a suitable base for conversion to the desired derivatives in 67–76% yields (Scheme 7). From these ligands a library of iridium catalysts was prepared by complexation with bis(1,5-cyclooctadiene)diiridium(I) dichloride, followed by anion exchange with NaBAr_F_.

### Comparative hydrogenation studies with iridium catalysts derived from 1st and 2nd generation NeoPHOX ligands and serine-based PHOX ligands

A set of seven Ir-NeoPHOX catalysts was evaluated in the asymmetric hydrogenation of three alkenes and acetophenone phenylimine (Table 2). For comparison, the results previously reported for four related serine-based Ir-PHOX complexes are included in the table. Overall, the *tert*-leucine-derived complex **Ir-1b** and complexes **Ir-14-TBDMS** and **Ir-20-TBDMS** with a bulky *tert-*butyldimethylsilyloxy group showed the highest reactivity, resulting in full conversion of all three olefins. On the other hand catalysts **Ir-14-OH** and **ser-PHOX-OMe** bearing a free hydroxy and a methoxy group, respectively, gave very low or no conversion. In the case of complex **ser-PHOX-OMe** [30] it was found that the methoxy function in the oxazoline ring induced deactivation of the catalyst due to coordination to the metal center resulting in a stable hydride-bridged dinuclear complex under hydrogenation conditions. The analogous catalysts **Ir-14-OAc** and **ser-PHOX-OAc** with an acyloxy substituent proved to be more reactive, however, conversion still remained incomplete with olefins **S1** and **S3**. Similar results were obtained with the analogous NeoPHOX complex **Ir-14-OAc** bearing an acetoxy group at the oxazoline ring. Apparently, a polar potentially coordinating substituent near the metal center reduces or can even completely inhibit catalytic activity. The deactivating effect of a coordinating oxygen function could also explain the different conversions observed with the silyl ether-bearing catalysts, decreasing in the series *t-*BuMe_2_Si > Et_3_Si > Me_3_Si. It has been shown that Ir-hydride complexes that are formed as intermediates during hydrogenation are strong Brønsted acids [32], which can cause cleavage of trimethylsilyl ethers under hydrogenation conditions [33]. So partial desilylation liberating a free hydroxy group during hydrogenation may well be the reason for the low conversion obtained with catalyst **Ir-14-TMS** bearing an acid-labile trimethylsilyl ether group.

**Table 2:** Hydrogenation of standard substrates using serine- and threonine-derived NeoPHOX catalysts.

	**S1** ee [%] yield [%]	**S2** ee [%] yield [%]	**S3** ee [%] yield [%]	**S4** ee [%] yield [%]

**Ir-14-OH**	n.d.	41 (+) 10	n.d.	7 (S) 4
**Ir-14-OAc**	87 (*S*) 59	90 (+) 92	90 (*S*) >44	16 (*S*) 73
**Ir-14-TMS**	92 (S) 38	88 (+) 82	82 (*S*) >40	67 (*S*) 5
**Ir-14-TES**	97/^b^96 (*S*) 59/^b^87	89/^b^88 (+) 60/^b^>99	90/^b^90 (*S*) 87/^b^98	68/^b^62 (*S*) 8/^b^32
**Ir-14-TBDMS**	96 (*S*) >99	91 (+) >99	94 (*S*) >99	49 (*S*) 79
**Ir-20-TBDMS**	90 (*S*) >99	84 (+) >99	92 (*S*) >99	77 (*S*) 79
**Ir-1b**	97 (*R*) >99	97% (-) >99	97% (*R*) >99	97 (*R*) >99
**ser-PHOX-OMe** [30]	n.d. 1	57 (+) 9	32 (*S*) 3	5 (R) 2
**ser-PHOX-Bn** [30]	92 (*S*) 27	79 (+) 43	46 (*S*) 34	21 (*S*) 1
**ser-PHOX-OAc** [30]	92 (*S*) 90	91 (+) >99	56 (*S*) 70	56 (*R*) 99
**ser-PHOX-Bz** [30]	92(*S*) 88	92 (+) >99	69 (*S*) 91	53 (*R*) >99

^a^Reaction conditions: 50 bar, 2h, 1 mol % catalyst, 0.1 mmol substrate, 0.5 mL CH_2_Cl_2_; ^b^reaction time 16 h; 
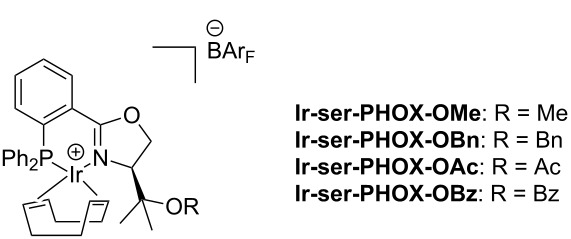

Among the NeoPHOX catalysts, the most reactive complex with a bulky TBDMS group showed also the highest enantioselectivities in the hydrogenation of alkenes **S1**–**3**. It also outperformed the most efficient serine-derived PHOX catalysts **ser-PHOX-OAc** and **ser-PHOX-OBz**. In terms of activity and enantiomeric excess the performance of this catalyst was comparable to the *tert*-leucine-derived complex **Ir-1b**. The analogous serine-based complex **Ir-20-TBDMS** gave somewhat lower enantioselectivities. In the hydrogenation of imine **S4** none of the catalysts shown in Table 2 induced high enantioselectivity.

### Crystal structures of iridium NeoPHOX complexes

**Figure 5:**
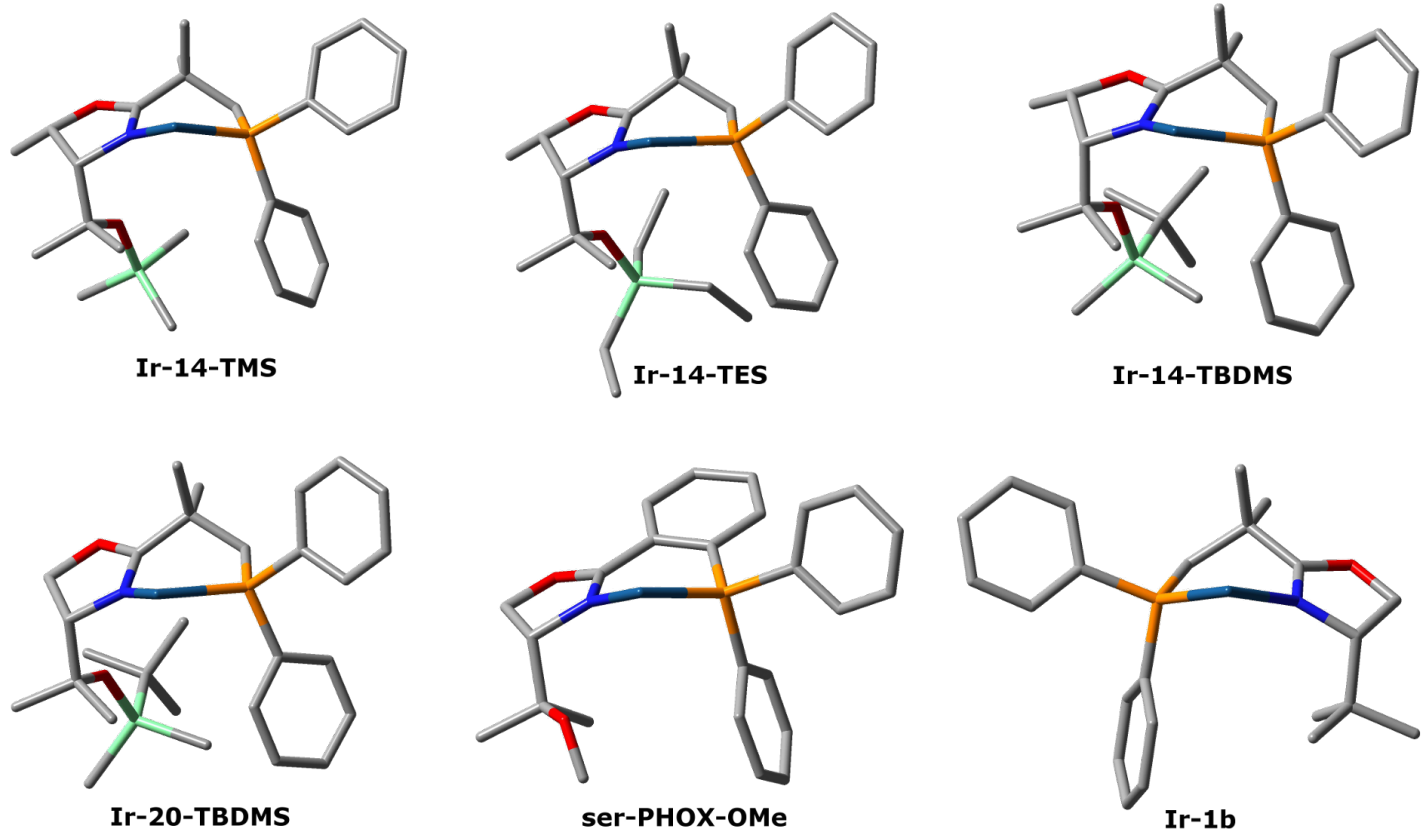
Crystal structures of selected Ir-complexes. Hydrogen atoms, COD and BArF anions were omitted for clarity.

The three dimensional structures of several threonine and serine-derived NeoPHOX iridium complexes were determined by X-ray diffraction and compared with known phosphinooxazoline complexes. We were interested to see whether the differences in steric shielding of the coordination sphere by the protecting groups on the tertiary alcohol function correlated with the hydrogenation results. From the obtained crystal structures, it can be seen that the conformations of the threonine and serine-derived NeoPHOX iridium complexes are essentially the same as those of the *tert*-leucine-derived NeoPHOX complex with respect to the geometry of the 6-membered iridacycle, which retains a boat-shaped conformation (Figure 5). No notable divergence in the steric environment of the iridium center in the four silyloxy-bearing NeoPHOX complexes could be observed that would explain the difference in enantioselectivity induced by these catalysts.

In contrast to the methoxy-bearing serine-derived PHOX complex **ser-PHOX-OMe**, the protected tertiary alcohol group in these complexes does not point towards the iridium center. The interaction of the ether oxygen atom with the iridium center was the reason for the lack of reactivity of this serine-based PHOX complex. In case of the silyl ethers the oxygen atom is strongly shielded preventing coordination to the iridium center.

### Palladium-catalyzed allylic substitution

**Scheme 8:**
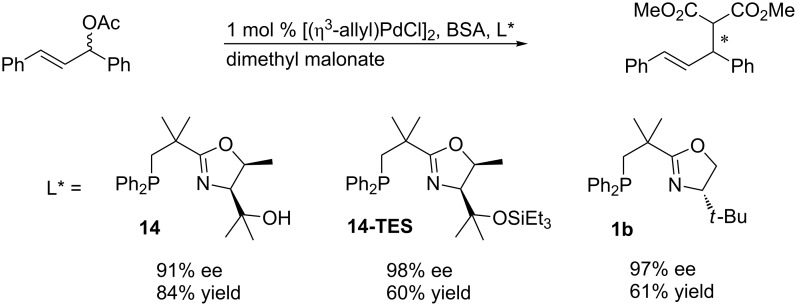
Asymmetric palladium-catalyzed allylic substitution with *rac*-(*E*)-1,3*-*diphenylallyl acetate*.*

As phosphinooxazoline ligands were originally designed for asymmetric palladium-catalyzed allylic substitutions, we tested the new NeoPHOX ligands in this reaction as well. For comparison with state-of-the-art ligands, we chose the standard test reaction of (*E*)-1,3*-*diphenylallylacetate with dimethyl malonate as nucleophile (Scheme 8).

The two catalysts derived from ligand **14** with a free hydroxy group and the corresponding triethylsilyl-protected derivative both performed well affording enantioselectivities of 90% and 98% ee, respectively. Ligand **14-TES** was even more effective than the corresponding *tert*-leucine-derived NeoPHOX ligand **1b** (97% ee).

**Scheme 9:**
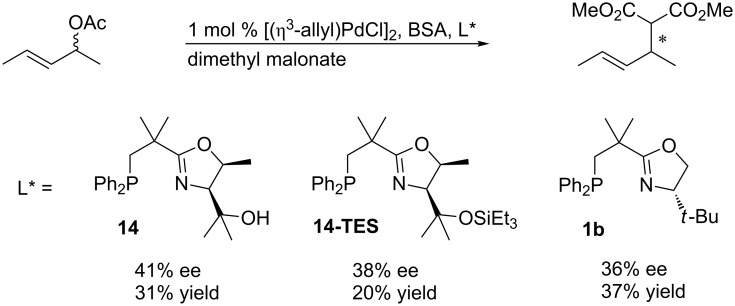
Asymmetric palladium-catalyzed allylic substitution with *rac*-(*E*)-1,3*-*dimethylallyl acetate*.*

Next, we also tested (*E*)-1,3*-*dimethylallyl acetate as substrate (Scheme 9). All three tested catalysts gave disappointing results. The yield was low in all cases and the enantiomeric excess reached only 41% for the best ligand **14**. Interestingly, the sterically more demanding ligand **14-TES** did not lead to higher enantioselectivity as for 1,3*-*diphenylallyl acetate.

**Scheme 10:**
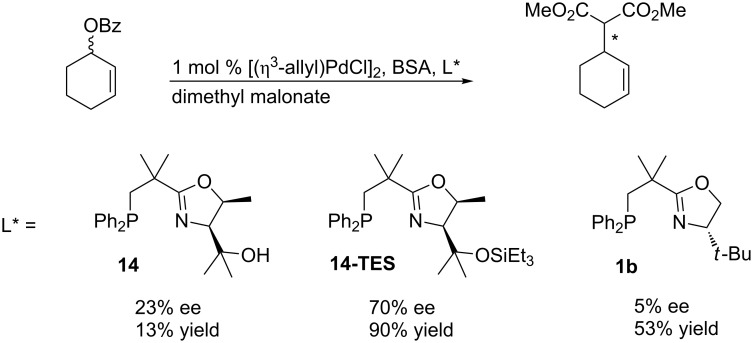
Asymmetric palladium-catalyzed allylic substitution with a cyclic substrate.

Finally, the ligands were also tested on a more demanding cyclic substrate (Scheme 10). In the allylic substitution with cyclohex-2-en-1-yl benzoate a notable enantiomeric excess of 70% was achieved using ligand **14-TES**, in striking contrast to the extremely low enantioselectivities reported for analogous PHOX ligands [4]. Using ligand **14** with a free hydroxy group the result was less satisfying. Not only the yield dropped to 53% but also the ee was substantially lower than with the triethylsilyl analog **14-TES**. For NeoPHOX ligand **1b** the enantiomeric excess was even lower (5%). These results demonstrate that the 2nd generation NeoPHOX ligands possess potential for palladium-catalyzed allylic substitutions.

## Conclusion

The most successful, most widely applied oxazoline-based N,P ligands are derived from the unnatural amino acid *tert*-leucine. The high price of this starting material is an impeding factor for large-scale applications. The NeoPHOX ligands derived from threonine provide an attractive alternative in this respect. Threonine as a chiral building block is available in both enantiomeric forms at a moderate price. The most effective ligand in this series, bearing a bulky CMe_2_OSiMe_2_*t*-Bu group at the stereogenic center, induced excellent enantioselectivities in the Ir-catalyzed asymmetric hydrogenation of olefins, with ee values in the same range as those reported for the best N,P ligands including the *tert*-leucine-derived NeoPHOX analog. In the asymmetric Pd-catalyzed allylic substitution as well, promising enantioselectivities were obtained, indicating a considerable potential for this ligand class in asymmetric catalysis.

A further notable feature of these ligands is their flexible synthesis, which allows easy variation of the substituent at the stereogenic center. Using different Grignard reagents in the addition to the ester group of a late stage intermediate, an array of tertiary alcohol derivatives is available. The subsequent silylation or acylation of the hydroxy group gives access to a library of diverse NeoPHOX ligands. In this way the steric size and the coordination ability of the substituent at the stereogenic center can be tuned for a specific application.

## Supporting Information

File 1 Experimental procedures and characterization data of all compounds and copies of ^1^H and ^13^C NMR spectra of selected molecules.
