# Supplementary material for: NeoPHOX – a structurally tunable ligand system for asymmetric catalysis
Source: Beilstein J Org Chem. 2016 Jun 13;12:1185–95. doi: 10.3762/bjoc.12.114 (PMC4979954; doi:10.3762/bjoc.12.114)
Supplement: File 1 — Experimental procedures and characterization data of all compounds and copies of 1H and 13C NMR spectra of selected molecules. [file Beilstein_J_Org_Chem-12-1185-s001.pdf]

Supporting Information

for

NeoPHOX – a structurally tunable ligand system for asymmetric catalysis

Jaroslav Padevět, Marcus G. Schrems, Robin Scheil and Andreas Pfaltz*

Address: Department of Chemistry, University of Basel, St. Johanns-Ring 19, CH-4056 Basel, Switzerland

Email: Andreas Pfaltz - andreas.pfaltz@unibas.ch

* Corresponding author

Experimental procedures and characterization data of all compounds and copies of ^{1}H and ^{13}C NMR spectra of selected molecules

1. General

Working techniques and reagents

Synthetic procedures involving manipulation under inert atmosphere were performed in dried glassware under positive argon pressure using standard Schlenk techniques. Moisture and air-sensitive compounds were handled in a glove box (MBraun Labmaster 130).

Commercially available reagents were purchased from Acros, Aldrich, Fluorochem, Strem and used without further purification. Triethylamine was distilled from calcium hydride, 2,6-lutidine was distilled under reduced pressure prior to use.

Anhydrous solvents were obtained by distillation from sodium/benzophenone (diethylether, pentane, tetrahydrofuran, toluene) or purification over activated alumina columns under

nitrogen (PureSolv, Innovative Technology Inc.) or obtained from Aldrich or Fluka in septum-sealed bottles under inert atmosphere and over molecular sieves. Oxygen-free solvents were prepared by freeze-pump-thaw degassing.

Column chromatography was performed on silica gel 60 (0.040–0.063 mm) or neutral alumina obtained from Aldrich or Merck. Reagents were of technical grade and were distilled prior to use.

Analytical methods

NMR Spectroscopy: NMR spectra were recorded either on a Bruker Avance 400 (400 MHz, BBO probe head) or a Bruker Avance DRX 500 (500 MHz, BBO or BBI probe heads) NMR spectrometer. Chemical shifts δ are given in ppm and they are referenced for $CDCl_3$ to 7.26 ppm (^{1}H NMR) and 77,16 ppm (^{13}C NMR) for C_6D_6 to 7.16 ppm (^{1}H NMR) and 128.1 ppm (^{13}C NMR) and for THF-d_8 to 3.58 ppm (^{1}H NMR) or to internal standard TMS 0 ppm. ^{31}P NMR spectra were calibrated to an external standard of phosphoric acid (85%) to 0 ppm. ^{19}F NMR spectra were calibrated to the chemical shift of the most downfield isotopomer of external $CFCl_3$ to 0 ppm. ^{11}B NMR spectra were calibrated to 0 ppm with $BF_3{\cdot}Et_2O$ as an external standard. The assignment of ^{1}H and ^{13}C NMR signals was accomplished with help of DEPT135 NMR experiments or by using 2D NMR experiments (COSY, HMQC, HSQC, HMBC). Multiplets were assigned as s (singlet), d (doublet), dd (doublet of doublet), t (triplet), q (quartet), m (multiplet) and br s (broad singlet).

Mass spectrometry: EI (Electron Impact) and FAB (Fast atom bombardment) mass spectra were recorded by Dr. Heinz Nadig (Department of Chemistry, University of Basel). Electron Impact ionization spectra were recorded on a VG70-250 spectrometer and Fast Atom Bombardment spectra were recorded on a Finnigan MAR312 system with 3-nitrobenzyl alcohol (NBA) as matrix. Electron spray ionization (ESI) was measured on a Varian 1200L

Triple Quad MS/MS spectrometer with the sample concentrations between 10^{-4} and 10^{-5} M (40 psi nebulizing gas, 4.9 kV spray voltage, 18 psi drying gas at 200 °C, 38–75 V capillary voltage, 1300–1500 V detector voltage). MALDI (Matrix-assisted laser desorption ionization) spectra were recorded on a Voyager-DE-Pro or Bruker Microflex system with *p*-nitroaniline or 2,5-dihydroxybenzoic acid as matrices. The signals are given in mass-to-charge ratios (*m/z*) with the relative intensities in brackets.

Elemental analysis: Elemental analyses were measured by Mr. W. Kirsch (Department of Chemistry, University of Basel) on a Lenco CHN-900 system.

Melting points: Melting points were determined on a Büchi 535 melting point apparatus and are uncorrected.

Optical rotations: Optical rotations were measured on a Perkin Elmer Polarimeter 341 (l = 1 dm) at 20 °C at 589 nm (sodium lamp). The concentration c is given in g/100 mL.

Infrared spectroscopy: Infrared spectra were recorded on a Perkin Elmer 1600 series FTIR spectrometer or on a Shimadzu FTIR-8400S spectrometer (Golden Gate ATR). Liquid samples were measured as a thin layer between two sodium chloride plates and solid samples were compressed into potassium bromide pellets. The absorption bands are given in wavenumbers ($\tilde{\nu}$ [cm^{-1}]). The peak intensity is given as s (strong), m (medium), w (weak). The index br stands for broad.

Gas chromatography: Gas chromatograms were recorded on Carlo Elba HRGC Mega2 Series 800 (HRGS Mega 2) instruments. Separations on an achiral stationary phase were performed on a Restek Rtx-1710 column (30 m × 0.25 mm × 0.25 µm); for separations on a chiral stationary phase β- and γ-cyclodextrin columns (30 m × 0.25 mm × 0.25 µm) were used.

Gas chromatography with mass spectrometric detection (GC–MS): A HP6890 gas chromatograph with HP5970A mass detector (EI) was used. Column: Macherey-Nagel OPTIMA1 Me2Si (25 m × 0.2 mm × 0.35 µm, 20 psi, split ca. 20:1, carrier gas: 1 mL/min helium). Shimadzu GC-MS-QP2010 SE; column: Rtx-5MS (30 m × 0.25 mm × 0.25 µm, 100 kPa, split ca. 40:1, carrier gas: 3 mL/min).

High-performance liquid chromatography (HPLC): A Shimadzu system with SCL-10A system controller, CTO-10AC column oven, LC10AD pump system, and DGU-14a degasser was used. Columns: Chiracel OD-H, OJ-H, AD-H (4.6 × 250 mm; Daicel Chemical Industries).

2. Synthetic procedures and analytical data

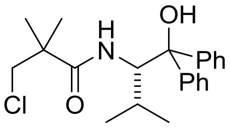

8: (*S*)-3-Chloro-*N*-(1-hydroxy-3-methyl-1,1-diphenylbutan-2-yl)-2,2-dimethylpropanamide

The aminoalcohol **7** (1.03 g, 4.03 mmol, 1.00 equiv) was dissolved in 10 mL of dry CH_2Cl_2, NEt_3 (0.61 mL, 4.43 mmol, 1.10 equiv) was added under stirring and the mixture was cooled to 0 °C. Then, 3-chloropivaloyl chloride (625 mg, 4.03 mmol, 1.00 equiv) dissolved in 10 mL of dry CH_2Cl_2 was added drop wise under stirring and the cooling bath was removed. The mixture was stirred over night at rt and the reaction quenched by the addition of 10 mL of a 1 M HCl solution. The phases were separated and the aqueous phase was extracted with CH_2Cl_2 (2 × 10 mL). The combined organic phases were washed with saturated $NaHCO_3$ solution (10 mL). The aqueous phase was back-extracted with CH_2Cl_2 (2 × 10 mL). The combined organic phases were washed with brine, dried over Na_2SO_4 and the solvent was removed leaving a yellow solid. To the crude product were added 10 mL of ethyl acetate and 40 mL of

hexanes. The suspension was heated to reflux and additional ethyl acetate (12 mL) was added until all solids dissolved. The hot solution was filtered and the filter was rinsed with 20 mL of hot hexanes. The solution was cooled in a freezer (−20 °C) over night. A first batch of product was collected as colorless crystals (830 mg, 2.22 mmol, 50%, mp: 182–183 °C). The mother liquor was concentrated and 10 mL of hexanes added to the solid. The obtained mixture was heated to reflux. When the mixture was cooled to rt again the mother liquor was decanted and the process repeated by addition of 5 mL of hexanes. The remaining white solid (418 mg, 1.12 mmol, 28%) was pure based on ^{1}H NMR analysis and had a mp of 164–165 °C. The combined yield was 1.25 g (3.34 mmol, 83%).

[$C_{22}H_{28}ClNO_2$], (373.92 g/mol)

M.p.: 182-183 °C. **^{1}H-NMR** (400.1 MHz, $CDCl_3$, 300 K) δ (ppm) 7.59-7.44 (m, 4H, HC_{Ar}), 7.35-7.30 (m, 2H, HC_{Ar}), 7.28-7.19 (m, 3H, HC_{Ar}), 7.18-7.12 (m, 1H, HC_{Ar}), 6.24 (d, 1H, J = 9.4 Hz, N*H*), 4.97 (d, 1H, J = 10.6 Hz, NC*H*), 3.61 (dd, 1H, J = 0.9 Hz, J = 10.7 Hz, $ClCH_2$), 3.34 (dd, 1H, J = 1.0 Hz, J = 10.7 Hz $ClCH_2$), 2.84 (s, 1H, O*H*), 1.92 (m, 1H, $CH(CH_3)_2$), 1.12 (s, 3H, $C(CH_3)_2$), 0.95 (d, 3H, J = 0.9 Hz, $C(CH_3)_2$), 0.93 (d, 6H, J = 6.8 Hz, 2×$CH(CH_3)_2$). **^{13}C{^{1}H}-NMR** (100.6 MHz, $CDCl_3$, 300 K) δ (ppm) 174.3 (*C*=O), 145.8 (C_{Ar}), 145.4 (C_{Ar}), 128.5 (HC_{Ar}), 128.3 (HC_{Ar}), 127.0 (HC_{Ar}), 126.9 (HC_{Ar}), 125.3 (HC_{Ar}), 125.2 (HC_{Ar}), 82.3 (*C*OH),57.9 (N*C*H), 52.6 (CH_2Cl), 44.3 ($C(CH_3)_2$), 28.8 ($CH(CH_3)_2$), 23.6 (CH_3), 23.0 (CH_3), 22.8 (CH_3), 17.9 (CH_3). **MS** (EI) m/z (%): 554 (10), 374 ($[M+H]^+$, 10), 356 (100), 238 (21), 221 (20), 190 (51), 167 (20), 105 (27), 91 (56), 72 (39), 55 (28). **IR** ($\tilde{\nu}$ [cm^{-1}]) 3434m, 2964m, 2936w, 2877w, 1631s, 1532s, 1497w, 1480w, 1467w, 1447m, 1391m, 1365m, 1339w, 1323w, 1288w, 1277w, 1239w, 1219w, 1170m, 1145w, 1127m, 1099w, 1064m, 1045w, 1036w, 949w, 918m, 889m, 743s, 727m, 694s, 664m, 642m, 626m.

$[\alpha]_D^{20}$ −36.0 (*c* 0.95, $CHCl_3$). **HRMS** (+ESI, *m/z*) for $[C_{22}H_{28}ClNO_2+Na]^+$: calc.: 396.1706; found: 396.1708.

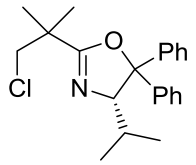

9: (*S*)-2-(1-Chloro-2-methylpropan-2-yl)-4-isopropyl-5,5-diphenyl-4,5-dihydrooxazole

Amide **8** (650 mg, 1.74 mmol) was dissolved in 10 mL of $CHCl_3$. Methanesulfonic acid was added drop wise (0.3 mL) and the mixture heated to reflux. A solid extractor containing 4 g of activated molecular sieves (4 Å) was used to remove water. After 3 h of reflux, the mixture was cooled to 0 °C and 10 mL of a saturated $NaHCO_3$ solution were added. The aqueous phase was extracted with CH_2Cl_2 (2 × 10 mL), the combined organic phases were washed with brine and dried over Na_2SO_4. The solvent was removed and the crude product was dried under high vacuum to give 615 mg of a slightly yellow oil (99% yield, pure by ^{1}H NMR). The oil was dissolved in 5 mL of hexanes, filtered and the filter was rinsed with 10 mL of hexanes. The solvent was removed and the product was dried under high vacuum to give 584 mg (1.64 mmol, 94%) of product **9** as yellow oil.

$[C_{22}H_{26}ClNO]$, (355.90 g/mol)

^{1}H-NMR (400.1 MHz, $CDCl_3$, 300 K): δ(ppm) 7.46-7.43 (m, 2H, H_{Ar}), 7.35-7.21 (m, 8H, H_{Ar}), 4.61 (d, 1H, J = 4.4 Hz, NC*H*), 3.76 (d, 1H, J = 10.8 Hz, ClCH_2), 3.68 (d, 1H, J = 10.8 Hz, ClCH_2), 1.78-1.67 (m, 1H, C*H*(CH_3)$_2$), 1.37 (s, 3H, C(CH_3)$_2$), 1.35 (s, 3H, C(CH_3)$_2$), 0.94 (d, 3H, J = 6.8 Hz, CH(CH_3)$_2$), 0.58 (d, 3H, J = 6.6 Hz, CH(CH_3)$_2$). **^{13}C{^{1}H}-NMR** (100.6 MHz, $CDCl_3$, 300 K): δ(ppm) 167.7 (*C*=N), 145.6 (C_{Ar}), 140.5 (C_{Ar}), 128.2 (HC_{Ar}), 127.7 (2 lines, HC_{Ar}), 127.0 (HC_{Ar}), 126.9 (HC_{Ar}), 126.1 (HC_{Ar}), 92.4 (O*C*(C_6H_5)$_2$), 79.1 (N*C*H), 52.3 (Cl*C*H_2), 38.9 (*C*(CH_3)$_2$), 30.2 (*C*H(CH_3)$_2$), 23.7 (2 lines, *C*H_3), 21.8 (*C*H_3),

16.7 (CH_3). **MS** (EI) m/z (%): 356 ($[M+H]^+$, 100), 221 (13), 173 (37), 105 (20), 77 (12), 91 (16), 55 (9). **IR** ($\tilde{\nu}$ [cm^{-1}]) 3058w, 2957m, 2929m, 2871m, 1668s, 1599w, 1584w, 1492s, 1468s, 1447s, 1386s, 1366m, 1340w, 1335w, 1313w, 1290w, 1228w, 1175m, 1157w, 1139m, 1115s, 1089m, 1060w, 1035m, 1002w, 973s, 956s, 926m, 907w, 754s, 700s, 678w, 625m, 609m. $[\alpha]_D^{20}$ −313.3 (*c* 0.93, $CHCl_3$). **HRMS** (+ESI, *m/z*) for $[C_{22}H_{26}ClNO+H]^+$: calc.: 356.1781; found: 356.1783.

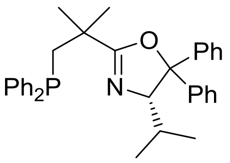

10: (*S*)-2-(1-(Diphenylphosphino)-2-methylpropan-2-yl)-4-isopropyl-5,5-diphenyl-4,5-dihydrooxazole

A dry J.-Young tube was charged with oxazoline **9** (200 mg, 562 μmol, 1.00 equiv) and sealed under argon (3 ×). A 0.5 M solution of $KPPh_2$ in THF (1.4 mL, 0.70 mmol, 1.25 equiv) was added and the mixture was stirred at 80 °C for 6 h. The solution was cooled to 0 °C and a few drops of water were added until the solution became colorless. Then 10 mL of a saturated NH_4Cl solution were added followed by 20 mL of MTBE. The phases were separated and the aqueous phase was extracted with 2 × 10 mL of MTBE. The combined organic phases were washed with brine and dried over Na_2SO_4. Silica gel was added and the solvent was removed on a rotavap. The compound was purified by filtration through silica gel (h×d, 11 cm×3 cm, pentane/ethyl acetate, 20/1). The product was obtained as a colorless wax (154 mg, 0.31 mmol, 54%).

$[C_{34}H_{36}NOP]$, (505.63 g/mol)

^{1}H-NMR (400.1 MHz, $CDCl_3$, 300 K): δ (ppm) 7.51-7.40 (m, 6H, H_{Ar}), 7.35-7.19 (m, 14H, H_{Ar}), 4.58 (d, 1H, J = 4.6 Hz, NCH), 2.56 (d, 2H, J = 3.4 Hz, PCH_2), 1.79-1.70 (m, 1H, CH(CH_3)$_2$), 1.33 (s, 6H, C(CH_3)$_2$), 0.92 (d, 3H, J = 6.8 Hz, CH(CH_3)$_2$), 0.63 (d, 3H,

J = 6.5 Hz, CH(CH_3)$_2$). **^{13}C{^{1}H}-NMR** (100.6 MHz, $CDCl_3$, 300 K): δ(ppm) 170.4 (d, J = 4 Hz, C=N), due to the complexity of the aromatic region the C_{Ar} and HC_{Ar} peaks are listed without consideration of C-P couplings, 145.8, 140.9, 140.0 (2 lines), 139.9, 139.8, 133.1, 133.0, 132.9, 132.8, 128.4, 128.3, 128.3, 128.3, 128.3, 128.2, 128.1, 127.6, 127.6, 127.0, 126.9, 126.3, 92.2 (OC(C_6H_5)$_2$), 79.1 (NCH), 40.4 (d, J = 17 Hz, PCH$_2$), 37.1 (d, J = 18 Hz, C(CH_3)$_2$), 30.2 (CH(CH_3)$_2$), 27.0 (d, J = 10 Hz, C(CH$_3$)$_2$), 26.8 (d, J = 11 Hz, C(CH$_3$)$_2$), 21.8 (CH(CH$_3$)$_2$), 17.0 (CH(CH_3)$_2$). **^{31}P-NMR** (162.0 MHz, $CDCl_3$, 300 K): δ(ppm) −26.9. **MS** (+ESI) m/z (%): 506 ([M+H]$^+$, 100). $[\alpha]_D^{20}$ −198.0 (c 0.55, $CHCl_3$). **HRMS** (+ESI, m/z) for [$C_{34}H_{36}NOP$+H]$^+$: calc.: 506.2613; found: 506.2613.

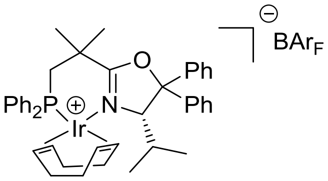

Ir-10: (S)-2-(1-(Diphenylphosphino)-2-methylpropan-2-yl)-4-isopropyl-5,5-diphenyl-4,5-dihydrooxazole-η^4-(1,5-cyclooctadiene)-iridium(I) tetrakis-[3,5-bis(trifluoromethyl)phenyl)-borate

The ligand (120 mg, 237 µmol, 1.00 equiv) and the iridium precursor (87.7 mg, 131 µmol, 0.50 equiv) were placed in a J.-Young-tube, the atmosphere was exchanged to argon and 2 mL of dry CH_2Cl_2 were added. The solution was heated to 50 °C for 45 min and then cooled to rt. $NaBAr_F$ (252 mg, 284 µmol, 1.20 equiv) were added and the solution was stirred for 30 min. Silica gel was added and the solvent was removed on a rotovap. The complex was purified by filtration over silica (40 g, h×d, 12 cm×3 cm) using 200 mL of MTBE followed by 200 mL of CH_2Cl_2. The complex was obtained as an orange solid (359 mg, 215 µmol, 91%).

^{1}H-NMR (500.1 MHz, CD_2Cl_2, 300 K): δ(ppm) 7.86 (dd, 2H, J = 7.4 Hz, J = 11.1 Hz, H_{Ar}), 7.75 (s, 8H, $H_{ArF\text{-}o}$), 7.60-7.52 (m, 9H, $H_{ArF\text{-}p}$, H_{Ar}), 7.48 (t, 2H, J = 7.7 Hz, H_{Ar}), 7.42-7.38

(m, 4H, H_{Ar}), 7.37-7.29 (m, 4H, H_{Ar}), 7.28-7.24 (m, 1H, H_{Ar}), 7.09-7.05 (m, 2H, H_{Ar}), 4.91-4.86 (m, 1H, COD-C*H*), 4.71 (d, 1H, *J* = 1.1 Hz, NC*H*), 4.20-4.15 (m, 1H, COD-CH), 3.68-3.64 (m, 1H, COD-C*H*), 2.71-2.60 (m, 3H, PCH_2, COD-CH_2), 2.55 (dd, 1H, *J* = 8.0 Hz, *J* = 15.3 Hz, COD-CH_2), 2.45-2.40 (m, 1H, COD-C*H*), 2.16-2.08 (m, 5H, C(CH_3)$_2$, 2×COD-CH_2), 1.96-1.82 (m, 5H, C(CH_3)$_2$, C*H*(CH$_3$)$_2$, COD-CH_2), 1.73 (td, 1H, *J* = 9.1 Hz, *J* = 13.5 Hz, COD-CH_2), 1.66-1.58 (m, 1H, COD-CH_2), 1.37-1.31 (m, 1H, COD-CH_2), 1.10 (d, 3H, *J* = 7.1 Hz, CH(CH_3)$_2$), −0.04 (d, 3H, *J* = 6.8 Hz, CH(CH_3)$_2$). **^{13}C{^{1}H}-NMR** (125.8 MHz, CD_2Cl_2, 300 K): δ(ppm) 176.5 (d, *J* = 3 Hz, *C*=N), 161.4 (q, *J* = 51 Hz, $C_{ArF\text{-}i}$), due to the complexity of the aromatic region the C_{Ar} and HC_{Ar} peaks are listed without consideration of C-P couplings, 142.7, 136.1, 135.2, 135.1, 134.5 (H$C_{ArF\text{-}o}$), 132.2 (3×), 131.8, 131.0, 130.9, 130.7 (2×), 129.2, 129.1, 129.0, 128.9, 128.8, 128.6 (qq, *J* = 3 Hz, *J* = 32 Hz, $C_{ArF\text{-}m}$), 128.6 (2×), 128.6, 128.4, 128.3, 128.2 (3×), 128.1, 127.9, 125.0, 123.4, 117.1 (sept, *J* = 4 Hz, H$C_{ArF\text{-}p}$), 94.9 (O*C*(C_6H_5)$_2$), 94.1 (d, *J* = 10 Hz, COD-*C*H), 92.0 (d, *J* = 13 Hz, COD-*C*H), 77.3 (N*C*H), 63.7 (COD-*C*H), 60.9 (COD-*C*H), 38.7 (d, *J* = 2 Hz, *C*(CH$_3$)$_2$), 36.0 (d, *J* = 4 Hz, COD-*C*H$_2$), 33.3 (d, *J* = 32 Hz, P*C*H$_2$), 32.6 (d, *J* = 7 Hz, C(*C*H$_3$)$_2$), 32.0 (d, *J* = 1 Hz, COD-*C*H$_2$), 28.9 (*C*H(CH$_3$)$_2$), 27.8 (d, *J* = 2 Hz, COD-*C*H$_2$), 26.7 (d, *J* = 12 Hz, C(*C*H$_3$)$_2$), 25.6 (COD-*C*H$_2$), 20.6 (CH(*C*H$_3$)$_2$), 13.9 (CH(*C*H$_3$)$_2$). **^{31}P-NMR** (162.0 MHz, CD_2Cl_2, 300 K): δ(ppm) 6.4. **^{19}F{^{1}H}-NMR** (376.5 MHz, CD_2Cl_2, 300 K): δ(ppm) −64.0. **MS** (+ESI) m/z (%) 806 ([M−(BAr$_F$)]$^+$, 100). $[\alpha]_D^{20}$ −52 (*c* 0.29, CHCl$_3$). **Elemental Analysis** for $C_{74}H_{60}BF_{24}IrNOP$ (1669.24 g/mol), calc.: C, 53.25; H, 3.62; N, 0.84; found: C, 53.22; H, 3.61; N, 0.75.

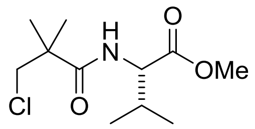

15: (*S*)-Methyl 2-(3-chloro-2,2-dimethylpropanamido)-3-methylbutanoate

L-Valine methyl ester hydrochloride (8.38 g, 50.0 mmol, 1.00 equiv) was suspended in 75 mL of dry CH_2Cl_2 in a three-necked sulfonation flask with mechanical stir bar. The solution was cooled to 0 °C and 20.9 mL of NEt_3 (150 mmol, 3.00 equiv) were added under stirring. To this mixture a solution of 7.75 g of 3-chloropivaloylchloride (50.0 mmol, 1.00 equiv) in 25 mL of dry CH_2Cl_2 was added over 30 min, keeping the temperature between 0 and 10 °C. When the addition was completed, the cooling bath was removed and the reaction mixture was stirred until rt was reached. The mixture was heated to reflux for 1.5 h (brown color), cooled to rt and extracted with 50 mL of water, followed by 50 mL of a 1 M solution of HCl. The combined aqueous phases were extracted with CH_2Cl_2 (2 × 50 mL) and the combined organic phases were washed with 50 mL of a saturated $NaHCO_3$ solution. The aqueous phase was extracted with CH_2Cl_2 (25 mL) and the combined organic phases were washed with 50 mL of brine. The organic phase was dried over Na_2SO_4 and the solvent was removed on a rotovap and the crude product was dried using an oil pump until constant pressure was obtained (10^{-1} mbar). The crude product was obtained as a brown oil (12.44 g) which was taken up in 20 mL of hexanes. Active carbon (300 mg) was added, the mixture was refluxed for 5 min and filtered hot. The filter was rinsed with hot hexanes (5 × 20 mL) and the solution was put in a freezer (−20 °C) over night. The mother liquor was decanted. The product was obtained as a colorless solid containing some brown material as impurity. This mixture was allowed to warm to rt causing the colorless solid to melt, while the impurity remained solid. The liquid was decanted and the flask and the impurity were rinsed with hexane (3×2 mL) and combined with the product. The solvent was removed and the residue dried at 10^{-1} mbar. The product, a slightly yellow liquid, was cooled in a dry-ice/acetone bath and put again under

vacuum to initiate crystallization. Finally, 10.1 g (40.4 mmol, 81%) of an off-white solid were obtained. The impurity and the mother liquor were combined and the mixture was cooled in a dry-ice/acetone bath under shaking. The impurity crystallized first and the cold solution was filtered to remove the impurity. The filter was washed with 4 mL of cold hexanes and the solution was cooled to −78 °C. The mother liquor was decanted and the crystalline product was dried under vacuum to give 1.47 g (5.88 mmol, 12%) as a second batch. Total yield: 11.6 g (46.3 mmol, 93%).

M.p.: 37-38 °C. **^{1}H-NMR** (400.1 MHz, $CDCl_3$, 300 K): δ = 6.25 (d, 1H, *J* = 7.6 Hz, N*H*), 4.56 (dd, 1H, *J* = 4.7 Hz, *J* = 8.5 Hz, NHC*H*COOCH$_3$), 3.73 (s, 3H, COOC*H*$_3$), 3.67 (d, 1H, *J* = 10.8 Hz, ClC*H*$_2$), 3.54 (d, 1H, *J* = 10.8 Hz, ClC*H*$_2$), 2.17 (d sept., 1H, *J* = 4.9 Hz, *J* = 6.9 Hz, C*H*(CH$_3$)$_2$), 1.31 (d, 6H, *J* = 13.6 Hz, C(C*H*$_3$)$_2$), 0.91 (dd, 6H, *J* = 6.9 Hz, *J* = 13.7 Hz, CH(C*H*$_3$)$_2$). **^{13}C{^{1}H}-NMR** (100.6 MHz, $CDCl_3$, 300 K): δ = 174.5 (*C*=O), 172.4 (*C*=O), 57.0 (NH*C*H), 52.5 (*C*H$_2$Cl), 52.1 (COO*C*H$_3$), 44.3 (*C*(CH$_3$)$_2$), 31.3 (C*H*(CH$_3$)$_2$), 23.6 (*C*H$_3$), 23.1 (*C*H$_3$), 18.9 (*C*H$_3$), 17.7 (*C*H$_3$). **MS** (FAB), m/z: 250 ([M+H]$^+$, 100), 190 (37), 137 (11), 91 (19), 55 (11). **IR** ($\tilde{\nu}$ [cm^{-1}]) 3315m, 2964m, 2935m, 2876w, 1738s, 1636s, 1592s, 1568m, 1435m, 1391m, 1369m, 1356m, 1319w, 1294w, 1265w, 1202s, 1148s, 1080w, 1041w, 1001m, 972w, 962w, 926m, 912w, 885w, 847w, 810w, 775m, 727s, 671m, 621m. $[\alpha]_D^{20}$ +9.8 (*c* 1.51, $CHCl_3$). **Elemental Analysis** for $C_{11}H_{20}ClNO_3$ (249.73 g/mol), calc.: C, 52.90; H, 8.07; N, 5.61; found: C, 52.98; H, 7.98; N, 5.49.

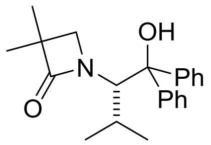

16: (*S*)-1-(1-Hydroxy-3-methyl-1,1-diphenylbutan-2-yl)-3,3-dimethylazetidin-2-one

To a dry sulfination flask equipped with a mechanical stir bar, a reflux condenser, a dropping funnel and a neck closed with a stopper were added 5.00 g (20.0 mmol, 1.00 equiv) of ester **15**. Vacuum was applied and the flask was backfilled with argon. Dry THF (40 mL) was added under stirring and the solution was cooled to 0 °C. Through the dropping funnel a 1 M solution of PhMgBr in dry THF (66 mL, 66.0 mmol, 3.30 equiv) was added over 30 min. The temperature was kept in a range between −5 and 0 °C. After complete addition, the cooling bath was removed and the solution stirred for additional 30 min. Then the solution was heated to reflux for 2 h. After 2 h approximately 50 mL of THF were distilled off and the solution was cooled to 0 °C. A saturated NH_4Cl solution (10 mL) was added drop wise followed by 50 mL of MTBE. The mixture was stirred for 10 min and 40 mL of a 1 M HCl solution were added. The phases were separated and the aqueous phase was washed with 50 mL of MTBE, then again with 25 mL of MTBE. The combined organic phases were washed with 50 mL of saturated $NaHCO_3$ solution and the aqueous phase was extracted with 25 mL of ethyl acetate. The combined organic phases were washed with 50 mL of brine and dried over Na_2SO_4. The solvent was removed on a rotovap. During this process the product started to crystallize. Approximately 100 mL of hexanes were added to the residue and the mixture was heated to reflux. Ethyl acetate was added portion wise to the refluxing mixture until most of the compound had dissolved (approx. 40 mL). The solution was filtered hot and placed in a refrigerator (+5 °C) over night. The mother liquor was decanted and the colorless crystals were rinsed with 6 mL of hexane. A fraction (2.98 g, 7.97 mmol, 40%) was collected having a mp of 183–184 °C.

M.p.: 183-184 °C. **^{1}H-NMR** (400.1 MHz, $CDCl_3$, 300 K): δ = 7.62-7.59 (m, 4H, H_{Ar}), 7.31-7.27 (m, 4H, H_{Ar}), 7.15 (ddt, 2H, J = 3.5 Hz, J = 4.6 Hz, J = 6.0 Hz, H_{Ar}), 6.32 (s, 1H, OH), 4.02 (d, 1H, J = 2.7 Hz, NHCH), 3.21 (d, 1H, J = 4.9 Hz, CH_2), 3.00 (d, 1H, J = 4.9 Hz, CH_2), 2.15 (d sept., 1H, J = 2.7 Hz, J = 6.9 Hz, CH(CH_3)$_2$), 1.20 (s, 3H, C(CH_3)$_2$), 1.12 (d, 3H, J = 6.7 Hz, CH(CH_3)$_2$), 0.90 (d, 3H, J = 7.0 Hz, CH(CH_3)$_2$), 0.64 (s, 3H, C(CH_3)$_2$). **^{13}C{^{1}H}-NMR** (100.6 MHz, $CDCl_3$, 300 K): δ = 175.2 (C=O), 147.2 (C_{Ar}), 144.8 (C_{Ar}), 128.2 (2×HC_{Ar}), 128.0 (2×HC_{Ar}), 126.5 (2×HC_{Ar}), 125.2 (2×HC_{Ar}), 125.0 (2×HC_{Ar}), 80.9 ((C_6H_5)$_2$$C$OH), 70.4 (N$C$H), 58.8 ($CH_2$), 48.2 ($C$($CH_3$)$_2$), 29.6 ($C$H($CH_3$)$_2$), 24.5 (CH($CH_3$)$_2$), 21.2 (C($CH_3$)$_2$), 20.2 (C($CH_3$)$_2$), 19.6 (CH($CH_3$)$_2$). **MS** (FAB, NBA), m/z: 338 ($[M+H]^+$, 42), 320 (100), 292 (19), 250 (15), 221 (19), 126 (43), 105 (29), 55 (21). **IR** ($\tilde{\nu}$ [cm^{-1}]) 3256w, 2968w, 2953w, 2925w, 1707s, 1700s, 1448m, 1418m, 1349m, 1333w, 1293w, 1208w, 1187w, 1094w, 1063m, 1019m, 998w, 962w, 987m, 820m, 754s, 747s, 737m, 700s, 687s, 668s, 652s, 635s. $[\alpha]_D^{20}$ −94.2 (c 1.04, $CHCl_3$). **Elemental Analysis** for $C_{22}H_{27}NO_2$ (337.46 g/mol), calc.: C, 78.30; H, 8.06; N, 4.15; found: C, 78.15; H, 8.00; N, 3.96.

Threonine derived NeoPHOX ligands

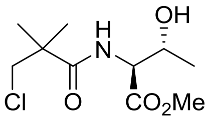

11: (2S,3R)-Methyl 2-(3-chloro-2,2-dimethylpropanamido)-3-hydroxybutanoate

Methylthreonine hydrochloride (5.00 g; 29.5 mmol, 1.00 equiv) was dissolved in 50 mL CH_2Cl_2 and 12.5 mL of NEt_3 (88.4 mmol, 3.00 equiv) were added at 0 °C, followed by drop wise addition of 3.8 mL of 3-chloropivaloyl chloride (29.48 mmol, d = 1.199, 1.00 equiv). After stirring at rt over night the reaction mixture was poured into 10 mL sat. $NaHCO_3$ and the aqueous layer was extracted with Et_2O (3 × 30 mL). The combined organic phases were

dried over $MgSO_4$ and after concentration the remaining oil was distilled in a Kugelrohr oven (170 °C/0.1 Torr) yielding 7.13 g (28.3 mmol, 96% yield) of colorless oily product, which was used in the subsequent step without further purification. An analytically pure sample was obtained by column chromatography on silica gel with EtOAc (R_f 0.45) .

^{1}H-NMR (400.1 MHz, $CDCl_3$, 300K): δ(ppm) 6.51 (d, 1H, J = 8.3 Hz, N*H*), 4.61 (d, 1H, J = 8.6 Hz, NC*H*), 4.38 (m, 1H, C*H*O), 3.77 (s, 3H, COOCH_3), 3.71 (d, 1H, J = 10.6 Hz, CH_2Cl), 3.57 (d, 1H, J = 10.6 Hz, CH_2Cl), 1.37 (s, 3H, CH_3), 1.33 (s, 3H, CH_3), 1.23 (d, 3H, J = 6,6 Hz, CH_3). **^{13}C{^{1}H}-NMR** (125.8 MHz, $CDCl_3$, 300K): δ(ppm) 175.4 (*C*=O), 171.5 (*C*OO), 68.0 (*C*HOH), 57.2 (*C*H), 52.7 (COO*C*H_3), 52.6 (*C*H_2Cl), 44.5 (*C*(CH_3)$_2$), 23.8 (C(*C*H_3)$_2$), 23.2 (C(*C*H_3)$_2$), 20.1 (*C*H_3). **MS** (FAB) m/z (%) 254 (33), 253 (13), 252 ($[M+H]^+$, 100), 234 (16), 202 (8), 192 (12), 119 (7), 116 (10), 102 (22), 93 (8), 91 (22) . **IR** ($\tilde{\nu}$ [cm^{-1}]) 3387m, 2974m, 2956m, 2936w, 2875w, 1744s, 1648s, 1523m, 1475w, 1437m, 1391w, 1349w, 1290m, 1208m, 1083w, 1021w, 997w, 853w, 811w, 731w. **$[\alpha]^{20}_D$** -7.0 (*c* 1.01, $CHCl_3$). **Elemental analysis** for $C_{10}H_{18}ClNO_4$ (251.71 g/mol) calcd %: C, 47.72; H, 7.21; N, 5,56; found: C, 47.47; H, 7.12; N, 5.54.

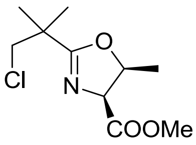

12: (4*S*,5*S*)-Methyl 2-(1-chloro-2-methylpropan-2-yl)-5-methyl-4,5-dihydrooxazole-4-carboxylate

Amide **11 (**1.12 g; 4.45 mmol, 1.00 equiv) and Burgess reagent (1.38 g, 5.79 mmol, 1.30 equiv) were dissolved in 40 mL THF and the mixture refluxed for 4 h. Afterwards THF was removed under reduced pressure, the obtained residue treated with Et_2O and remaining solids were filtered off. The remaining yellowish oil (1.20 g) was distilled in a Kugelrohr

oven (110°C/0.08 Torr) to give 924 mg (3.96 mmol, 89% yield) of the product as colorless oil, which was used in the next step without further purification. When using DAST for the oxazoline-ring closure the product yield was 93%. An analytically pure sample was obtained by column chromatography with EtOAc/Hex (1:5, R_f 0.25).

^{1}H-NMR (500.1 MHz, $CDCl_3$, 300K): δ(ppm) 4.88 (m, 1H, OC*H*), 4.76 (d, 1H, *J* = 10.0 Hz, NC*H*), 3.73 (s, 3H, COOCH_3), 3.63 (m, 2H, CH_2Cl), 1.33 (s, 6H, 2xCH_3), 1.26 (d, 3H, *J* = 6.5 Hz, CH(CH_3)). **^{13}C{^{1}H}-NMR** (125.8 MHz, $CDCl_3$, 300K): δ(ppm) 173.2 (*C*(quart)), 170.3 (*C*OO), 77.8 (O*C*H), 71.3 (N*C*H), 52.2 (*C*H_2Cl), 52.0 (COO*C*H_3), 39.0 (*C*(CH_3)$_2$), 23.6 (C(*C*H_3)$_2$), 23.6 (C(*C*H_3)$_2$), 16.0 (*C*H_3). **MS** (EI, 70 eV): m/z (%): 233 (M^+, 1), 198 (22, $[M-Cl]^+$), 174 (100, $[M-(COOMe)]^+$), 140 (9), 84 (65), 55 (15). **IR** ($\tilde{\nu}$ [cm^{-1}]) 2984m, 2957m, 1736s, 1655s, 1439m, 1386m, 1362w, 1321w, 1290w, 1253w, 1196s, 1174s, 1141w, 1118m, 1044s, 1000w, 973w, 945w, 917w, 892w, 832m, 751w, 634w. **$[\alpha]^{20}_D$** +51,7° (*c* 1.12, $CHCl_3$). **Elemental analysis** for $C_{10}H_{16}ClNO_3$ (233.69 g/mol)) calcd %: C, 51.40; H, 6.90; N, 5.99; found: C, 51.22; H, 6.82; N, 6.11.

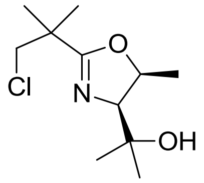

13: 2-((4*S*,5*S*)-2-(1-Chloro-2-methylpropan-2-yl)-5-methyl-4,5-dihydrooxazol-4-yl)propan-2-ol

Oxazoline **12** (2.00 g, 8.58 mmol, 1.00 equiv) was dissolved in 30 mL dry THF and 5.7 mL MeMgCl (3 M in THF, 2.00 equiv) were added drop wise at −78 °C. The reaction mixture was allowed to warm up slowly in the dry ice cooling bath overnight. After quenching with NH_4Cl the mixture was extracted with Et_2O. The organic phases were dried over $MgSO_4$ and

concentrated to give 1.88 g of crude product. Kugelrohr distillation (100°C/0.2 Torr) afforded 1.60 g (6.86 mmol, 80%) of the product **13**.

^{1}H-NMR (500.1 MHz, $CDCl_3$, 300K): δ(ppm) 4.75 (m, 1H, OC*H*), 3.89 (d, 1H, *J* = 9.0 Hz, NC*H*), 3.63 (d, 2H, *J* = 2.5 Hz CH_2Cl), 1.46 (d, 3H, *J* = 7.0 Hz, CH(CH_3), 1.34 (s, 3H, CH_3), 1.31 (s, 3H, CH_3), 1.30 (s, 3H, CH_3), 1.27 (s, 3H, CH_3)). **^{13}C{^{1}H}-NMR** (125.8 MHz, $CDCl_3$, 300K): δ(ppm) 171.0 (*C*(quart.)), 79.4 (O*C*H), 71.8 (N*C*H), 71.8 (*C*OH(quart.)), 52.6 (*C*H_2Cl), 39.0 (*C*($CH_3)_2$), 28.4 (C(*C*$H_3)_2$), 26.2 (C(*C*$H_3)_2$), 23.7 (C(*C*$H_3)_2$), 23.7 (C(*C*$H_3)_2$), 16.1 (*C*H_3). **MS** (FAB) m/z (%) 237 (5), 236 (32), 235 (13), 234 ($[M+H]^+$, 100), 218 (13), 216 (10), 174 (15), 91 (16), 59 (12), 55 (14). **IR** ($\tilde{\nu}$ [cm^{-1}]) 3449m, 2977s, 2939m, 2873m, 2353w, 2343w, 1718m, 1657s, 1468m, 1444m, 1383m, 1364m, 1284m, 1227w, 1180m, 1137m, 1118m, 1076w, 1019m, 948m, 924w, 882w, 825w, 792w, 745w. **$[\alpha]^{20}_D$** +22.6° (*c* 1,15, $CHCl_3$).

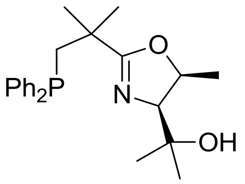

14: 2-((4*S*,5*S*)-2-(1-(Diphenylphosphino)-2-methylpropan-2-yl)-5-methyl-4,5-dihydrooxazol-4-yl)propan-2-ol

Neopentyl chloride **13** (107 mg, 0.46 mmol, 1.00 equiv) was dissolved in 5 mL dry THF and 0.29 mL *n*-BuLi (1.6 M in *n*-hexanes, 0.46 mmol, 1.00 equiv) was added at 0 °C, followed by 0.92 mL of $KPPh_2$ (0.5 M in THF, 0.46 mmol, 1.00 equiv). Then the reaction mixture was warmed to rt and subsequently refluxed for 15 h. The solvent was evaporated and the residue dissolved in 20 mL MTBE. The solution was washed with saturated aq. NH_4Cl (6 mL) and the aqueous layer extracted with MTBE (3 × 10 mL). The combined organic phases were

washed with brine and dried over Na_2SO_4. Chromatography on silica gel with EtOAc/Hex (1:2) afforded 120 mg (0.31 mmol, 68% yield) of the product as a transparent oil which solidified in the refrigerator at +5 °C within a few days.

^{1}H-NMR (500.1 MHz, $CDCl_3$, 300K): δ(ppm) 7.50 (m, 2H, H_{Ar}), 7.41 (m, 2H, H_{Ar}), 7.31 (m, 6H, H_{Ar}), 4.52 (m, 1H, OC*H*), 3.74 (d, 1H, *J* = 9.1 Hz, NC*H*), 2.72 (s, 1H, O*H*), 2.55 (dd, 1H, *J* = 14.3, *J* = 4.9 Hz, CH_2Cl), 2.37 (dd, 1H, *J* = 14.4, *J* = 3.3 Hz, CH_2Cl), 1.49 (d, 3H, *J* = 6.6 Hz, $CH(CH_3)$), 1.32 (s, 3H, CH_3), 1.29 (s, 3H, CH_3), 1.27 (s, 3H, CH_3), 1.24 (s, 3H, CH_3). **^{13}C{^{1}H}-NMR** (125.8 MHz, $CDCl_3$, 300K): δ(ppm) 173.2 (*C*=N), 140.0 (d, *J* = 11.0 Hz, C_{Ar}), 139.0 (d, *J* = 11.0 Hz, C_{Ar}), 133.3 (d, *J* = 20 Hz, C_{ArH}), 132.8 (d, *J* = 20 Hz, C_{ArH}), 128.7 (C_{ArH}), 128.5 (C_{ArH}), 128.4 (d, *J* = 2 Hz, C_{ArH}), 128.3 (d, *J* = 2 Hz, C_{ArH}), 79.2 (O*C*H), 75.6 (N*C*H), 72.2 (*C*OH), 41.0 (d, *J* = 15 Hz, CH_2), 37.2 (d, *J* = 18 Hz, ($C(CH_3)_2$), 29.0 ($HOC(CH_3)_2$), 27.7 (d, *J* = 8 Hz, $C(CH_3)_2$), 27.4 (d, *J* = 10 Hz, $C(CH_3)_2$), 26.15 ($HOC(CH_3)_2$), 16.1 ($CHCH_3$). **^{31}P{^{1}H}-NMR** (202.5 MHz, $CDCl_3$, 300K): δ(ppm) −21.3. **MS** (FAB) m/z (%) 386 (4), 385 (25), 384 ($[M+H]^+$, 100), 326 (2), 325 (13), 324 (43), 306 (11), 285 (17), 284 (32), 228 (6), 227 (38), 202 (10), 201 (18), 199 (11), 185 (27), 183 (13), 136 (7), 91 (6). **$[\alpha]^{20}_D$** +16.1 (*c* 1.00, $CHCl_3$).

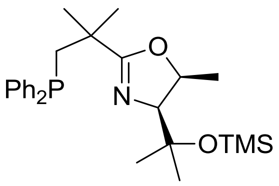

14-TMS: (4*S*,5*S*)-2-(1-(Diphenylphosphino)-2-methylpropan-2-yl)-5-methyl-4-(2-((trimethylsilyl)oxy)propan-2-yl)-4,5-dihydrooxazole

To a solution of alcohol **14** (50.0 mg, 0.13 mmol, 1.00 equiv) in 3 mL dry CH_2Cl_2, 76 μL (0.65 mmol, 5.00 equiv) of 2,6-lutidine were added drop wise at 20 °C. Then 67 μL TMSOTf

(58.0 mg, 0.26 mmol; 2.00 equiv) were added and the solution stirred for 1 h. The solvent was evaporated under high vacuum and the residue was treated with Et_2O. The precipitate was filtered off and the sample dried under high vacuum. Column chromatography on silica gel with EtOAc/Hex/Et_3N (1:10:0,5) gave 40 mg (0.08 mmol, 67% yield) of product **14-TMS** as a colorless oil.

^{1}H-NMR (400.1 MHz, $CDCl_3$, 300K): δ (ppm) 7.47 (m, 4H, H_{Ar}), 7.31 (m, 6H, H_{Ar}), 4.28 (m, 1H, OC*H*), 3.63 (d, *J* = 9.1 Hz, 1H, NC*H*), 2.46 (ddd, *J* = 51.0 Hz, *J* = 14.3 Hz, *J* = 3.7 Hz, 2H, CH_2), 1.47 (d, *J* = 6.8 Hz, 3H, CH(CH_3)), 1.34 (s, 3H, CH_3), 1.32 (s, 3H, CH_3), 1.29 (s, 3H, CH_3), 1.23 (s, 3H, CH_3), 0.11 (s, 9H, Si(CH_3)$_3$). **^{31}P{^{1}H}-NMR** (162.0 MHz, $CDCl_3$, 300K): δ (ppm) −22.3.

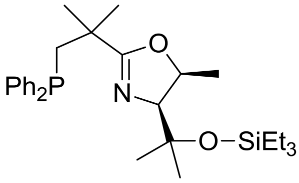

14-TES: (4*S*,5*S*)-2-(1-(Diphenylphosphino)-2-methylpropan-2-yl)-5-methyl-4-(2-((triethylsilyl)oxy)propan-2-yl)-4,5-dihydrooxazole

To a solution of alcohol **14** (50.0 mg, 0.13 mmol, 1.00 equiv) in 3 mL dry CH_2Cl_2, 76 µL (0.65 mmol, 5.00 equiv) of 2,6-lutidine were added drop wise at 20 °C. Then 59 µL TESOTf (69.0 mg, 0.26 mmol, 2.0 equiv) were added and the solution stirred for 3 h. The solvent was evaporated under high vacuum and the residue treated with Et_2O. The precipitate was filtered off and the sample dried under vacuum. Column chromatography on silica gel with EtOAc/Hex (1:9) afforded 47 mg (0.09 mmol, 73% yield) of the product **14-TES** as a colorless oil.

^{1}H-NMR (400.1 MHz, $CDCl_3$, 300K): δ(ppm) 7.46 (q, J = 7.4 Hz, 4H, H_{Ar}), 7.30 (m, 6H, H_{Ar}), 4.32 (m, 1H, OCH), 3.63 (m, 1H, NCH), 2.46 (ddd, J = 60.7 Hz, J = 14.3 Hz, J = 3.8 Hz, 2H, CH_2), 1.46 (d, J = 6.9 Hz, 3H, CH_3), 1.34 (s, 3H, CH_3), 1.31 (s, 3H, CH_3), 1.28 (s, 3H, CH_3), 1.23 (s, 3H, CH_3), 0.95 (t, J = 7.9 Hz, 9H, Si(CH_2CH_3)$_3$), 0.60 (q, J = 8.2 Hz, 6H, Si(C$H_2$$CH_3$)$_3$). **^{13}C{^{1}H}-NMR** (100 MHz, $CDCl_3$, 300K): δ(ppm) 172.8 (C=N), 140.1 (d, J = 12.7 Hz, C_{Ar}), 139.9 (d, J = 13.0 Hz, C_{Ar}), 133.3 (d, J = 19.8 Hz, HC_{Ar}), 133.0 (d, J = 19.5 Hz, HC_{Ar}), 128.5 (C_{ArH}), 128.4 (C_{ArH}), 128.4 (C_{ArH}), 128.4 (C_{ArH}), 79.9 (OCH), 76.1 (NCH), 75.7 (OC(CH_3)$_2$), 41.1 (d, J = 16.6 Hz, $C$$H_2$), 36.9 (d, J = 17.3 Hz, (C(CH_3)$_2$), 30.7 (HOC($C$$H_3$)$_2$), 27.5 (d, J = 9.4 Hz, C($C$$H_3$)$_2$), 27.1 (d, J = 10.5 Hz, C($C$$H_3$)$_2$), 26.4 (HOC($C$$H_3$)$_2$), 16.3 (CH$C$$H_3$), 7.2 ($C$$H_3$, ethyl), 7.0 ($C$$H_2$, ethyl). **^{31}P{^{1}H}-NMR** (202.5 MHz, $CDCl_3$, 300K): δ(ppm) −22.3. **IR** ($\tilde{\nu}$ [cm^{-1}]) 3054w, 2956w, 2911w, 2875w, 1655w, 1433w, 1382w, 1236w, 1163m, 1037m, 857w, 738s, 694s, 506w. **$[\alpha]^{20}_D$** +8.1 (c 1.06, $CHCl_3$).

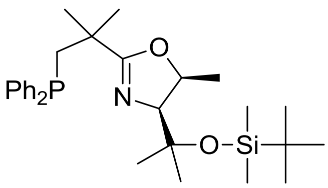

14-TBDMS: (4*S*,5*S*)-4-(2-((*tert*-Butyldimethylsilyl)oxy)propan-2-yl)-2-(1-(diphenylphosphino)-2-methylpropan-2-yl)-5-methyl-4,5-dihydrooxazole

To a solution of alcohol **14** (50.0 mg, 0.13 mmol, 1.00 equiv) in 3 mL dry CH_2Cl_2, 76 µL (0.65 mmol, 5.00 equiv) of 2,6-lutidine were added drop wise at 20 °C. Then 60 µL TBDMSOTf (69.0 mg, 0.26 mmol, 2.00 equiv) were added and the solution stirred for 1 h. Then the solvent was evaporated under high vacuum and the residue treated with Et_2O. The precipitate was filtered off and the solution concentrated. Column chromatography on silica

gel with EtOAc/Hex (1:9, R_f 0.25) afforded 44 mg (0.09 mmol, 68% yield) of the product **14-TBDMS** as a colorless oil.

^{1}H-NMR (500.1 MHz, $CDCl_3$, 300K): δ(ppm) 7.56 – 7.40 (m, 4H, H_{Ar}), 7.38 – 7.23 (m, 6H, H_{Ar}), 4.43 – 4.29 (m, 1H, OC*H*), 3.62 (d, *J* = 9.3 Hz, 1H, NC*H*), 2.47 (ddd, *J* = 55.6 Hz, *J* = 14.4 Hz, *J* = 3.7 Hz, 2H, CH_2P), 1.46 (d, *J* = 6.9 Hz, 3H, CHCH_3), 1.34 (s, 3H, $(CH_3)_2$CO), 1.32 (s, 3H, $(CH_3)_2$C), 1.28 (s, 3H, $(CH_3)_2$C), 1.28 (s, 3H, $(CH_3)_2$CO), 0.86 (s, 9H, $(CH_3)_2$*t*BuSiO), 0.11 (s, 3H, $(CH_3)_2$*t*BuSiO), 0.10 (s, 3H, $(CH_3)_2$*t*BuSiO). **^{13}C{^{1}H}-NMR** (125.8 MHz, $CDCl_3$, 300K): δ (ppm) 172.6 (*C*=N), 140.1 (d, *J* = 13 Hz, C_{Ar}), 139.89 (d, *J* = 13 Hz, C_{Ar}), 133.3 (d, *J* = 20 Hz, C_{ArHo}), 133.0 (d, *J* = 20 Hz, C_{ArHo}), 128.6 – 128.2 (m, $C_{ArHm,p}$), 79.7 (O*C*H), 76.2 (N*C*H), 76.0 (O*C*$(CH_3)_2$), 41.1 (d, *J* = 17 Hz, *C*H_2P), 36.8 (d, *J* = 17 Hz, *C*$(CH_3)_2$), 30,7 (OC($C$$H_3$)$_2$), 27.5 (d, *J* = 10 Hz, C($C$$H_3$)$_2$), 27.1 (d, *J* = 11 Hz, C($C$$H_3$)$_2$), 26.3 (OC($C$$H_3$)$_2$), 26.1 (C($C$$H_3$)$_3$), 18.3 (*C*$(CH_3)_3$), 16.5 (CH*C*$H_3$), -1.7 (Si($C$$H_3$)$_2$), -1.7 (Si($C$$H_3$)$_2$). **^{31}P{^{1}H}-NMR** (202.5 MHz, $CDCl_3$, 300K): δ(ppm) –22.9. **IR** ($\tilde{\nu}$ [cm^{-1}]) 3054w, 2956w, 2929w, 2856w, 1656w, 1434w, 1383w, 1252m, 1162s, 1029s, 834s, 772s, 740s, 694s, 507w. **$[\alpha]^{20}_{D}$** +6.8 (*c* 0.99, $CHCl_3$).

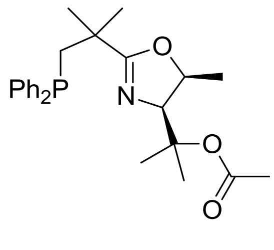

14-OAc: 2-((4*S*,5*S*)-2-(1-(Diphenylphosphino)-2-methylpropan-2-yl)-5-methyl-4,5-dihydrooxazol-4-yl)propan-2-yl acetate

To a solution of ligand **14** (50.0 mg, 0.13 mmol, 1.00 equiv) in 3 mL dry CH_2Cl_2, 76 µL (70 mg, 0.65 mmol, 5.00 equiv) of 2,6-lutidine were added drop wise at 20 °C followed by

20 μL (0.29 mmol, 2.20 equiv) of AcCl. After stirring overnight (16 h) at rt, the reaction mixture was concentrated and subjected to column chromatography on silica gel (EtOAc/Hex 1:4) to afford 42 mg (0.10 mmol, 76% yield) of **14-OAc** as a colorless oil.

^{1}H-NMR (400 MHz, $CDCl_3$, 295K): δ (ppm) 7.45 (m, 4H, H_{Ar}), 7.31 (m, 6H, H_{Ar}), 4.30 (m, 1H, OC*H*), 4.08 (d, *J* = 9.1 Hz, 1H, NC*H*), 2.45 (ddd, *J* = 52.9 Hz, *J* = 14.3 Hz, *J* = 3.8 Hz, 2H, CH_2), 1.96 (s, 3H, CH_3), 1.60 (s, 3H, CH_3), 1.45 (s, 3H, CH_3), 1.40 (d, *J* = 6.8 Hz, 3H, $CHCH_3$), 1.32 (s, 3H, CH_3), 1.28 (s, 3H, CH_3). **^{31}P{^{1}H}-NMR** (162 MHz, $CDCl_3$, 295K): δ (ppm) 22.08. **MS** (EI, 70 eV): m/z (%): 425 (M^+, 1), 366 (13), 324 (31), 284 (95), 227 (100), 183 (30), 121 (20), 91 (5).

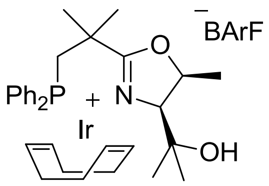

Ir-14-OH

Ligand **14** (30.0 mg, 78.0 μmol, 1.00 equiv) and bis(1,5-cyclooctadiene)diiridium(I) dichloride (26.0 mg, 39.0 μmol, 0.50 equiv) were dissolved in 3 mL dry CH_2Cl_2 and refluxed for 2.5 h under argon. Then of $NaBAr_F$ (90.0 mg, 101 mmol, 1.30 equiv) were added and the mixture stirred for 30 min at rt. Silica gel (2 g) was added and the solvent removed on a rotavap. The immobilized complex was transferred to a silica gel column (2 × 12 cm). The column was flushed with 100 mL of Et_2O followed by 100 mL of CH_2Cl_2. The orange product, eluted by CH_2Cl_2, was collected in one fraction. The resulting orange solution was concentrated under vacuum and the resulting solid dissolved in $CHCl_3$ and concentrated again to remove residual water. The addition/evaporation of $CHCl_3$ was repeated three times. After

drying the residue under high vacuum, 119 mg (78.0 μmol, 99% yield) of complex **Ir-14-OH** was obtained as an orange solid.

^{1}H-NMR (500.1 MHz, $CDCl_3$, 300K): δ(ppm) 7.82 (dd, J = 11.0 Hz, J = 7.3 Hz, 2H, H_{Ar}), 7.72 (s, 8H, $H_{ArF\text{-}o}$), 7.62-7,54 (m, 3H, H_{Ar}), 7.53 (s, 4H, $H_{ArF\text{-}p}$), 7.39 (m, 3H, H_{Ar}), 7.05-7.01 (m, 2H, H_{Ar}), 5.27 (m, 1H, COD-C*H*), 4.84 (m, 1H, COD-C*H*), 4.82 (m, 1H, OC*H*), 3.78 (d, J = 8.8 Hz, 1H, NC*H*), 3.50 (dd, J = 7.1 Hz, J = 3.3 Hz, 1H, COD-C*H*), 2.61 (m, 3H, COD-H, COD-CH_2), 2.55 (d, J = 10 Hz, 2H, CH_2P), 2.32 (m, 2H, COD-CH_2), 2.21 (s, 3H, $C(CH_3)_2$), 2.13 (m, 1H, COD-CH_2), 2.01 (s, 1H, O*H*), 1.89 (m, 1H, COD-CH_2), 1.64 (m, 1H, COD-CH_2), 1.57 (d, J = 6.9 Hz, 3H, $CHCH_3$), 1.49 (d, J = 2.5 Hz, 3H, $C(CH_3)_2$), 1.43 (m, 1H, COD-CH_2), 1.10 (s, 3H, $OC(CH_3)_2$), 0.56 (s, 3H, $OC(CH_3)_2$). **^{13}C{^{1}H}-NMR** (125.8 MHz, $CDCl_3$, 300K): δ (ppm) 180.3 (*C*=N), 161.7 (q, J = 50 Hz, C_{ArFi}), 134.9 (C_{ArH}), 134.8 ($HC_{ArF\text{-}o}$), 132.8 (C_{ArH}), 132.5 (d, J = 55 Hz, C_{Ar}), 131.3 (C_{ArH}), 131.2 (d, J = 10 Hz, C_{ArH}), 130.0 (d, J = 11 Hz, C_{ArH}), 129.1 (qq, J = 3 Hz, J = 32 Hz, $HC_{ArF\text{-}m}$), 129.1 (C_{ArH}), 128.3 (d, J = 54 Hz, C_{Ar}), 124.6 (q, J = 273 Hz, CF_3) 117.5 ($HC_{ArF\text{-}p}$), 96.1 (d, J = 12 Hz, COD-*C*H), 94.2 (d, J = 12 Hz, COD-*C*H), 82.8 (O*C*H), 75.00 (N*C*H), 70.7 ($OC(CH_3)_2$), 63.6 (COD-*C*H), 60.8 (COD-*C*H), 38.9 ($C(CH_3)_2$), 36.3 (COD-CH_2), 33.9 (d, J = 6 Hz, $C(CH_3)_2$), 33.5 (d, J = 32 Hz, CH_2P), 32.0 (COD-CH_2), 28.5 (COD-CH_2), 26.9 (d, J = 12 Hz, $C(CH_3)_2$), 26.4 ($OC(CH_3)_2$), 26.1 (COD-CH_2), 24.5 ($OC(CH_3)_2$), 14.8 ($CHCH_3$). **^{31}P{^{1}H}-NMR** (202.5 MHz, $CDCl_3$, 300K): δ(ppm) 9.5. **^{19}F{^{1}H}-NMR** (376.5 MHz, $CDCl_3$, 300K): δ(ppm) –62.6. **MS** (MALDI-TOF) m/z (%): 684 ($[M-(BAr_F)]^+$, 100).

IR ($\tilde{\nu}$ [cm^{-1}]) 2971w, 1610w, 1439w, 1353m, 1271s, 1110s, 1000w, 886m, 838m, 744w, 711m, 681m, 667m. **$[\alpha]^{20}_D$** +1.3 (*c* 0.70, $CHCl_3$).

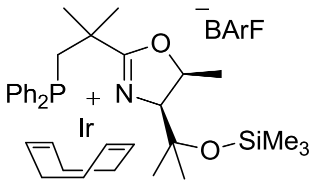

Ir-14-TMS

Following the general procedure for the preparation of **Ir-14-OH**, using 15.0 mg of ligand **14-TMS** (33.0 μmol, 1.00 equiv), 11.0 mg of $[Ir(cod)Cl]_2$ (16.0 μmol, 0.50 equiv) in 3 mL dry CH_2Cl_2 and 37.0 mg of $NaBAr_F$ (42.0 μmol, 1.30 equiv), 43.0 mg (22.4 μmol, 80% yield) of **Ir-14-TMS** were obtained as an orange solid. Crystallization of the product **Ir-14-TMS** from $CHCl_3$ overlaid by *n*-heptane afforded crystals suitable for X-ray crystal analysis.

^{1}H-NMR (500.1 MHz, $CDCl_3$, 300K): δ(ppm) 7.75 (m, 10H, H_{Ar}), 7.58 (m, 7H, H_{Ar}), 7.37 (m, 3H, H_{Ar}), 7.01 (m, 2H, H_{Ar}), 4.80 (m, 3H, CH_2, OC*H*), 3.56 (m, 2H, NC*H*, COD-C*H*), 2.55 (m, 5H, COD), 2.28 (br s, 2H, COD-CH_2, COD-C*H*), 2.17 (s, 3H, C(CH_3)$_2$), 2.08 (m, 1H, COD-CH_2), 1.85 (m, 1H, COD-CH_2), 1.70 (d, *J* = 7.1 Hz, 3H, CHCH_3), 1.61 (m, 1H, COD-CH_2), 1.51 (d, *J* = 2.8 Hz, 3H, C(CH_3)$_2$), 1.39 (s, 3H, OC(CH_3)$_2$), 1.30 (m, 1H, COD-CH_2), 0.85 (s, 3H, OC(CH_3)$_2$), –0.10 (s, 9H, Si(CH_3)$_3$) **^{13}C{^{1}H}-NMR** (125.8 MHz, $CDCl_3$, 300K): δ(ppm) 180.3 (*C*=N), 161.7 (q, *J* = 50 Hz, C_{ArFi}), 135.0 (d, *J* = 11 Hz, C_{ArH}), 134.8 (H$C_{ArF\text{-}o}$), 132.6 (C_{ArH}), 131.2 (d, *J* = 5 Hz, C_{ArH}), 131.1 (C_{ArH}), 129.6 (d, *J* = 11 Hz, C_{ArH}), 129.1 (C_{ArH}), 128.8 (q, *J* = 32 Hz, H$C_{ArF\text{-m}}$), 124.6 (q, *J* = 273 Hz, *C*F_3), 117.5 (H$C_{ArF\text{-}p}$), 94.2 (d, *J* = 12 Hz, COD-*C*H), 93.1 (d, *J* = 12 Hz, COD-*C*H), 84.2 (O*C*H), 74.4 (SiO*C*(CH_3)$_2$), 74.2 (N*C*H), 63.0 (COD-*C*H), 59.7 (COD-*C*H), 38.8 (*C*(CH_3)$_2$), 36.3 (COD-*C*H_2), 33.3 (C(*C*H_3)$_2$), 32.9 (COD-*C*H_2) 32.2 (d, *J* = 32 Hz, *C*H_2P), 29.7 (SiOC(*C*H_3)$_2$), 28.3 (COD-*C*H_2), 27.1 (C(*C*H_3)$_2$), 26.6 (*C*H_3COSi), 25.8 (COD-*C*H_2), 15.2 (*C*H_3CH), 1.08 (SiMe_3). **^{31}P{^{1}H}-NMR** (202.5 MHz, $CDCl_3$, 300K): δ(ppm) 8.8. **^{19}F{^{1}H}-NMR** (376.5 MHz, $CDCl_3$, 300K):

δ(ppm) −62.6. **MS** (MALDI-TOF) m/z (%): 756 ([M−(BAr$_F$)]$^+$, 100). **IR** ($\tilde{\nu}$ [cm^{-1}]) 2971w, 2903w, 1610w, 1588w, 1438w, 1351m, 1272s, 1159m, 1117s, 1047w, 1028w, 989m, 886m, 837m, 744w, 715m, 681m, 667s. **[α]^{20}$_D$** −17.0 (*c* 0.69, $CHCl_3$). **Elemental analysis** for $C_{66}H_{62}NO_2BF_{24}SiPIr$ (1619.27 g/mol) calcd %: C, 48.96; H, 3.86; N, 0.87; found: C, 48.69; H, 3.92; N, 1.12.

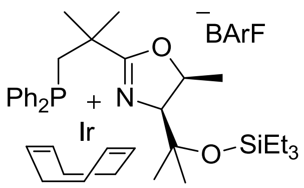

Ir-14-TES

Following the general procedure for the preparation **Ir-14-OH**, starting from 20.0 mg of ligand **14-TES** (40.0 μmol, 1.00 equiv), 11.0 mg of $[Ir(cod)Cl]_2$ (20.0 μmol, 0.50 equiv) in 3 mL dry CH_2Cl_2 and 37.0 mg of NaBAr$_F$ (52.0 μmol, 1.30 equiv), 65.0 mg (40.0 μmol, 99% yield) of **Ir-14-TES** were obtained as an orange solid. Crystallization of the product **Ir-14-TES** from $CHCl_3$ overlaid by *n*-heptane afforded crystals suitable for X-ray crystal analysis.

^{1}H-NMR (500.1 MHz, $CDCl_3$, 300K): δ(ppm) 7.76 (dd, *J* = 10.9 Hz, *J* = 7.7 Hz, 2H, H_{Ar}), 7.72 (s, 8H, $H_{ArF\text{-}o}$), 7.60 (m, 1H, H_{Ar}), 7.54 (m, 2H, H_{Ar}), 7.52 (s, 4H, $H_{ArF\text{-}p}$), 7.39 (m, 3H, H_{Ar}), 7.01 (m, 2H, H_{Ar}), 4.84 (m, 1H, OC*H*), 4.76 (br s, 2H, 2 x COD-C*H*), 3.57 (m, 2H, NC*H*, COD-C*H*), 2.58 (m, 4H, 2 x COD-CH_2), 2.47 (m, 1H, COD-C*H*), 2.28 (m, 2H, CH_2P), 2.18 (s, 3H, C(CH_3)$_2$), 2.08 (m, 1H, COD-CH_2), 1.85 (m, 1H, COD-CH_2), 1.73 (d, *J* = 6.9 Hz, 3H, CHCH_3), 1.62 (m, 1H, COD-CH_2), 1.51 (d, *J* = 2.5 Hz, 3H, C(CH_3)$_2$), 1.41 (s, 3H, OC(CH_3)$_2$), 1.36 (m, 1H, COD-CH_2), 0.80 (m, 12H, SiCH$_2$CH_3, OC(CH_3)$_2$), 0.40 (m, 6H, SiCH_2CH$_3$). **^{13}C{^{1}H}-NMR** (125.8 MHz, $CDCl_3$, 300K): δ (ppm) 180.5 (*C*=N), 161.9 (q, *J* = 50 Hz, C_{ArFi}), 134.9 (d, *J* = 11 Hz, C_{ArH}), 134.8 (H$C_{ArF\text{-}o}$), 132.8 (C_{ArH}), 131.2 (d,

J = 5 Hz, C_{ArH}), 131.1 (C_{ArH}), 129.6 (d, J = 11 Hz, C_{ArH}), 129.0 (C_{ArH}), 128.8 (q, J = 32 Hz, $C_{ArF\text{-}m}$), 124.6 (q, J = 273 Hz, CF_3), 117.5 (H$C_{ArF\text{-}p}$), 94.4 (d, J = 12 Hz, COD-CH), 93.1 (d, J = 12 Hz, COD-*C*H), 84.5 (O*C*H), 74.2 (O*C*$(CH_3)_2$), 74.0 (N*C*H), 63.0 (COD-*C*H), 59.9 (COD-*C*H), 38.9 (*C*$(CH_3)_2$), 36.4 (COD-*C*H_2), 33.4 (d, J = 5 Hz, C(*C*$H_3)_2$), 32.5 (d, J = 34 Hz, *C*H_2P), 30.0 (*C*H_3COSi), 28.4 (COD-*C*H_2), 27.1 (d, J = 12 Hz, *C*$(CH_3)_2$), 26.1 (*C*H_3COSi), 25.9 (COD-*C*H_2), 15.2 (*C*H_3CH), 6.9 (*C*H_3 ethyl), 6.5 (*C*H_2 ethyl). **^{31}P{^{1}H}-NMR** (202.5 MHz, $CDCl_3$, 300K): δ(ppm) 9.0. **^{19}F{^{1}H}-NMR** (376.5 MHz, $CDCl_3$, 300K): δ(ppm) −62.6. **^{11}B-NMR** (160.5 MHz, $CDCl_3$, 300K): δ(ppm) −6.6. **MS** (MALDI-TOF) m/z (%): 798 ($[M-(BAr_F)]^+$, 100). **IR** ($\tilde{\nu}$ [cm^{-1}]) 2959w, 2923w, 2883w, 2855w, 1611w, 1574w, 1460w, 1439w, 1352s, 1273s, 1215w, 1158s, 1115s, 1047w, 1027w, 890m, 837m, 803w, 715m, 680m. **$[\alpha]^{20}_D$** −19.1 (*c* 0.73, $CHCl_3$).

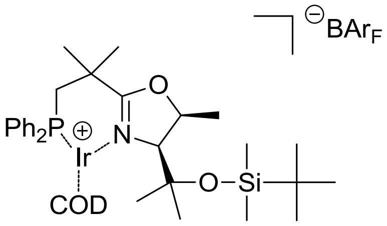

Ir-14-TBDMS

Following the general procedure for the preparation of **Ir-14-OH**, starting from 44.0 mg (88 μmol, 1.00 equiv) of **14-TBDMS**, 30.0 mg (44.0 μmol, 0.50 equiv) of bis(1,5-cyclooctadiene)diiridium(I) dichloride and 102 mg (115 μmol, 1.30 equiv) of $NaBAr_F$, 109 mg (66.0 μmol, 75% yield) of product **Ir-14-TBDMS** were obtained as a yellow solid.

^{1}H-NMR (500.1 MHz, $CDCl_3$, 300K): δ (ppm) 7.81 – 7.75 (m, 10H, 2 x H_{Ar}, 8 x $H_{ArF\text{-}o}$), 7.60 – 7.58 (m, 1H, H_{Ar}), 7.55 – 7.52 (m, 6H, 2 x H_{Ar}, 4 x $H_{ArF\text{-}p}$), 7.40 – 7.36 (m, 3H, H_{Ar}), 7.06 – 7.02 (m, 2H, H_{Ar}), 4.88 (m 1H, OC*H*), 4.78 (br s, 2H, 2 x COD-C*H*), 3.60-3.59 (m, 1H,

COD-C*H*), 3.56 (d, *J* = 8.3 Hz, 1H, NC*H*), 2.70 – 2.53 (m, 4H, CH_2P, COD-CH_2), 2.51 – 2.45 (m, 1H, COD-C*H*), 2.31-2.29 (m, 2H, COD-CH_2), 2.22 (s, 3H, C(CH_3)$_2$), 2.12 – 2.07 (m, 1H, COD-CH_2), 1.90-1.83 (m, 1H, COD-CH_2), 1.79 (d, *J* = 7.0 Hz, 3H, CHCH_3), 1.66-1.61 (m, 1H, COD-CH_2), 1.55 (d, *J* = 2.9 Hz, 3H, C(CH_3)$_2$), 1.42 (s, 3H, OC(CH_3)$_2$), 1.40 – 1.34 (m, 1H, COD-CH_2), 0.95 (s, 3H, OC(CH_3)$_2$), 0.74 (s, 9H, SiC(CH_3)$_3$), 0.05 (s, 3H, SiC(CH_3)$_2$), -0.28 (s, 1H, SiC(CH_3)$_2$). ^{13}**C{^{1}H}-NMR** (125.8 MHz, $CDCl_3$, 300K): δ (ppm) 180.6 (*C*=N), 161.7 (q, *J* = 50 Hz, C_{ArFi}), 135.2 (d, *J* = 12 Hz, C_{ArH}), 134.8 (H$C_{ArF\text{-}o}$), 132.6 (d, *J* = 2 Hz, C_{ArH}), 132.2 (d, *J* = 55 Hz, C_{Ar}), 131.2 (C_{ArH}), 131.1 (C_{ArH}), 129.6 (d, *J* = 11 Hz, C_{ArH}), 129.0 (d, *J* = 10 Hz, C_{ArH}), 128.9 (q, *J* = 32 Hz, H$C_{ArF\text{-}m}$), 128.8 (d, *J* = 54 Hz, C_{ArH}), 124.6 (q, *J* = 273 Hz, CF_3), 117.5 (H$C_{ArF\text{-}p}$), 94.2 (d, *J* = 10 Hz, COD-*C*H), 93.0 (d, *J* = 13 Hz, COD-*C*H), 84.5 (O*C*H), 74.4 (N*C*H), 74.4 (O*C*(CH_3)$_2$), 62.8 (COD-*C*H), 59.7 (COD-*C*H), 38.9 (d, *J* = 2 Hz, (*C*(CH_3)$_2$), 36.4 (d, *J* = 5 Hz, COD-CH_2), 33.7 (d, *J* = 7 Hz, C(CH_3)$_2$), 33.0 (d, *J* = 32 Hz, CH_2P), 32.2 (COD-CH_2), 28.3 (COD-CH_2), 29.7 (OC(CH_3)$_2$), 26.8 (d, *J* = 12 Hz, C(CH_3)$_2$), 25.7 (COD-CH_2), 25.7 (SiC(CH_3)$_3$), 26.1 (OC(CH_3)$_2$), 17.8 (Si*C*(CH_3)$_3$), 15.0 (CHCH_3), -2.0 (SiC(CH_3)$_2$), -2.4 (SiC(CH_3)$_2$). ^{31}**P{^{1}H}-NMR** (202.5 MHz, $CDCl_3$, 300K): δ (ppm) 8.6. ^{11}**B-NMR** (160.5 MHz, $CDCl_3$, 300K): δ (ppm) –6.6. **MS** (MALDI-TOF) m/z (%): 798 ([M−(BAr_F)]$^+$, 100). **IR** ($\tilde{\nu}$ [cm^{-1}]) 2952w, 2859w, 1610w, 1582w, 1471w, 1442w, 1353m, 1273s, 1158m, 1115s, 1047w, 1019w, 1001w, 966w, 886m, 837m, 778w, 744w, 734w, 712m, 682m, 671m. **$[\alpha]^{20}_D$** –21.8 (*c* 0.86, $CHCl_3$). **Elemental analysis** for $C_{69}H_{68}NO_2BF_{24}SiPIr$ (1661.35 g/mol) calcd %: C, 49.88; H, 4.13; N, 0.84; found: C, 49.59; H, 4.19; N, 0.91.

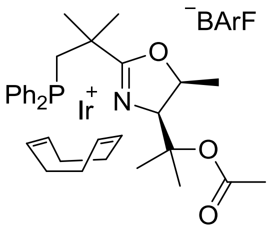

Ir-14-OAc

Following the general procedure for the preparation of **Ir-14-OH**, starting from 42.0 mg (99.0 μmol, 1.00 equiv) of ligand **14-OAc**, 67.0 mg (44.5 μmol, 0.50 equiv) of bis(1,5-cyclooctadiene)diiridium(I) dichloride and 113 mg (128 μmol, 1.30 equiv) of $NaBAr_F$, 128 mg (81.2 μmol, 82% yield) of product **Ir-14-OAc** were obtained as an orange solid.

^{1}H-NMR (500.1 MHz, $CDCl_3$, 300K): δ(ppm) 7.76 (dd, J = 11.0 Hz, J = 7.6 Hz, 2H, H_{Ar}), 7.72 (s, 8H, $H_{ArF\text{-}o}$), 7.60 (m, 1H, H_{Ar}), 7.53 (m, 6H, 2 x H_{Ar}, 4 x $H_{ArF\text{-}p}$), 7.38 (m, 3H, H_{Ar}), 7.02 (m, 2H, H_{Ar}), 4.94 (m, 1H, COD-C*H*), 4.87 (m, 1H, OC*H*), 4.81 (br s, 1H, COD-C*H*), 4.56 (d, J = 8.2 Hz, 1H, NC*H*), 3.62 (br s, 1H, COD-C*H*), 2.57 (m, 5H, CH_2P, COD-CH_2, COD-C*H*), 2.31 (m, 2H, COD-CH_2), 2.20 (s, 3H, $C(CH_3)_2$), 2.12 (m, 1H, COD-CH_2), 1.93 (s, 3H, $COCH_3$), 1.88 (m, 1H, COD-CH_2), 1.65 (s, 3H, $OC(CH_3)_2$, 1H, COD-CH_2), 1.61 (d, J = 7.3 Hz, 3H, $CHCH_3$), 1.49 (s, 3H, $C(CH_3)_2$), 1.40 (m, 1H, COD-CH_2), 0.82 (s, 3H, $OC(CH_3)_2$). **^{13}C{^{1}H}-NMR** (125.8 MHz, $CDCl_3$, 300K): δ(ppm) 181.0 (*C*=N), 170.1 (*C*=O) 161.7 (q, J = 50 Hz, C_{ArFi}), 134,9 (C_{ArH}), 134.8 ($HC_{ArF\text{-}o}$), 132.8 (d, J = 3 Hz, C_{ArH}), 131.9 (d, J = 55 Hz, C_{Ar}), 131.3 (d, J = 3 Hz, C_{ArH}), 131.2 (C_{ArH}), 131.1 (C_{ArH}), 129.7 (d, J = 11 Hz, C_{ArH}), 129.2 (d, J = 11 Hz, C_{ArH}), 128.9 (qq, J = 3 Hz, J = 32 Hz, $HC_{ArF\text{-}m}$), 124.6 (q, J = 273 Hz, CF_3), 117.5 ($HC_{ArF\text{-}p}$), 94.7 (d, J = 11 Hz, COD-*C*H), 93.5 (d, J = 13 Hz, COD-*C*H), 83.9 (O*C*H), 80.9 ($OC(CH_3)_2$), 70.3 (N*C*H), 63.7 (COD-*C*H), 60.6 (COD-*C*H), 39.0 (d, J = 2 Hz, $C(CH_3)_2$), 36.5 (d, J = 5 Hz, COD-CH_2), 33.4 (d, J = 6 Hz, $C(CH_3)_2$), 33.1 (d, J = 32 Hz, CH_2P), 32.3 (COD-CH_2), 28.3 (COD-CH_2), 27.0 (d, J = 12 Hz, $C(CH_3)_2$), 25.8

(COD-CH_2), 25.5 (OC(CH_3)$_2$), 22.1 (C=OCH_3), 21.9 (OC(CH_3)$_2$), 14.7 (CHCH_3). **^{31}P{^{1}H}-NMR** (202.5 MHz, $CDCl_3$, 300K): δ(ppm) 9.1. **^{19}F{^{1}H}-NMR** (376.5 MHz, $CDCl_3$, 300K): δ(ppm) −62.6. **^{11}B-NMR** (160.5 MHz, $CDCl_3$, 300K): δ(ppm) −6.6. **MS** (MALDI-TOF) m/z (%): 726 ([M−(BAr$_F$)]$^+$, 100). **IR** ($\tilde{\nu}$ [cm^{-1}]) 2972w, 1739m, 1609w, 1582w, 1474w, 1437w, 1353s, 1272s, 1114s, 1049w, 1020w, 1000w, 938w, 886m, 838m, 736m, 710m, 681m, 669m. **$[\alpha]^{20}_D$** −15.6 (*c* 1.00, $CHCl_3$). **Elemental analysis** for $C_{65}H_{56}NO_3BF_{24}PIr$ (1589.13 g/mol) calcd %: C, 49.13; H, 3.55; N, 0.88; found: C, 48.86; H, 3.50; N, 0.93.

Serine-derived NeoPHOX ligands

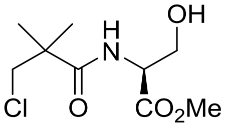

17: (*S*)-Methyl 2-(3-chloro-2,2-dimethylpropanamido)-3-hydroxypropanoate

To a solution of L-serine methylester hydrochloride (1.00 g; 6.43 mmol, 1.00 equiv) in 10 mL of CH_2Cl_2, 2.7 mL (19.3 mmol, 3.00 equiv) of NEt_3 were added at 0 °C. After stirring for 30 min at 0 °C, 0.83 mL (6.43 mmol, 1.00 equiv) of 3-chloropivaloyl chloride was added drop wise and the mixture was stirred for 1 h at rt. The reaction mixture was poured into a saturated solution of $NaHCO_3$ and the aqueous layer was extracted with Et_2O. The combined organic phases were dried over $MgSO_4$ and concentrated. Column chromatography on silica gel using EtOAc as eluent (R_f 0.4) gave 1.21 g (5.08 mmol, 79% yield) of product **17** as a colorless oil.

^{1}H-NMR (500 MHz, $CDCl_3$) δ = 6.85 (d, *J* = 7.1 Hz, 1H, N*H*), 4.64 – 4.57 (m, 1H, NC*H*), 3.92 (ddd, *J* = 43.8 Hz, *J* = 11.3 Hz, *J* = 3.5 Hz, 2H, CH_2O), 3.80 (s, 3H, CO_2Me), 3.61 (dd, *J* = 38.7 Hz, *J* = 10.8 Hz, 2H, CH_2Cl), 1.32 (s, 3H, CH_3), 1.30 (s, 3H, CH_3). **^{13}C{^{1}H}-NMR**

(125.8 MHz, $CDCl_3$, 300K): δ(ppm) 175.3 (*C*=O), 171.0 (*C*OO), 62.8 (*C*H_2OH), 54.9 (*C*H), 52.7 (COO*C*H_3), 52.7 (*C*H_2Cl), 44.3 (*C*(CH_3)$_2$), 23.5 (C(*C*H_3)$_2$), 23.1 (C(*C*H_3)$_2$). **MS** (FAB) m/z (%) 239 (10), 238 ($[M+H]^+$, 100), 220 (19), 178 (14), 102 (70), 91 (28), 55 (9). **IR** ($\tilde{\nu}$ [cm^{-1}]) 3382br w, 2954w, 1739m, 1651s, 1517m, 1252w, 1248w, 1286m, 1209m, 1101w, 1078m. **$[\alpha]^{20}_D$=** +21.0 (*c* 1.10, $CHCl_3$). **Elemental analysis**: calc.: C, 45.48; H, 6.79; N, 5.89; found: C, 45.15; H, 6.72; N, 5.80.

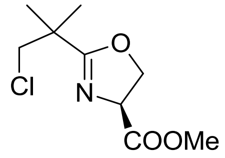

18: (*S*)-Methyl 2-(1-chloro-2-methylpropan-2-yl)-4,5-dihydrooxazole-4-carboxylate

To a solution of 700 mg (2.95 mmol, 1.00 equiv) of amide **17** in 15 mL of dry CH_2Cl_2 at −78 °C, 0.43 mL DAST (3.24 mmol, 1.10 equiv) was added drop wise. After stirring for 1 h at −78 °C, 610 mg (4.42 mmol, 1.50 equiv) of solid anhydrous K_2CO_3 was added and the reaction mixture was allowed to warm to rt. The reaction mixture was poured into a saturated aqueous solution of $NaHCO_3$ and extracted with CH_2Cl_2 (3 × 20 mL). After drying over $MgSO_4$, the solvent was removed to give 660 mg of crude product as an ochre-colored oil. Kugelrohr distillation (110 °C/0.08 Torr) afforded 612 mg (2.80 mmol, 95% yield) of product **18** as a colorless oil.

^{1}H-NMR (500 MHz, $CDCl_3$) δ 4.75 (dd, *J* = 10.5 Hz, *J* = 7.7 Hz, 1H, NC*H*), 4.50 (dd, *J* = 8.7 Hz, *J* = 7.7 Hz, 1H, OC*H*$_2$), 4.41 (dd, *J* = 10.5 Hz, *J* = 8.7 Hz, 1H, OCH_2), 3.77 (s, 3H, CO_2Me), 3.62 (q, *J* = 10.8 Hz, 2H, C*H*$_2$Cl), 1.33 (s, 3H, C*H*$_3$), 1.31 (s, 3H, C*H*$_3$). **^{13}C-NMR** (126 MHz, $CDCl_3$) δ 173.27 (O*C*=N), 171.58 (*C*=O), 69.69 (O*C*H_2), 68.14 (N*C*H), 52.65 (CO_2*C*H_3), 52.28 (*C*H_2Cl), 39.06 (*C*(CH_3)$_2$), 23.73 (*C*H_3), 23.65 (*C*H_3). **MS** (EI, 70 eV): m/z

(%): 219 (M^+, 33), 187 (30), 119 (18), 91 (100), 55 (41). **IR** ($\tilde{\nu}$ [cm^{-1}]) 3406w, 2955w, 1728m, 1649s, 1633m, 1499m, 1472w, 1435m, 1387w, 1364w, 1329w, 1244m, 1171m, 1140w, 962w, 905w, 770m. **$[\alpha]^{20}_D$**= 108.4 (*c* 0.95, $CHCl_3$). **Elemental analysis**: calc.: C, 49.21; H, 6.42; N, 6.38; found: C, 48.27; H, 6.13; N, 6.08.

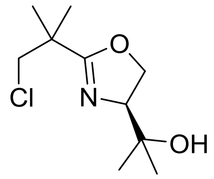

19: (*S*)-2-(2-(1-Chloro-2-methylpropan-2-yl)-4,5-dihydrooxazol-4-yl)propan-2-ol

To a solution of 270 mg (3.19 mmol, 1.00 equiv) of oxazoline **18** in 15 mL dry THF, 2.55 mL of MeMgCl solution (3 M in THF; 7.65 mmol, 2.40 equiv) were added drop wise at 0 °C and the solution was stirred at this temperature for 1.5 h. After quenching with NH_4Cl and extraction with Et_2O (3 × 20 mL), the combined organic phases were dried over Na_2SO_4 and concentrated to give 245 mg of a yellowish oil. Column chromatography on silica gel with EtOAc/MeOH (9:1) afforded 210 mg (2.45 mmol, 78%) of product **19** as a colorless oil.

^{1}H-NMR (400 MHz, $CDCl_3$) δ 4.24 (dd, *J* = 8.9 Hz, *J* = 3.8 Hz, 2H, OCH_2), 4.04 (dd, *J* = 10.0 Hz, *J* = 7.7 Hz, 1H, NC*H*), 3.65-3.59 (m, 2H, CH_2Cl), 1.92 (brs, 1H, O*H*), 1.33 (s, 3H, C(CH_3)$_2$), 1.31 (s, 3H, C(CH_3)$_2$), 1.28 (s, 3H, HOC(CH_3)$_2$), 1.13 (s, 3H, HOC(CH_3)$_2$). **^{13}C-NMR** (100 MHz, $CDCl_3$) δ 171.7(O*C*=N), 75.3 (N*C*H.), 71.5 (O*C*(CH_3)$_2$), 69.1 (OCH_2), 52.9 (*C*H_2Cl), 39.2 (*C*(CH_3)$_2$), 27.0 (C(*C*H_3)$_2$), 24.9 (C(*C*H_3)$_2$), 24.1 (C(*C*H_3)$_2$), 23.9 (C(*C*H_3)$_2$). **MS** (EI, 70 eV): m/z (%): 219 (M^+, 33), 187 (30), 119 (18), 91 (100), 55 (41). **IR** ($\tilde{\nu}$ [cm^{-1}]) 3406w, 2955w, 1728m, 1649s, 1633m, 1499m, 1472w, 1435m, 1387w, 1364w, 1329w, 1244m, 1171m, 1140w, 962w, 905w, 770m. **$[\alpha]^{20}_D$**= +52.2 (*c* 1.04, $CHCl_3$).

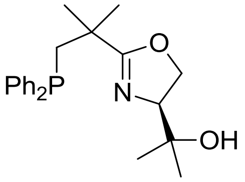

20: (*S*)-2-(2-(1-(Diphenylphosphino)-2-methylpropan-2-yl)-4,5-dihydrooxazol-4-yl)propan-2-ol

A solution of $KPPh_2$ in dry THF (1.1 mL; 0.5 M, 546 µmol, 1.2 equiv) was added to a solution of 100 mg (455 µmol, 1.00 equiv) of **19** in 15 mL of dry THF and the reaction mixture was refluxed overnight. The solvent was evaporated and the residue dissolved in a mixture of 15 mL of Et_2O, 3 mL of sat. aq. NH_4Cl and 2 mL of water. The phases were separated and the aqueous layer extracted with Et_2O (3 × 20 mL). The organic phases were dried over Na_2SO_4 and concentrated. The crude product (210 mg) was purified by column chromatography on silica gel using EtOAc/Hex 1:2 (R_f 0.25) and then EtOAc/Hex 1:1 (R_f 0.35) as eluent to afford 65 mg (177 µmol, 39% yield) of product **20** as a white solid.

^{1}H-NMR (500.1 MHz, $CDCl_3$, 300K): δ(ppm) 7.51–7.58 (m, 2H, H_{Ar}), 7.43–7.40 (m, 2H, H_{Ar}), 7.34–7.28 (m, 6H, H_{Ar}), 4.26-4.24 (m, 1H, OC*H*),), 4.05-4.02 (m, 1H, OC*H*), 3.95-3,93 (m, 1H, NC*H*), 2.63 (br s, 1H, O*H*), 2.57 (dd, 1H, *J* = 14.2 Hz, *J* = 4.9 Hz, CH_2Cl), 2.37 (dd, 1H, *J* = 14.2 Hz, *J* = 3.5 Hz, CH_2Cl), 1.32 (s, 3H, $OC(CH_3)_2$), 1.28 (s, 3H, $C(CH_3)_2$), 1.24 (s, 3H, $C(CH_3)_2$), 1.11 (s, 3H, $OC(CH_3)_2$). **^{13}C{^{1}H}-NMR** (125.8 MHz, $CDCl_3$, 300K): δ(ppm) 174.0 (*C*=N), 139.1 (d, *J* = 10.0 Hz, C_{Ar}), 138.6 (d, *J* = 10.0 Hz, C_{Ar}), 133.1 (d, *J* = 19 Hz, HC_{Ar}), 132.8 (d, *J* = 19 Hz, HC_{Ar}), 128.7 (C_{ArH}), 128.5 (C_{ArH}), 128.5 (C_{ArH}), 128.4 (C_{ArH}), 74.8 (N*C*H), 71.5 (O*C*($CH_3)_2$), 68.7 (OCH_2), 41.2 (d, *J* = 14 Hz, CH_2P), 37.3 (d, *J* = 8 Hz, (*C*($CH_3)_2$), 27.9 (d, *J* = 7 Hz, C($CH_3)_2$), 27.4 (OC($CH_3)_2$), 27.3 (d, *J* = 9 Hz,

C(CH_3)$_2$), 25.0 (OC(CH_3)$_2$). **^{31}P{^{1}H}-NMR** (202.5 MHz, $CDCl_3$, 300K): δ (ppm) 21.4. **IR** ($\tilde{\nu}$ [cm^{-1}]) 3370w, 3060w, 2974w, 2929w, 1641m, 1433m, 1370w, 1185m, 1130m, 969m, 747m, 697s, 505m. **$[\alpha]^{20}_D$**= +29.2 (*c* 1.09, $CHCl_3$).

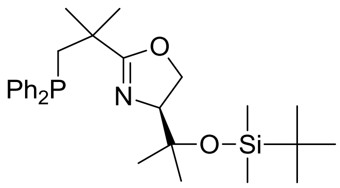

20-TBDMS: (*S*)-4-(2-((*tert*-Butyldimethylsilyl)oxy)propan-2-yl)-2-(1-(diphenylphosphino)-2-methylpropan-2-yl)-4,5-dihydrooxazole

2,6-Lutidine (70 μL; 610 μmol, 5.00 equiv) was added drop wise to a solution of 45.0 mg (122 μmol, 1.00 equiv) of alcohol **20** in 3 mL of dry CH_2Cl_2 at 20 °C. Then 56 μL (*d* = 1.151; 244 mmol, 2.00 equiv) of TBDMSOTf were added and the solution was stirred for 1 h at rt. The solvent was evaporated and the residue dissolved in Et_2O. After evaporation of the solvent and drying in high vacuum, 60 mg of crude product was obtained, which was purified by column chromatography on silica gel with EtOAc/Hex (1:9; R_f 0.25) to afford 32.0 mg (329 μmol, 54% yield) of ligand **20-TBDMS**.

^{1}H-NMR (500.1 MHz, $CDCl_3$, 300K): δ (ppm) 7.48 – 7.44 (m, 4H, H_{Ar}), 7.33 – 7.28 (m, 6H, H_{Ar}), 4.20 – 4.17 (m, 1H, OCH_2), 3.77-3.69 (m, 2H, NC*H*, 1 x OCH_2), 2.52-2.41 (m, 2H, CH_2P), 1.35 (s, 3H, OC(CH_3)$_2$), 1.29 (s, 3H, OC(CH_3)$_2$), 1.24 (s, 3H, C(CH_3)$_2$), 1.13 (s, 3H, C(CH_3)$_2$), 0.82 (s, 9H, $(CH_3)_2$*t*BuOSi), 0.07 (s, 3H, (CH_3)$_2$*t*BuOSi), 0.07 (s, 3H, (CH_3)$_2$*t*BuOSi). **^{13}C{^{1}H}-NMR** (125.8 MHz, $CDCl_3$, 300K): δ (ppm) 173.3 (*C*=N), 139.7 (d,

J = 13 Hz, C_{Ar}), 139.6 (d, J = 13 Hz, C_{Ar}), 133.2 (d, J = 20 Hz, C_{ArHo}), 132.8 (d, J = 20 Hz, C_{ArHo}), 128.4 – 128.3 (m, $C_{ArHm,p}$), 75.7 (OC(CH_3)$_2$), 74.7 (NCH), 68.7 (O$C$$H_2$), 41.1 (d, J = 17 Hz, $C$$H_2$P), 36.7 (d, J = 17 Hz, C(CH_3)$_2$), 28,7 (OC($C$$H_3$)$_2$), 27.5 (d, J = 9 Hz, C($C$$H_3$)$_2$), 27.1 (d, J = 10 Hz, C($C$$H_3$)$_2$), 26.3 (OC($C$$H_3$)$_2$), 25.8 (C($C$$H_3$)$_3$), 18.2 ($C$($CH_3$)$_3$), −2.1 (Si($C$$H_3$)$_2$), −2.2 (Si($C$$H_3$)$_2$). **^{31}P{^{1}H}-NMR** (202.5 MHz, $CDCl_3$, 300K): δ (ppm) −23.4. **MS** (EI, 70 eV): m/z (%): 483 (M^+, 2), 468 (8), 426 (46), 406 (42), 310 (78), 274 (12), 227 (53), 173 (100) 121 (21), 73 (38). **IR** ($\tilde{\nu}$ [cm^{-1}]) 2963m, 2947m, 2927m, 2883m, 2853m, 1733w, 1680m, 1580w, 1470m, 1437m, 1359m, 1303w, 1251m, 1186s, 1160s, 1118s, 1102m, 1051s, 1034s, 1004m, 985m, 897w, 822s, 772s, 738s, 714s, 652s. **$[\alpha]^{20}_D$**= +12.5 (c 1.05, $CHCl_3$).

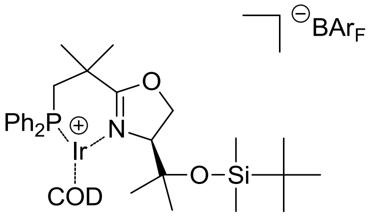

Ir-20-TBDMS:

Following the general procedure for the preparation **Ir-14-OH**, using 30.0 mg (62.0 µmol, 1.00 equiv) of **20-TBDMS**, 21.0 mg (31.0 µmol, 0.50 equiv) of bis(1,5-cyclooctadiene)diiridium(I) dichloride in 3 mL of dry CH_2Cl_2, and 72.0 mg (81.0 µm, 1.30 equiv) of $NaBAr_F$, 79.0 mg (47.7 µmol, 77% yield) of complex **Ir-20-TBDMS** were obtained as a yellow solid.

^{1}H-NMR (500.1 MHz, $CDCl_3$, 300K): δ (ppm) 7.81 – 7.72 (m, 10H, 2 x H_{Ar}, 8 x $H_{ArF\text{-}o}$), 7.60 – 7.55 (m, 1H, H_{Ar}), 7.55 – 7.49 (m, 6H, 2 x H_{Ar}, 4 x $H_{ArF\text{-}p}$), 7.41 – 7.35 (m, 3H, H_{Ar}), 7.08 – 6.96 (m, 2H, H_{Ar}), 4.88 (dd, J = 9.7 Hz, J = 4.0 Hz, 1H, OCH_2), 4.83 (m, 1H, COD-

CH), 4.81 – 4.75 (m, 1H, COD-CH), 4.37 (t, J = 9.7 Hz, 1H, OCH_2), 3.81 (dd, J = 9.8 Hz, J = 4.0 Hz, 1H, NCH), 3.67 – 3.61 (m, 1H, COD-CH), 2.68 – 2.48 (m, 4H, CH_2P, COD-CH_2), 2.48 – 2.42 (m, 1H, COD-CH), 2.28 (m, 2H, COD-CH_2), 2.24 (s, 3H, C(CH_3)$_2$), 2.13 – 2.02 (m, 1H, COD-CH_2), 1.84 (m, 1H, COD-CH_2), 1.61 (m, 1H, COD-CH_2), 1.51 (d, J = 2.9 Hz, 3H, C(CH_3)$_2$), 1.43 – 1.31 (m, 1H, COD-CH_2), 1.38 (s, 3H, OC(CH_3)$_2$) 0.84 (s, 3H, OC(CH_3)$_2$), 0.69 (s, 9H, SiC(CH_3)$_3$), 0.03 (s, 3H, SiC(CH_3)$_2$), –0.26 (s, 3H, SiC(CH_3)$_2$). **^{13}C{^{1}H}-NMR** (125.8 MHz, CDCl$_3$, 300K): δ (ppm) 180.2 (d, J = 2 Hz, C=N), 161.8 (q, J = 50 Hz, C_{ArFi}), 135.1 (d, J = 12 Hz, C_{ArH}), 134.8 (H$C_{ArF\text{-}o}$), 132.8 (d, J = 2 Hz, C_{ArH}), 132.3 (d, J = 55 Hz, C_{Ar}) 131.2 (d, J = 2 Hz, C_{ArH}), 131.1 (d, J = 10 Hz, C_{ArH}) 129.6 (d, J = 11 Hz, C_{ArH}), 129.1 (d, J = 10 Hz, C_{ArH}), 128.9 (q, J = 32 Hz, H$C_{ArF\text{-}m}$), 128.7 (d, J = 54 Hz, C_{ArH}), 124.6 (q, J = 273 Hz, CF$_3$), 117.5 (H$C_{ArF\text{-}p}$), 94.2 (d, J = 10 Hz, COD-CH), 92.1 (d, J = 13 Hz, COD-CH), 73.9 (NCH), 73.5 (OC(CH$_3$)$_2$), 71.6 (OCH$_2$), 63.3 (COD-CH), 60.3 (COD-CH), 38.9 (C(CH$_3$)$_2$), 36.4 (COD-CH$_2$), 33.8 (d, J = 5 Hz, C(CH$_3$)$_2$), 33.2 (d, J = 32 Hz, CH$_2$P), 32.4 (COD-CH$_2$), 28.7 (OC(CH$_3$)$_2$), 28.1 (COD-CH$_2$), 27.0 (d, J = 12 Hz, C(CH$_3$)$_2$), 25.6 (COD-CH$_2$), 25.4 (SiC(CH$_3$)$_3$), 24.2 (OC(CH$_3$)$_2$), 17.8 (SiC(CH$_3$)$_3$), –2.5 (SiC(CH$_3$)$_2$), –2.9 (SiC(CH$_3$)$_2$). **^{31}P{^{1}H}-NMR** (202.5 MHz, CDCl$_3$, 300K): δ(ppm) 9.2. **MS** (MALDI-TOF) m/z (%): 784 ([M–(BAr$_F$)]$^+$, 100). **IR** ($\tilde{\nu}$ [cm^{-1}]) 2963w, 2947w, 1610w, 1580w, 1437w, 1351m, 1271s, 1160m, 1114s, 1103s, 1199s, 1196s, 1001m, 970m, 895w, 886m, 838m, 777m, 743m, 715s, 710s, 668s. **[α]$^{20}_D$**= –17.4 (c 0.70, CHCl$_3$). **Elemental analysis:** calc.: C, 49.88; H, 4.13; N, 0.84; found: C, 49.59; H, 4.19; N, 0.91.

General procedure for allylic substitution

A solution of 1.8 mg of [Pd(allyl)Cl]$_2$ (0.01 mmol) and 0.025 mmol of the appropriate ligand in 1.2 mL of dry CH$_2$Cl$_2$ was degassed in a Young tube by three freeze-pump-thaw cycles and then stirred for 2 h at 50 °C. In a second Young tube, 1.00 mmol of the substrate was

dissolved in 4 mL of dry CH_2Cl_2 and 396 mg of dimethyl malonate (3.00 mmol), BSA (0.73 mL; 610 mg; 3.00 mmol) and 1 mg of dried KOAc were added. The mixture was degassed by three freeze-pump-thaw cycles and then the catalyst solution was added. The resulting reaction mixture was stirred at rt for 24 h under argon. For workup the reaction mixture was diluted with Et_2O and quenched by addition of 20 mL of sat. aq. NH_4Cl solution. The aqueous layer was extracted with Et_2O (3 × 15 mL) and the combined organic extracts were dried over $MgSO_4$. After removal of the solvent and column chromatography on silica gel (hexanes/EtOAc/Et_3N 18:1:1) the product was obtained as a white solid.

Determination of the enantiomeric excess

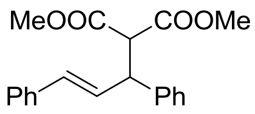

HPLC (Daicel Chiracel AD-H, heptane/isopropanol 97:3, 0.9 mL·min^{-1}, 20 °C, 254 nm); t_R = 16.3 (*R*), 17.9 (*S*) min.

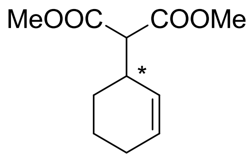

GC (β-cyclodextrin PM, 130 °C, 100 kPa); t_R = 21.7 (*R*), 23.7 (*S*) min.

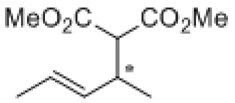

Integration of the OMe peaks in the ^{1}H NMR spectrum using Eu(hfc)$_3$ as chiral shift reagent.

Asymmetric hydrogenation

All hydrogenation reactions were performed according to a published procedure [1] at rt under 50 bar of H_2 gas with 1 mol % of catalyst (substrate concentration 0.2 mol/L) in dichloromethane (Aldrich, crown cap).

Product analyses

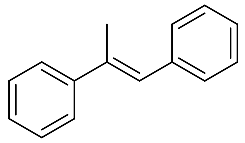

Hydrogenation of *E*-1,2-diphenylpropene:

GC: Restek Rtx-1710 column (30 m × 0.25 mm × 0.25 μm), 60 kPa He, (100 °C – 2 min - 7 K/min – 250 °C -10 min): t_R = 18.2 min (product), 23.8 min (starting material)

HPLC: (Diacel Chiracel OJ (2.6 × 250 mm), heptane/isopropanol 99:1, 0.5 mL/min, 20 °C, 220 nm, t_R = 15.6 min (*R*), 23.8 min (*S*)

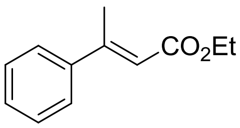

Hydrogenation of ethyl *E*-2-methylcinnamate:

GC: Chiraldex γ-cyclodextrin TFA G-TA (30 m × 0.25 mm × 0.12 μm) 60 kPa H_2, (85 °C – 50 min -10 K / min – 160 °C): t_R = 42.9 min ((*R*)-product), 44.9 min ((*S*)-product), 57.0 min (starting material).

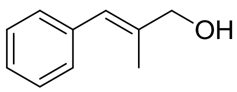

Hydrogenation of *E*-2-methyl-3-phenylprop-2-enol:

GC: Restek Rtx-1710 column (30 m × 0.25 mm × 0.25 μm), 60 kPa He, (100 °C – 2 min - 7 K/min – 250 °C -10 min): t_R = 14.6 min (product), 16.5 min (starting material).

HPLC: (Diacel Chiracel OD-H (2.6 × 250 mm), heptane/isopropanol 95:5, 0.5 mL/min, 40 °C, 200 nm, t_R = 15.3 min (+), 17.5 min (−).

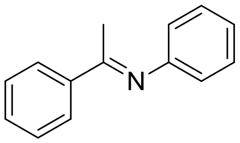

Hydrogenation of *E*-phenyl-(1-phenylethylidene)amine:

GC: Restek Macherey-Nagel Optima 5-Amin (30 m × 0.25 mm × 0.5 μm), 60 kPa He, (150 °C -7 K/min – 250 °C -10 min): t_R = 12.8 min (product), 13.2 min (starting material)

HPLC: (Diacel Chiracel OD-H (2.6 × 250 mm), heptane/isopropanol 99:1, 0.5 mL/min, 20 °C, 210 nm, t_R = 24.6 min (*S*), 33.0 min (*R*).

Crystallographic data

X-ray crystal structures were measured by Dr. Markus Neuburger (Department of Chemistry, University of Basel) on a Bruker Nonius KappaCCD diffractometer using graphite-monochromated MoKα radiation and solved using direct methods (Sir97, [2] Superflip [3] or SHELX [4]) and refined in Crystals [5]. Least-squares refinement against F was carried out on all non-hydrogen atoms. Chebychev polynomial weights were used to complete the refinement [6]. The absolute configuration was be determined by definition of the flack parameter [7]. Data were recorded at 123 K. Crystals were usually grown by dissolving the

complex in dichloromethane or chloroform and carefully overlaying the solution with *n*-heptane. The crystals were mounted with paraffin on a glass fiber goniometer head.

Crystallographic data of the complexes ser-PHOX-OMe (CCDC 1473372), Ir-14-TES (CCDC 1473373), Ir-14-TMS (CCDC 1473374), Ir-14-TBDMS (CCDC 1473375), Ir-20-TBDMS (CCDC 1473376), and Ir-1b (CCDC 1473377) have been deposited at the Cambridge Crystallographic Data Center. These data can be obtained free of charge from The Cambridge Crystallographic Data Centre via www.ccdc.cam.ac.uk/data_req.uest/cif.

Compound	Ir-14-TES	Ir-14-TMS
formula	$C_{70}H_{69}BCl_3F_{24}IrNO_2PSi$	$C_{67}H_{63}BCl_3F_{24}IrNO_2PSi$
formula weight ($g \cdot mol^{-1}$)	1780.72	1738.64
shape	block	block
color	orange	orange
temperature [K]	123	123
crystal size [mm^3]	0.060 · 0.130 · 0.240	0.070 · 0.150 · 0.240
crystal system	orthorhombic	orthorhombic
space group	$P\ 2_1\ 2_1\ 2_1$	$P\ 2_1\ 2_1\ 2_1$
a [Å]	14.8070(10)	15.1113(5)
b [Å]	17.4577(12)	17.7434(7)
c [Å]	28.8726(19)	27.3178(10)
α [°]	90	90
β [°]	90	90
γ [°]	90	90
volume [Å]^3	7463.5(9)	7324.6(5)
Z	4	4
density (calc.) [$g \cdot cm^{-1}$]	1.585	1.577
μ(Mo K_α) [mm^{-1}]	2.038	2.075
transmission (min/max)	0.77 / 0.88	0.73 / 0.86
Θ range for data collection [°]	1.546 - 27.899	1.770 - 29.136
radiation (λ [Å])	0.71073	0.71073
F(000)	3560	3464
measured reflections	68593	113578
independent reflections	17796 (merging r = 0.028)	19676 (merging r = 0.032)
observed reflections	16608 ($I>2.0\sigma(I)$)	16944 ($I>2.0\sigma(I)$)
parameters refined	938	1022
R	0.0239	0.0252
R_w	0.0286	0.0329
goodness of fit on F	1.0808	1.1085
flack parameter	-0.010(2)	-0.008(2)

**Compound**	**Ir-14-TBDMS**	**Ir-20-TBDMS**
formula	$C_{69}H_{68}BF_{24}IrNO_2PSi$	$C_{68}H_{66}BF_{24}IrNO_2PSi$
formula weight ($g\cdot mol^{-1}$)	1661.35	1647.32
shape	block	block
color	orange	orange
temperature [K]	123	123
crystal size [mm^3]	0.060 · 0.170 · 0.220	0.070 · 0.180 · 0.210
crystal system	orthorhombic	orthorhombic
space group	$P\ 2_1\ 2_1\ 2_1$	$P\ 2_1\ 2_1\ 2_1$
a [Å]	13.0330(3)	12.9974(3)
b [Å]	19.5152(4)	19.2190(4)
c [Å]	27.7197(6)	27.4554(5)
α [°]	90	90
β [°]	90	90
γ [°]	90	90
volume [Å]^3	7050.3(3)	6858.3(2)
Z	4	4
density (calc.) [$g\cdot cm^{-1}$]	1.565	1.595
μ(Mo K_α) [mm^{-1}]	2.042	2.098
transmission (min/max)	0.71 / 0.88	0.69 / 0.86
Θ range for data collection [°]	1.727 - 37.789	1.733 - 37.789
radiation (λ [Å])	0.71073	0.71073
F(000)	3328	3296
measured reflections	297104	296707
independent reflections	37854 (merging r = 0.044)	36782 (merging r = 0.042)
observed reflections	30699 ($I>2.0\sigma(I)$)	31942 ($I>2.0\sigma(I)$)
parameters refined	1031	930
R	0.0246	0.0222
R_w	0.0326	0.0265
goodness of fit on F	1.0931	1.0944
flack parameter	-0.0085(19)	-0.0087(15)

References

[1] Woodmansee, D. H.; Müller, M.-A.; Tröndlin, L.; Hörmann, E.; Pfaltz, A. *Chem. Eur. J.* **2012**, *18*, 13780-13786.

[2] Altomare, A.; Caserano, G.; Giacovazzo, C.; Guagliardi, A. *J. Appl. Crystallogr.* **1993**, *26*, 343-350.

[3] Palatinus, L.; Chapuis, G. *J. Appl. Crystallogr.* **2007**, *40*, 786-790.

[4] Sheldrick, G. *Acta Crystallographica Section A* **2008**, *64*, 112-122.

[5] Betteridge, P. W.; Carruthers, J. R.; Cooper, R. I.; Prout, K.; Watkin, D. J. *J. Appl. Crystallogr.* **2003**, *36*, 1487.

[6] Carruther, J. R.; Watkin, D. J. *Acta Crystallographica Section A* **1979**, *35*, 698-699.

[7] Flack, H. D.; Bernardinelli, G. *J. Appl. Crystallogr.* **2000**, *33*, 1143-1148.

^{1}H and ^{13}C NMR spectra

^{1}H NMR 11

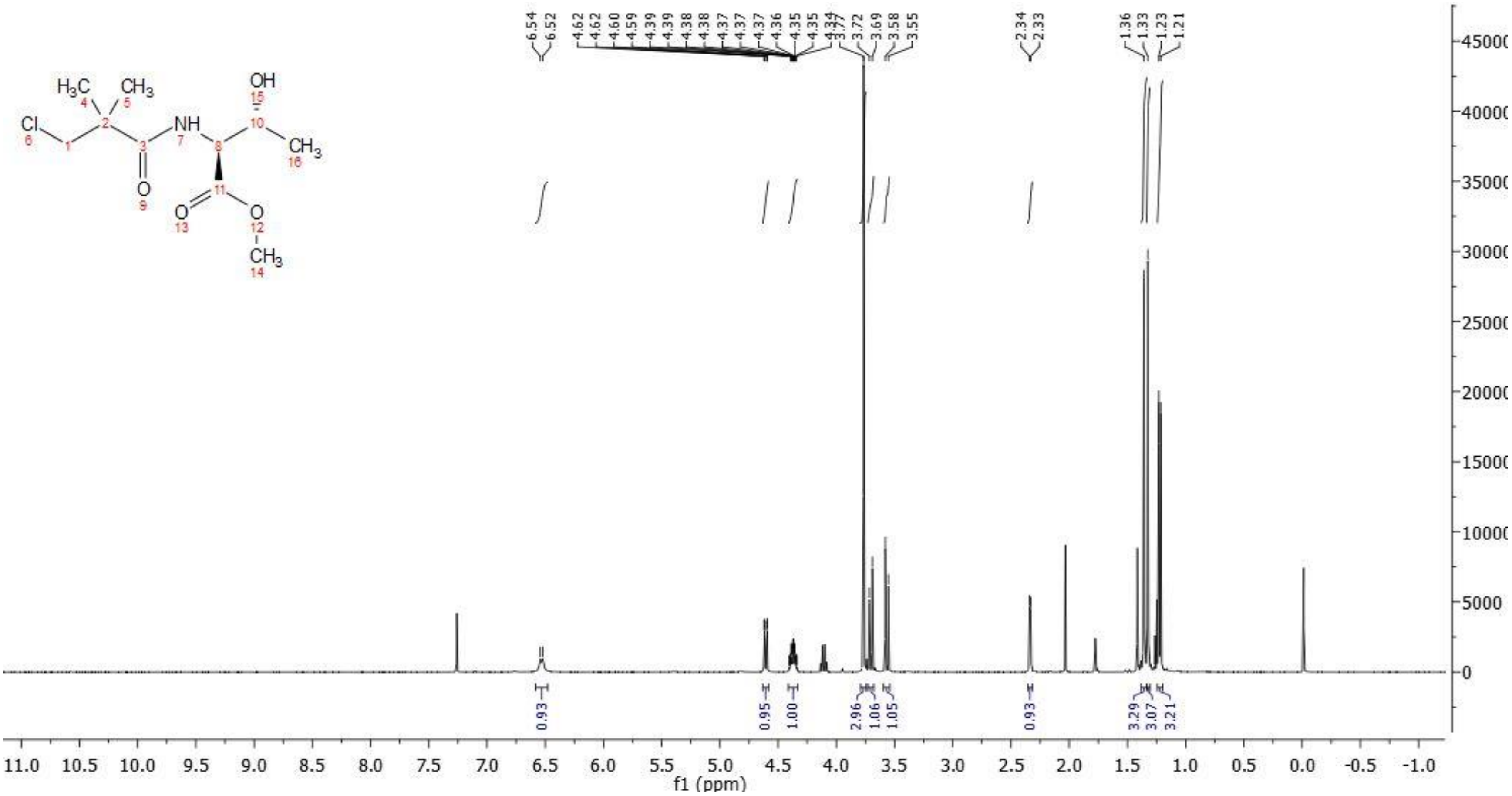

^{13}C NMR 11

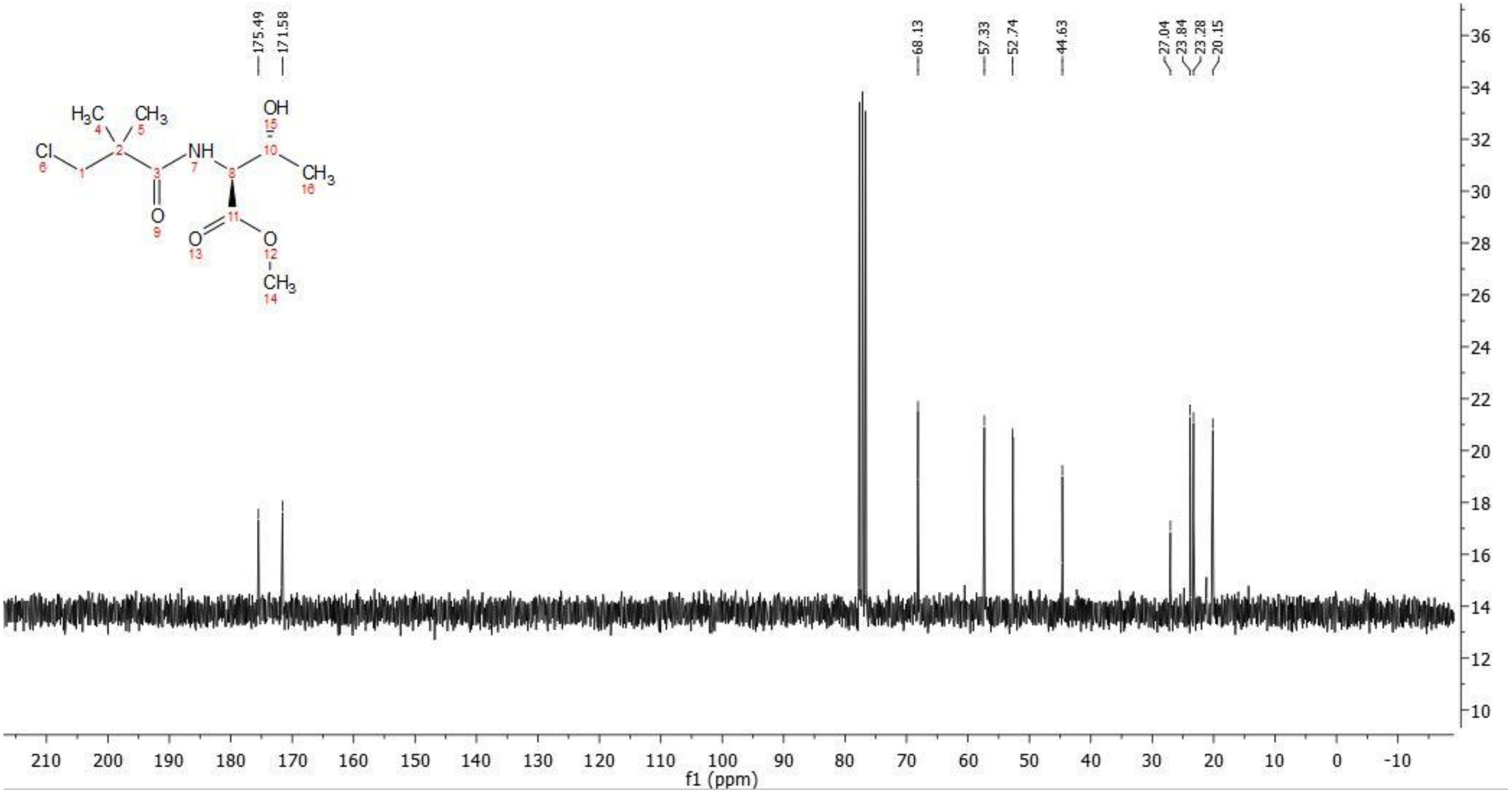

175.49
171.58
68.13
57.33
52.74
44.63
27.04
23.84
23.28
20.15
H3C
CH3
OH
Cl
NH
CH3
O
O
O
CH3
210
200
190
180
170
160
150
140
130
120
110
100
90
80
70
60
50
40
30
20
10
0
-10
f1 (ppm)
36
34
32
30
28
26
24
22
20
18
16
14
12
10

^{1}H NMR 12

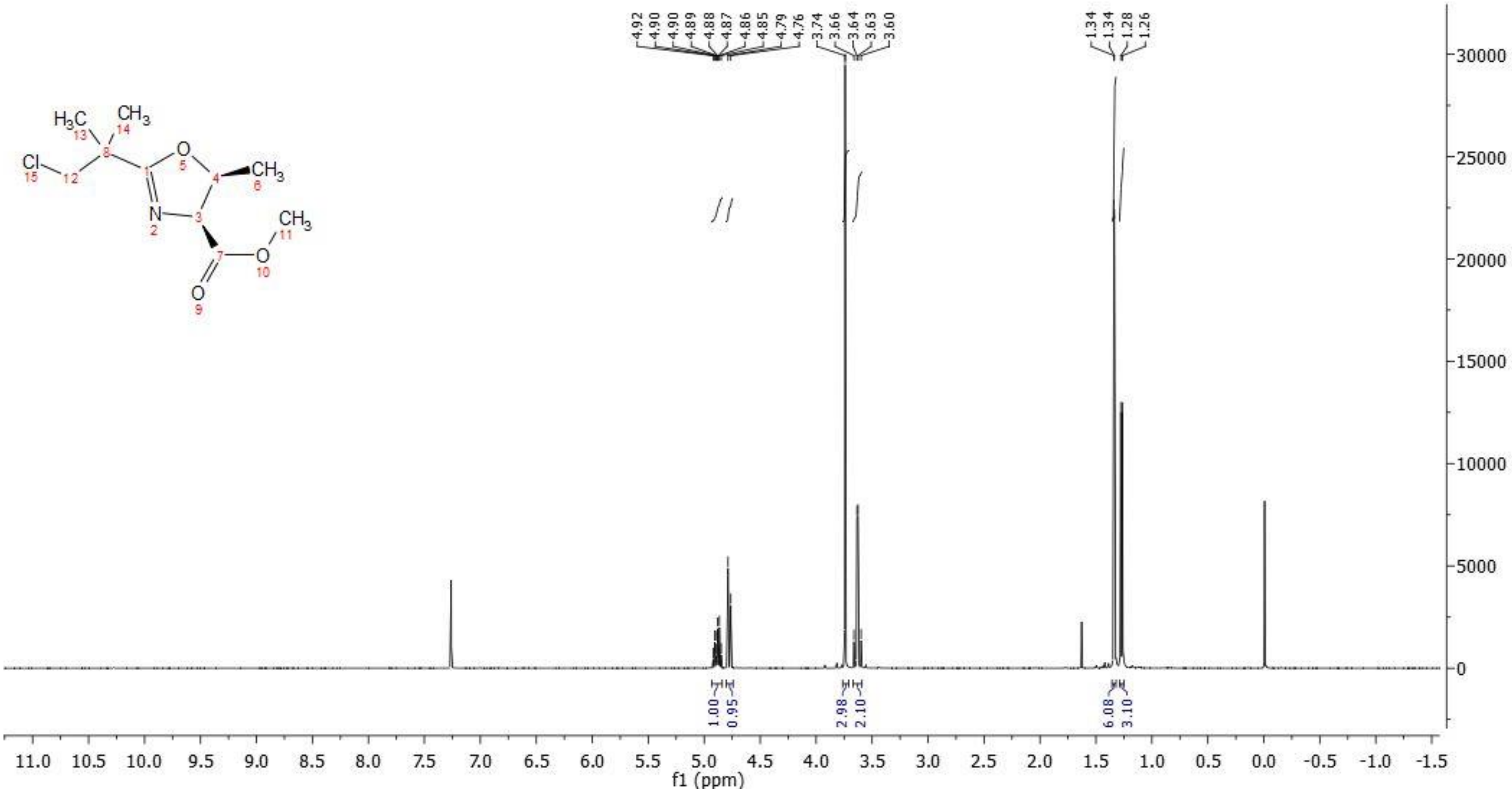

4.92
4.90
4.90
4.89
4.88
4.87
4.86
4.85
4.79
4.76
3.74
3.66
3.64
3.63
3.60
1.34
1.34
1.28
1.26
1.00
0.95
2.98
2.10
6.08
3.10
f1 (ppm)

^{13}C NMR 12

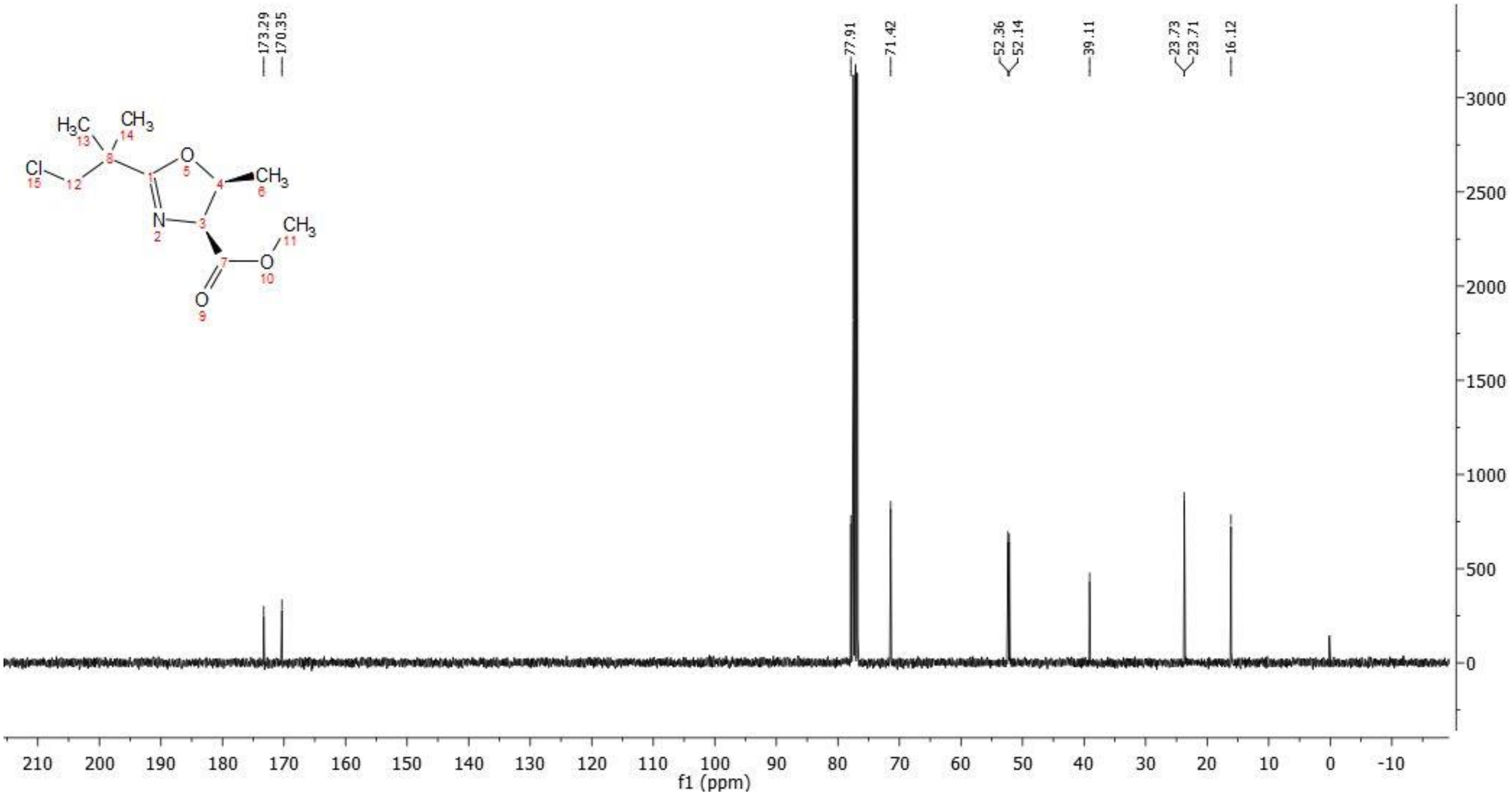

^{1}H NMR 13

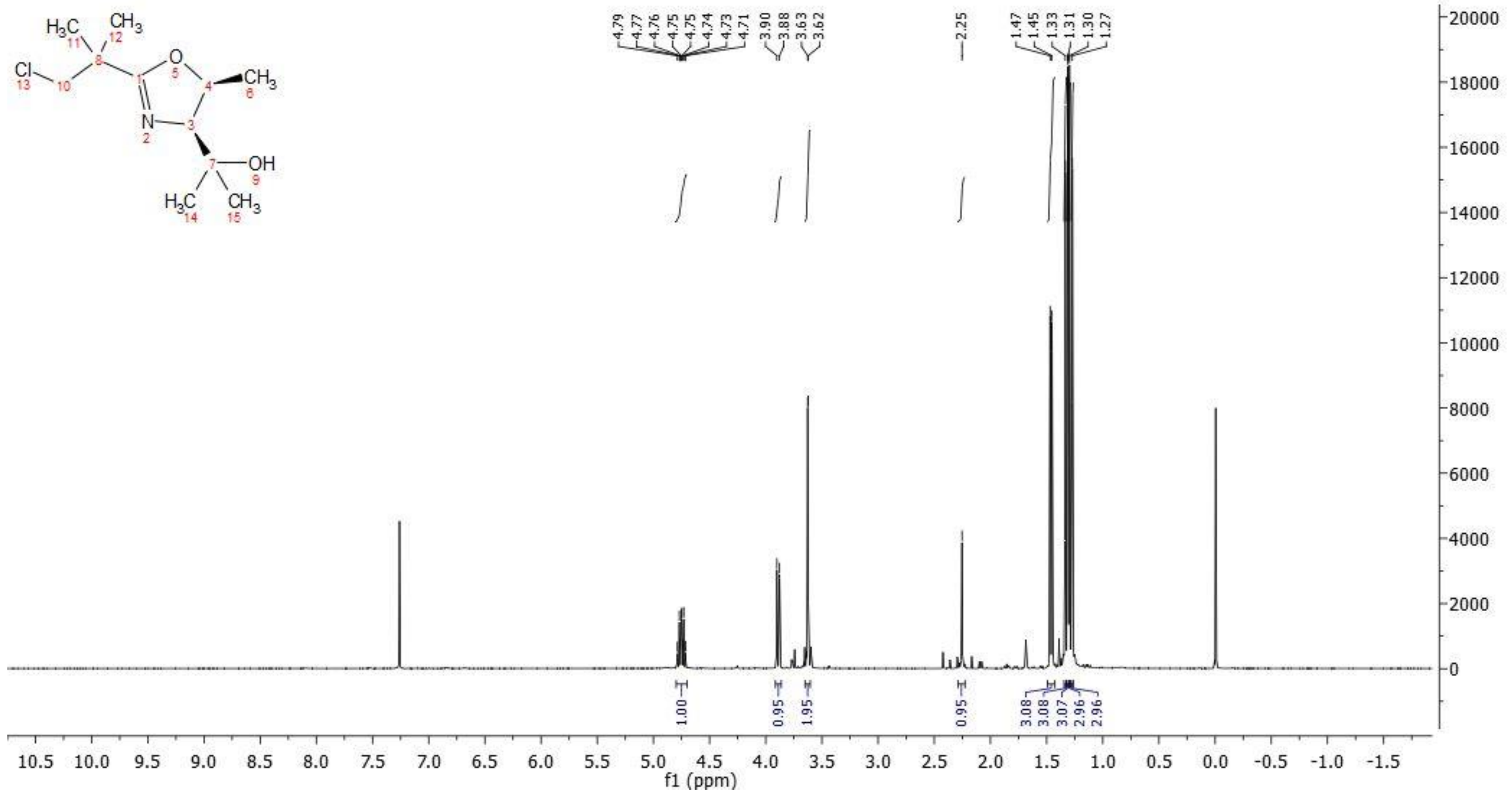

^{13}C NMR 13

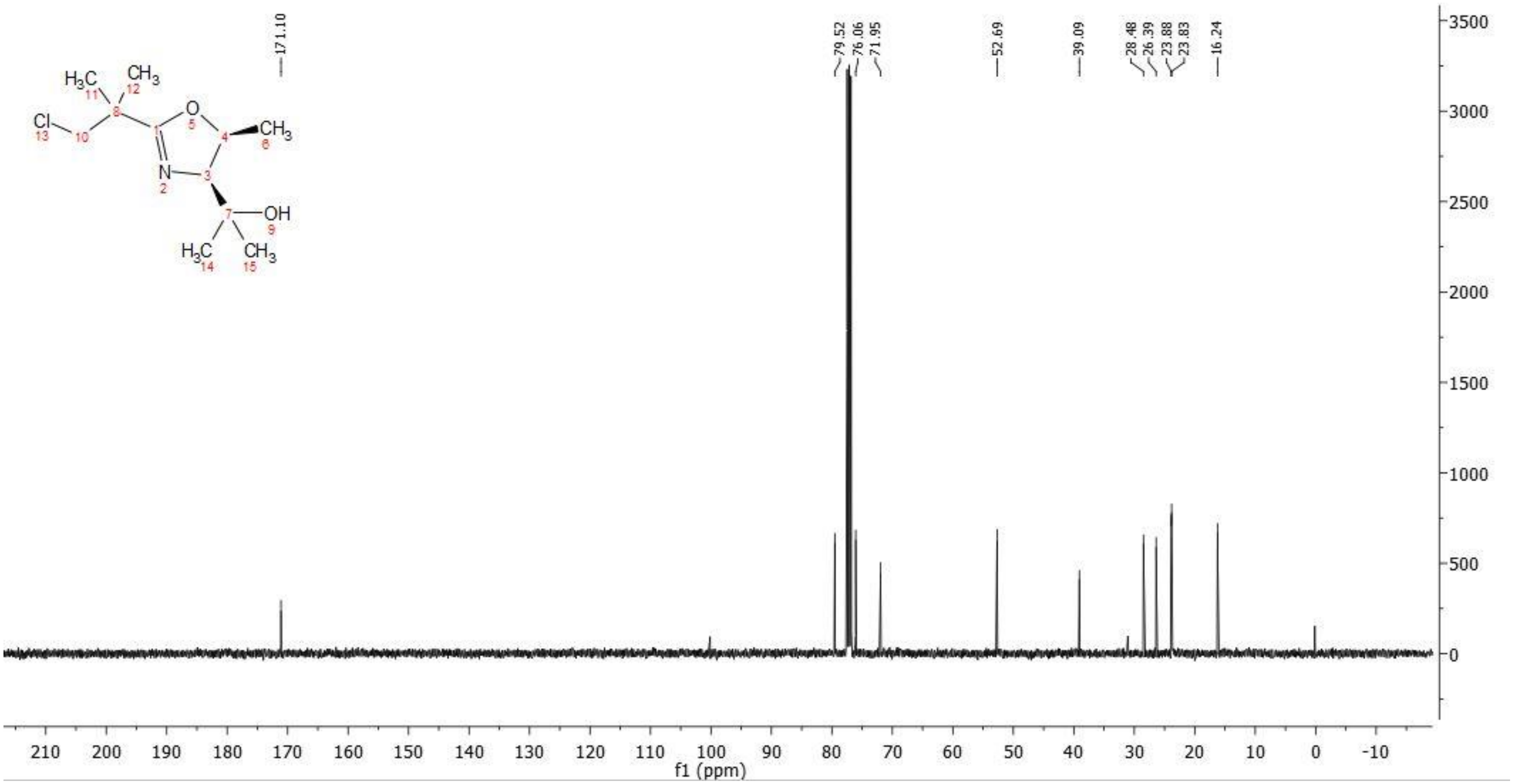
171.10
79.52
76.06
71.95
52.69
39.09
28.48
26.39
23.88
23.83
16.24
3500
3000
2500
2000
1500
1000
500
0
210 200 190 180 170 160 150 140 130 120 110 100 90 80 70 60 50 40 30 20 10 0 -10
f1 (ppm)
H3C
CH3
Cl
O
N
CH3
OH
H3C
CH3

^{1}H NMR 14

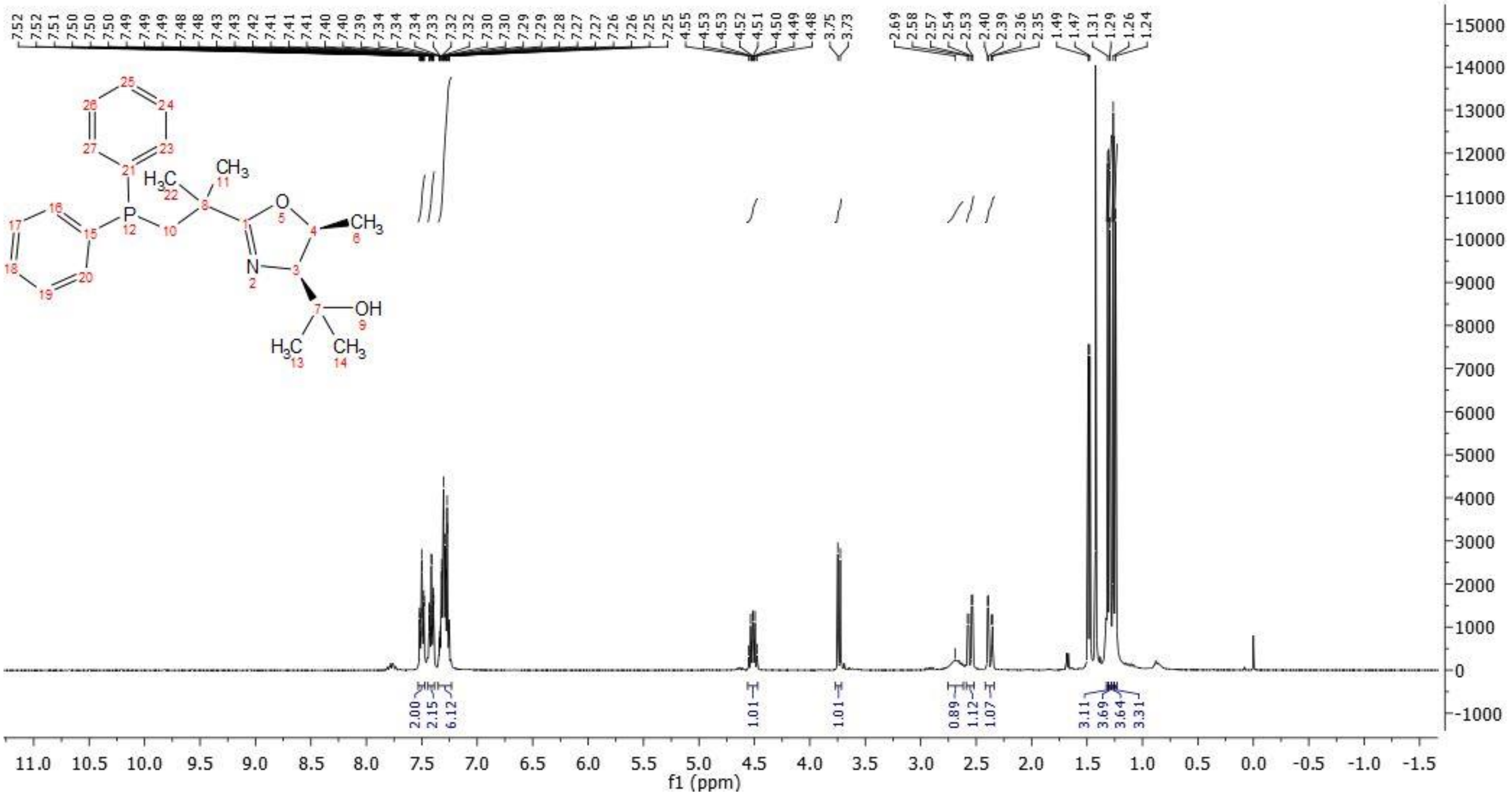

^{13}C NMR 14

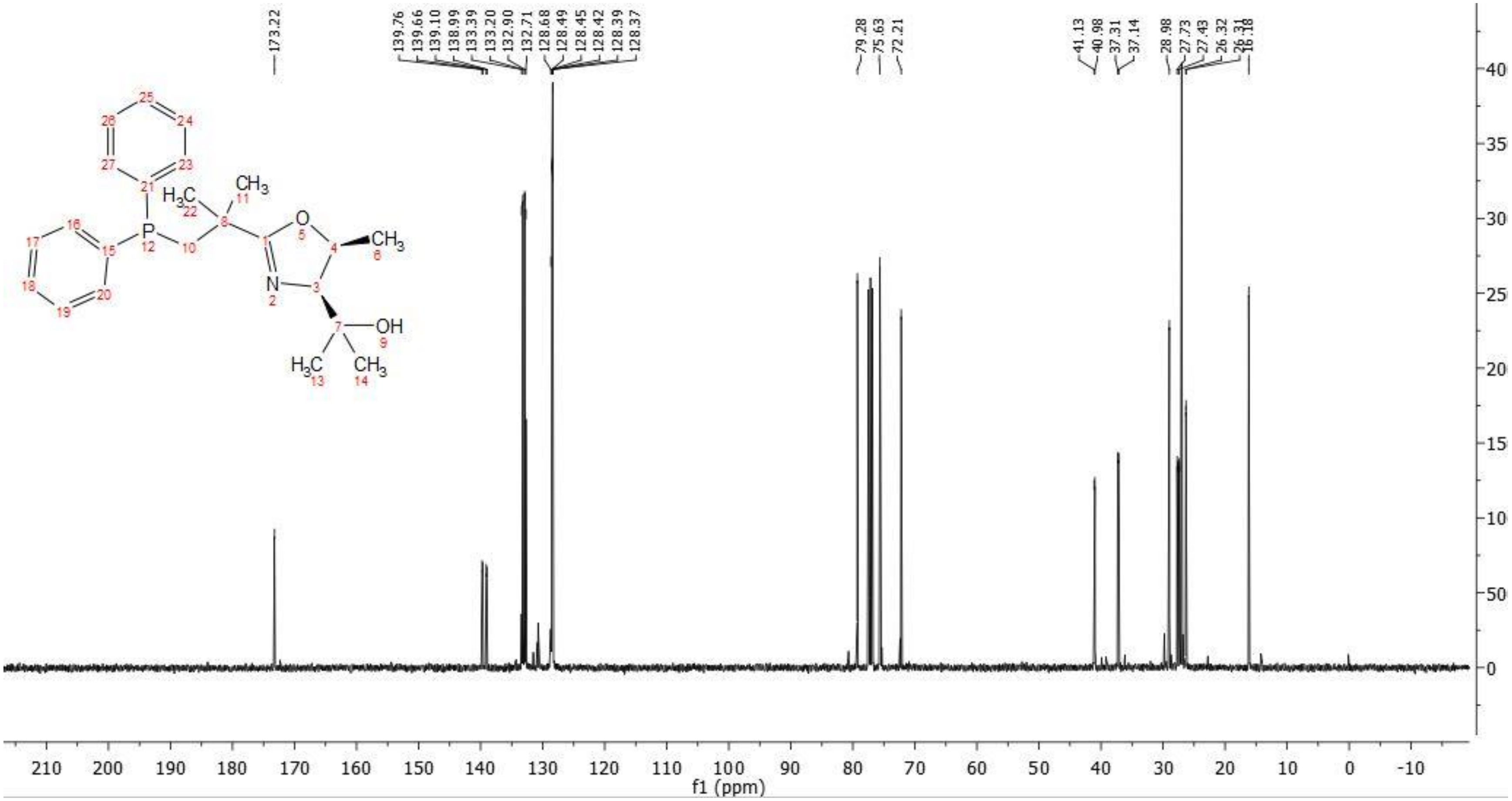
173.22
139.76
139.66
139.10
138.99
133.39
133.20
132.90
132.71
128.68
128.49
128.45
128.42
128.39
128.37
79.28
75.63
72.21
41.13
40.98
37.31
37.14
28.98
27.73
27.43
26.32
26.31
16.18
f1 (ppm)
OH
CH3
H3C
P
N
O

^{1}H NMR 14-TES

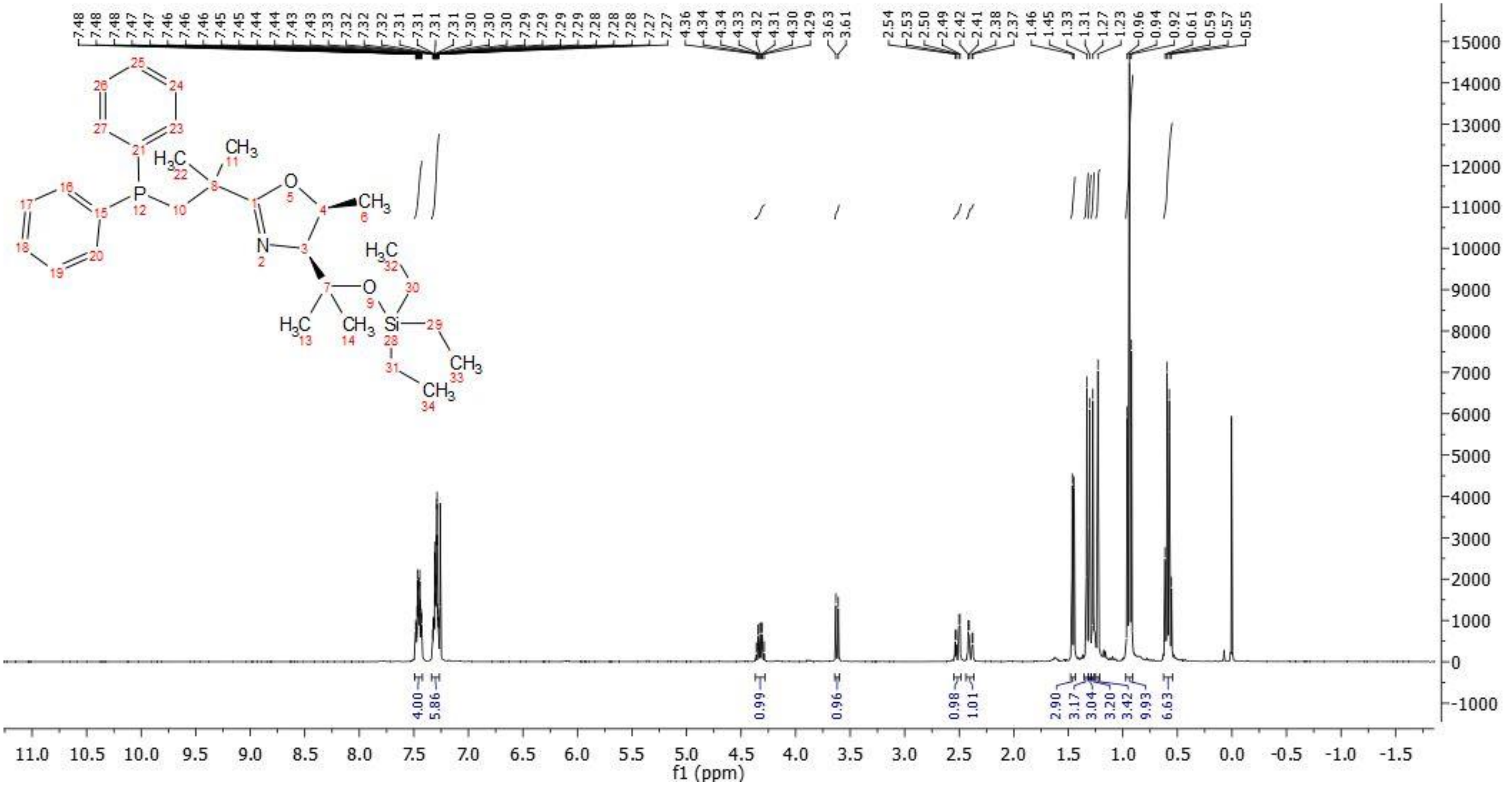

^{13}C NMR 14-TES

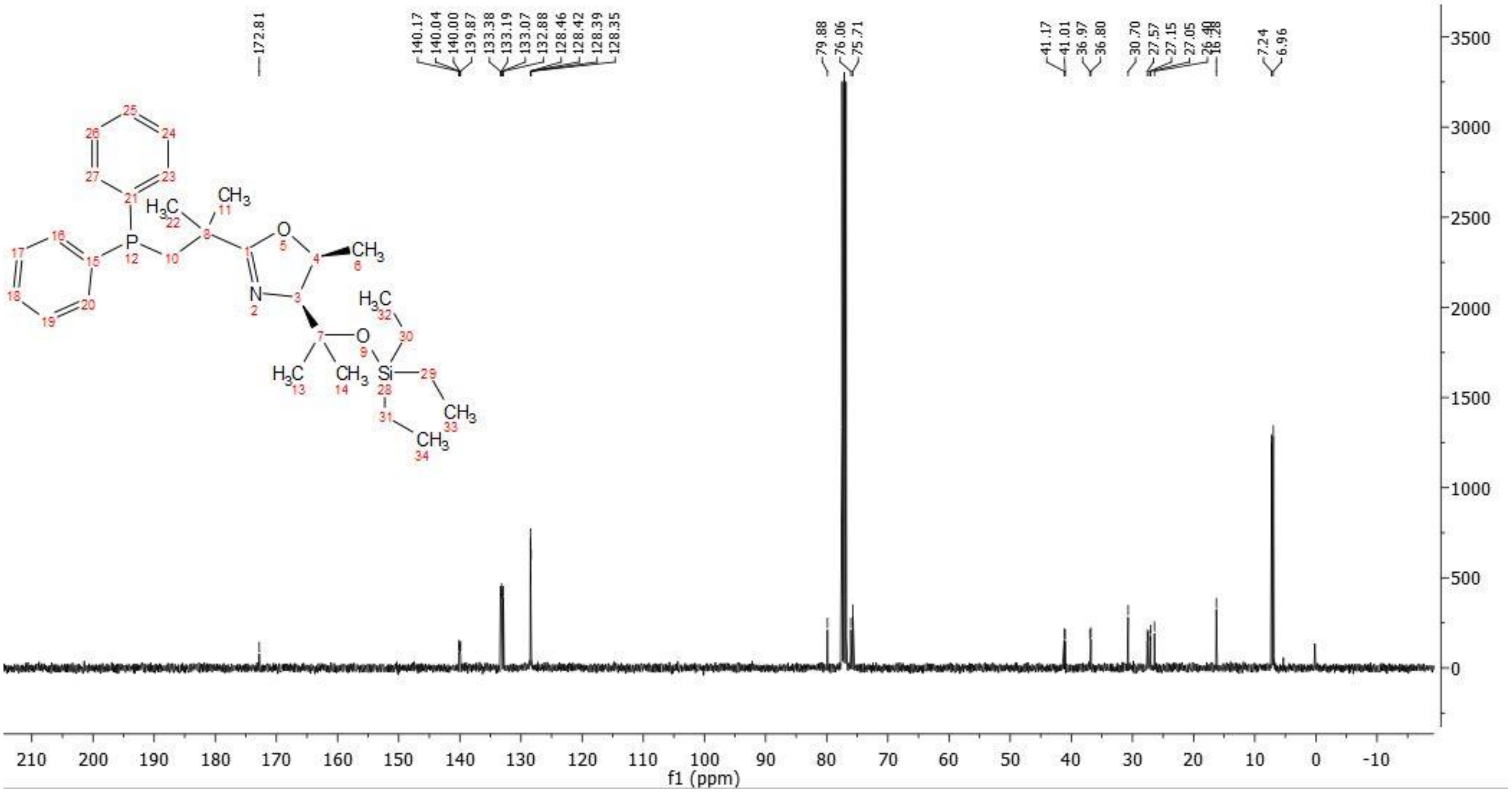

^{1}H NMR Ir-14-TES

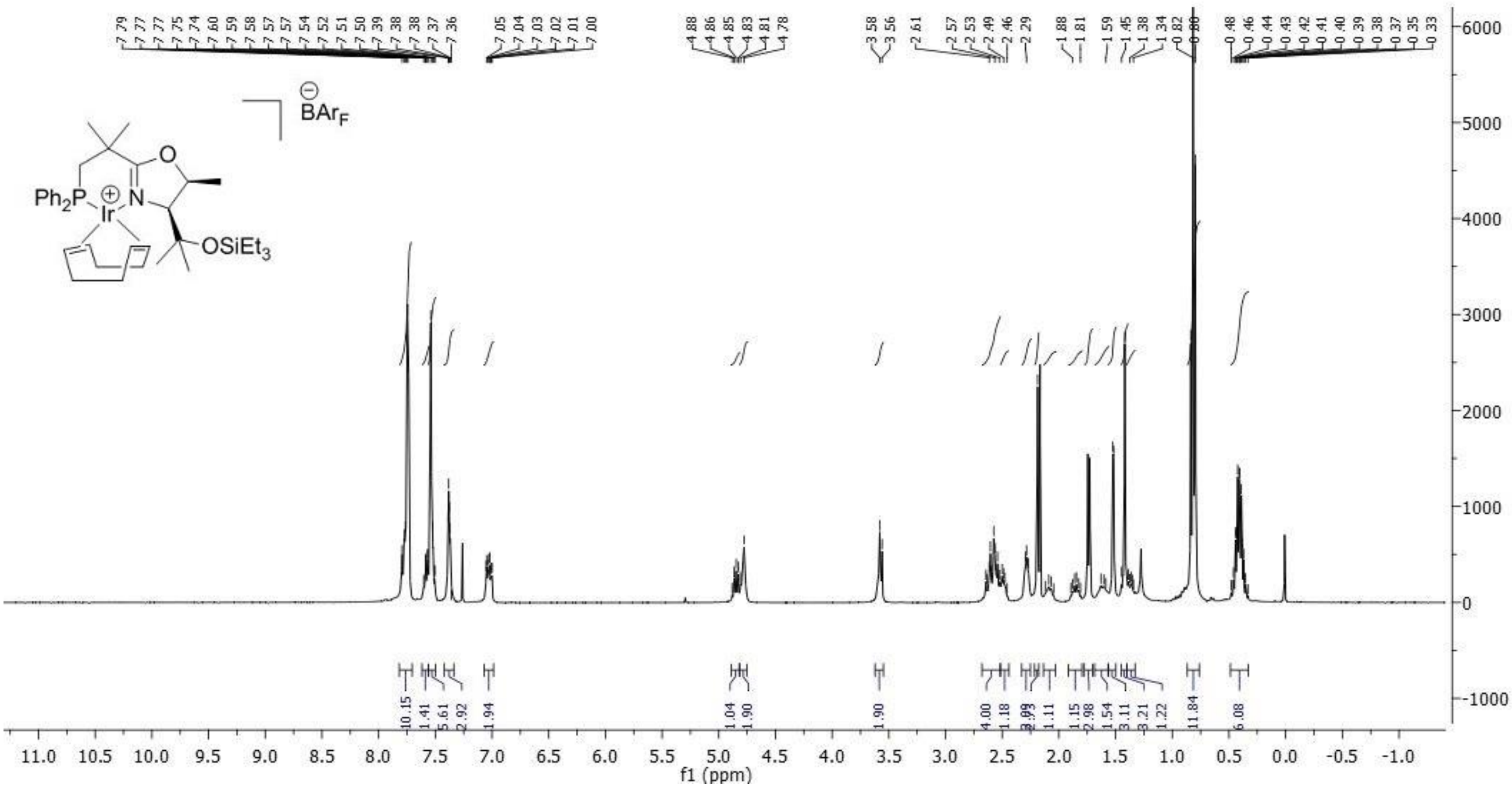

^{13}C NMR Ir-14-TES

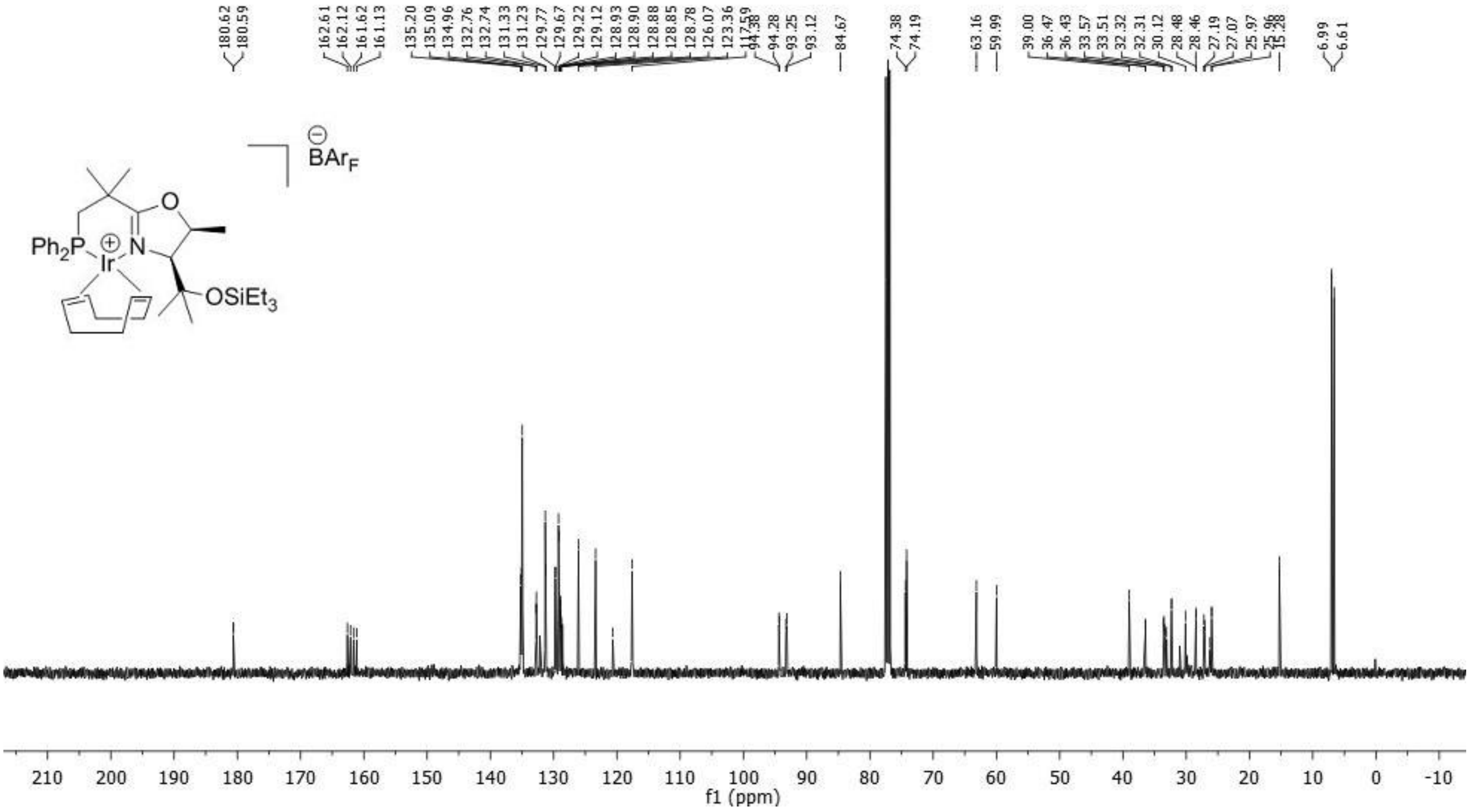

^{1}**H NMR 14-TBDMS**

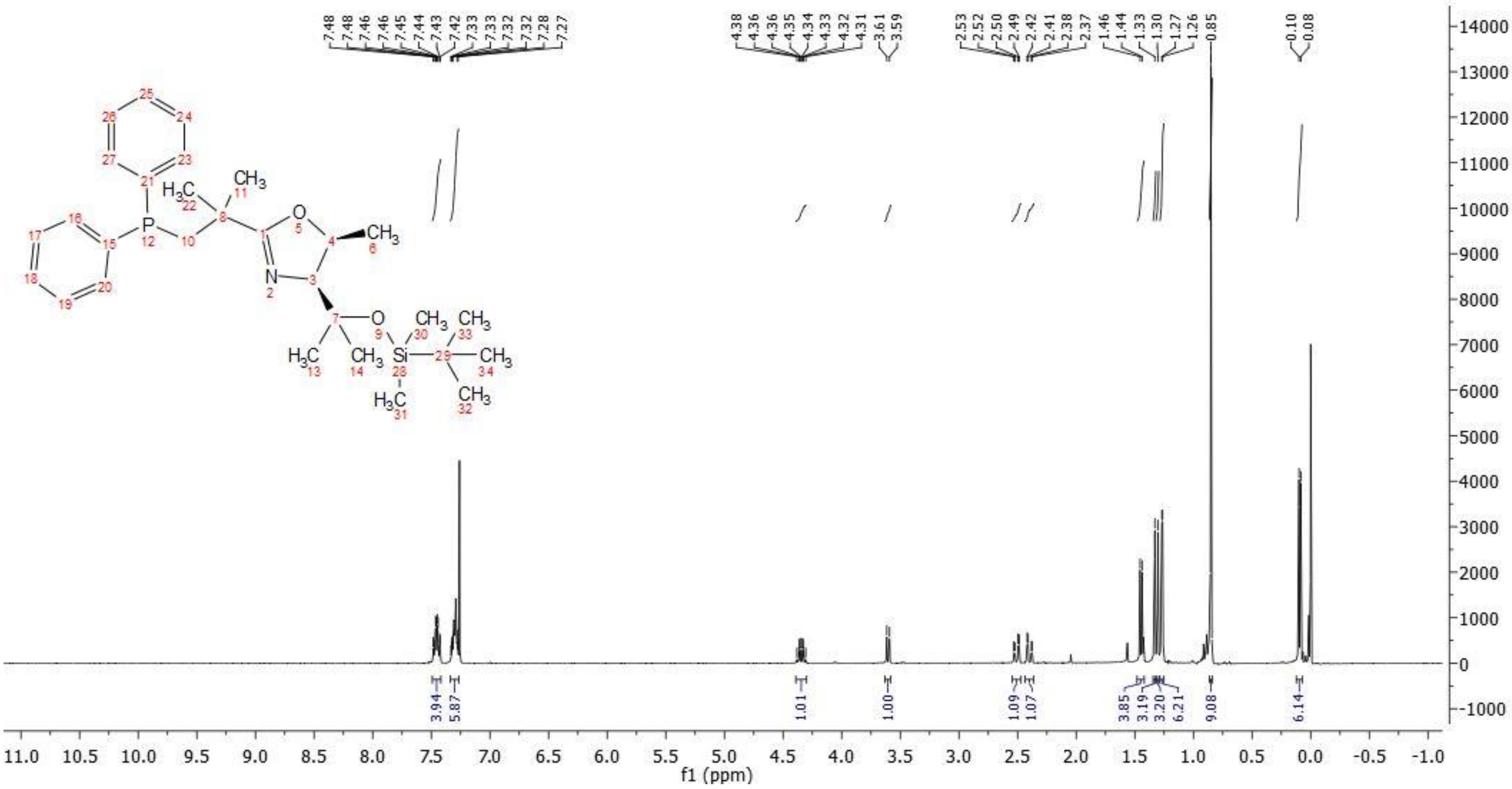

^{13}C NMR 14-TBDMS

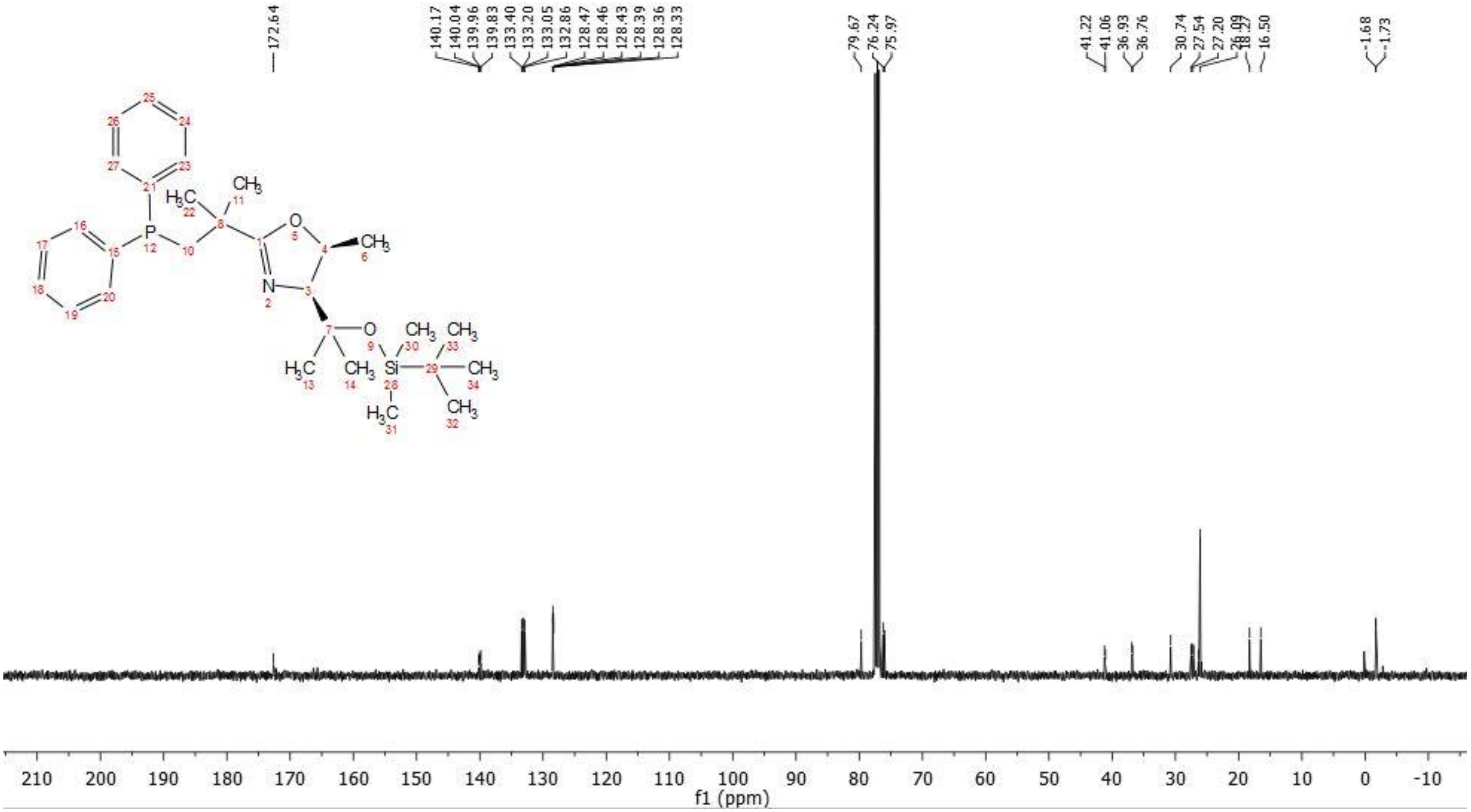

^{1}H NMR Ir-14-TBDMS

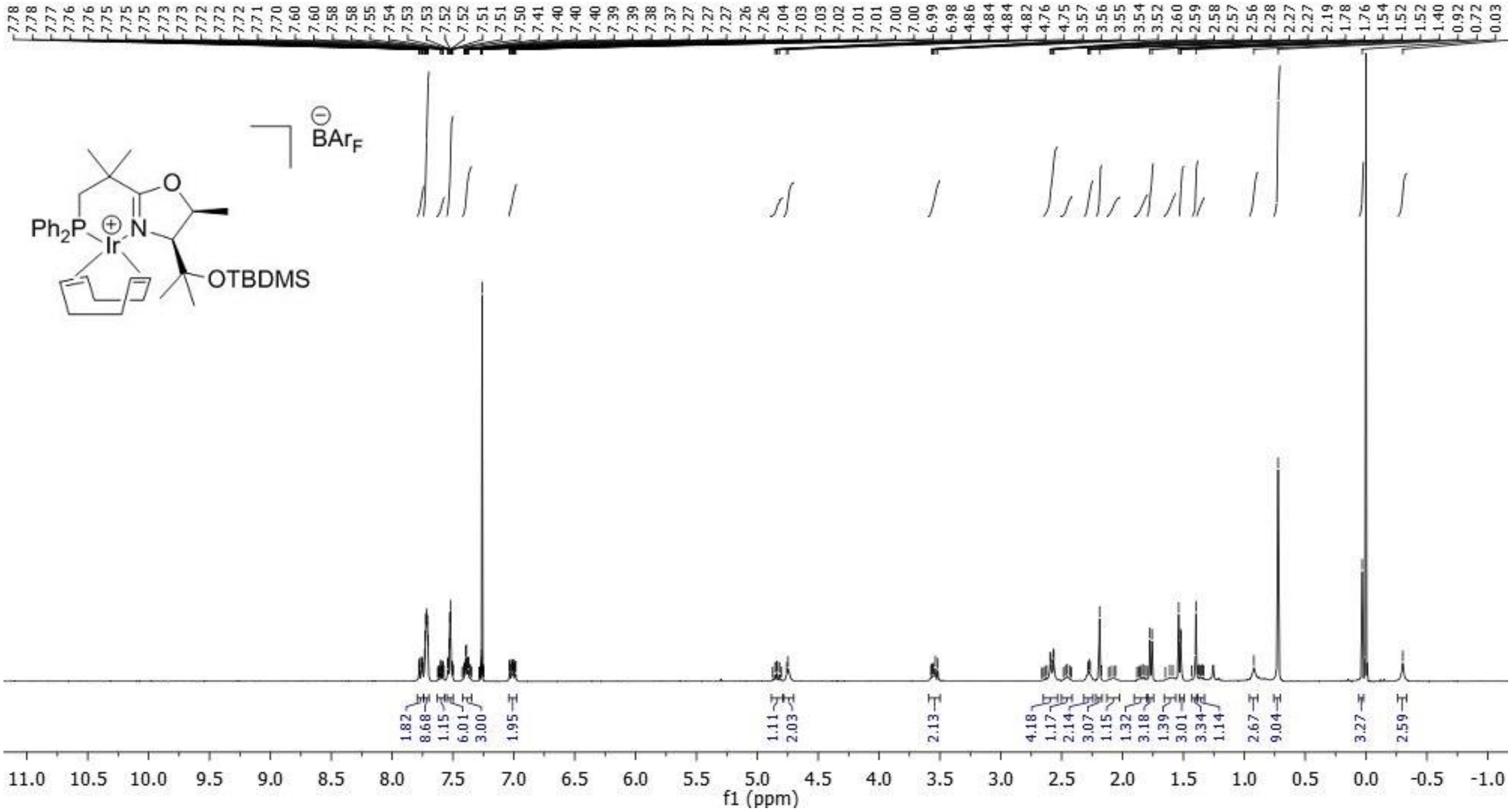

^{1}H NMR 17

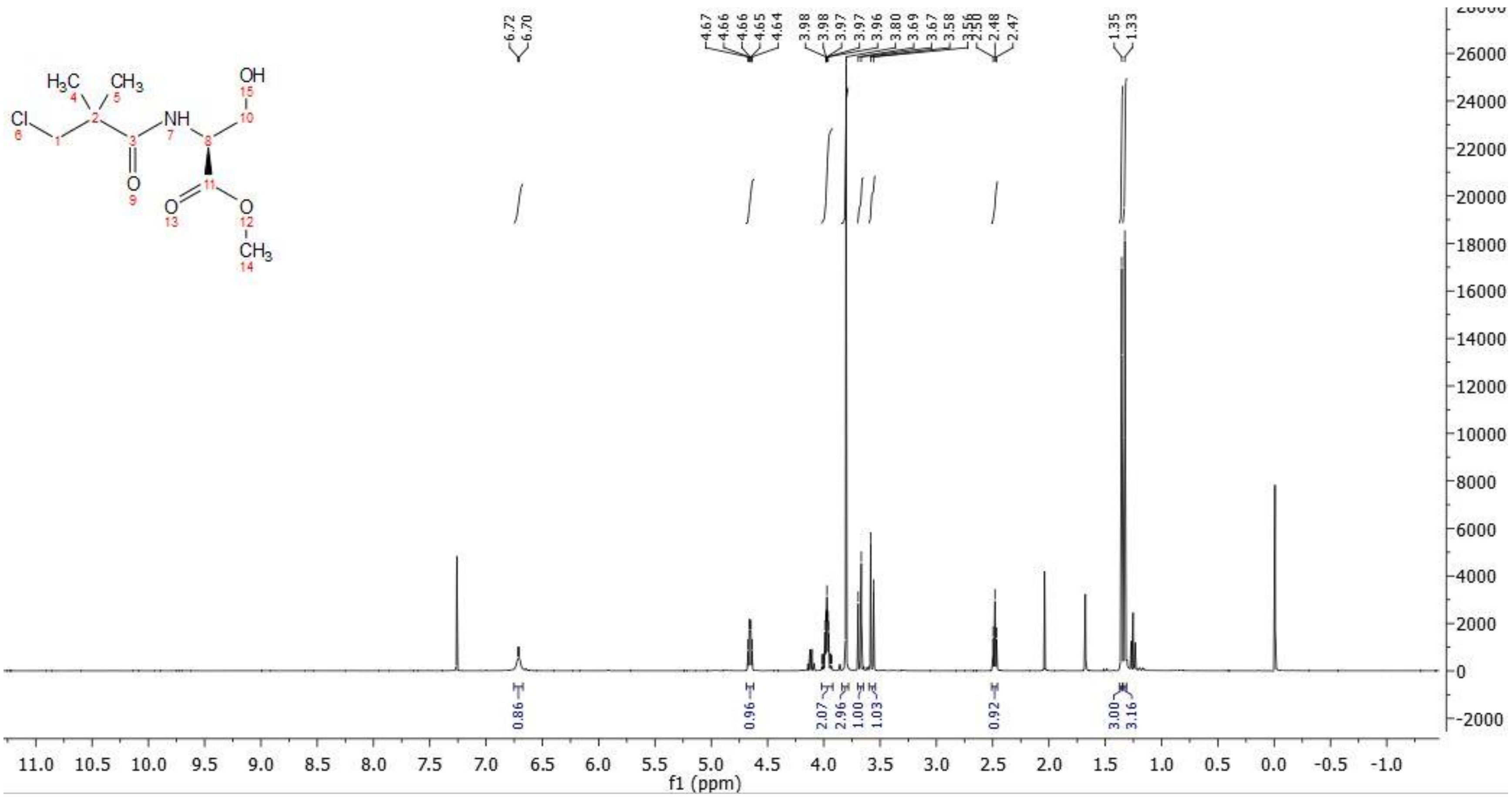
6.72
6.70
4.67
4.66
4.66
4.65
4.64
3.98
3.98
3.97
3.97
3.96
3.80
3.69
3.67
3.58
3.56
2.50
2.48
2.47
1.35
1.33
0.86
0.96
2.07
2.96
1.00
1.03
0.92
3.00
3.16
f1 (ppm)

^{13}C **NMR 17**

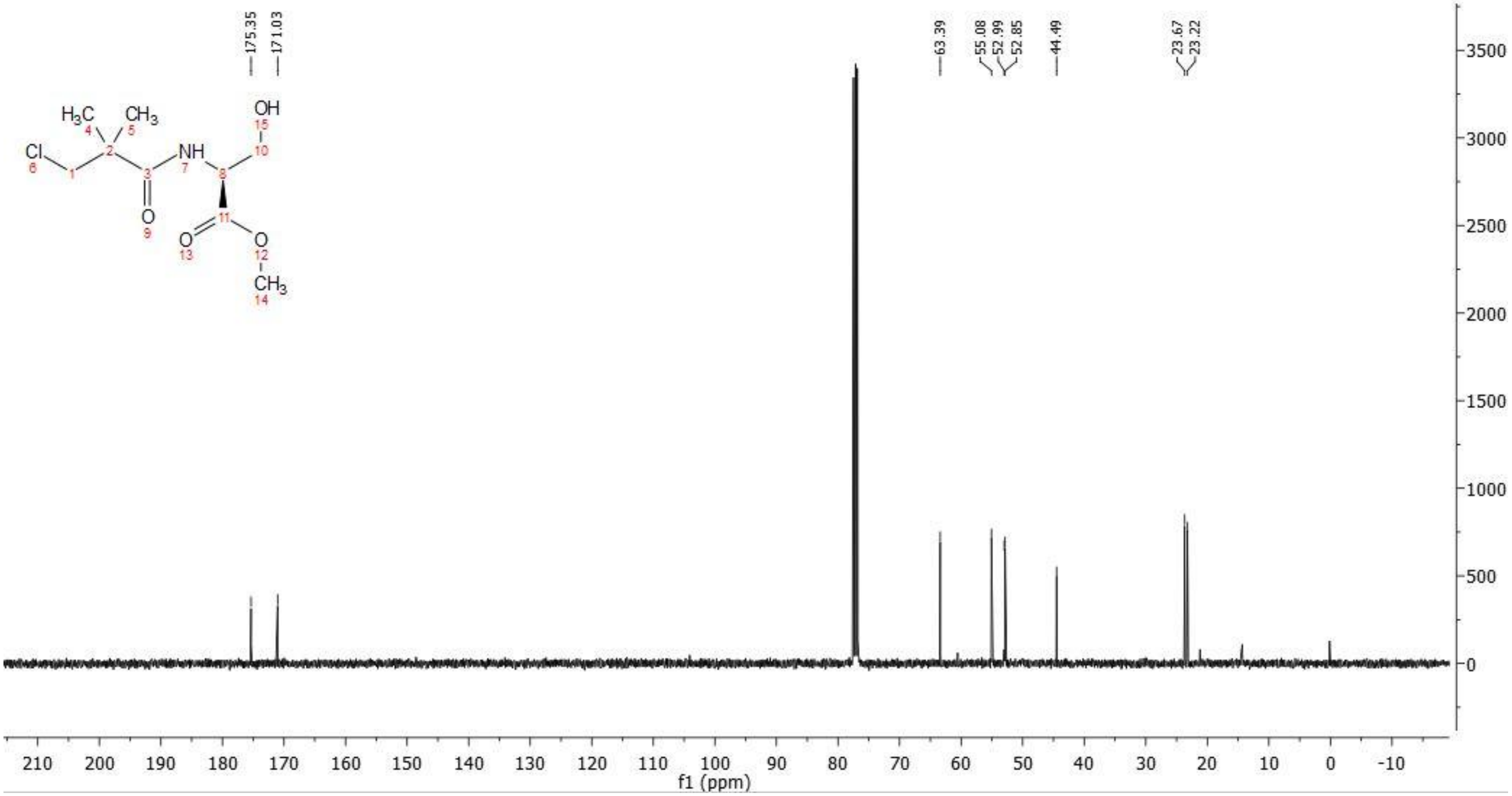

175.35
171.03
63.39
55.08
52.99
52.85
44.49
23.67
23.22
H3C
CH3
Cl
NH
OH
O
O
O
CH3
3500
3000
2500
2000
1500
1000
500
0
210
200
190
180
170
160
150
140
130
120
110
100
90
80
70
60
50
40
30
20
10
0
-10
f1 (ppm)

^{1}H NMR 18

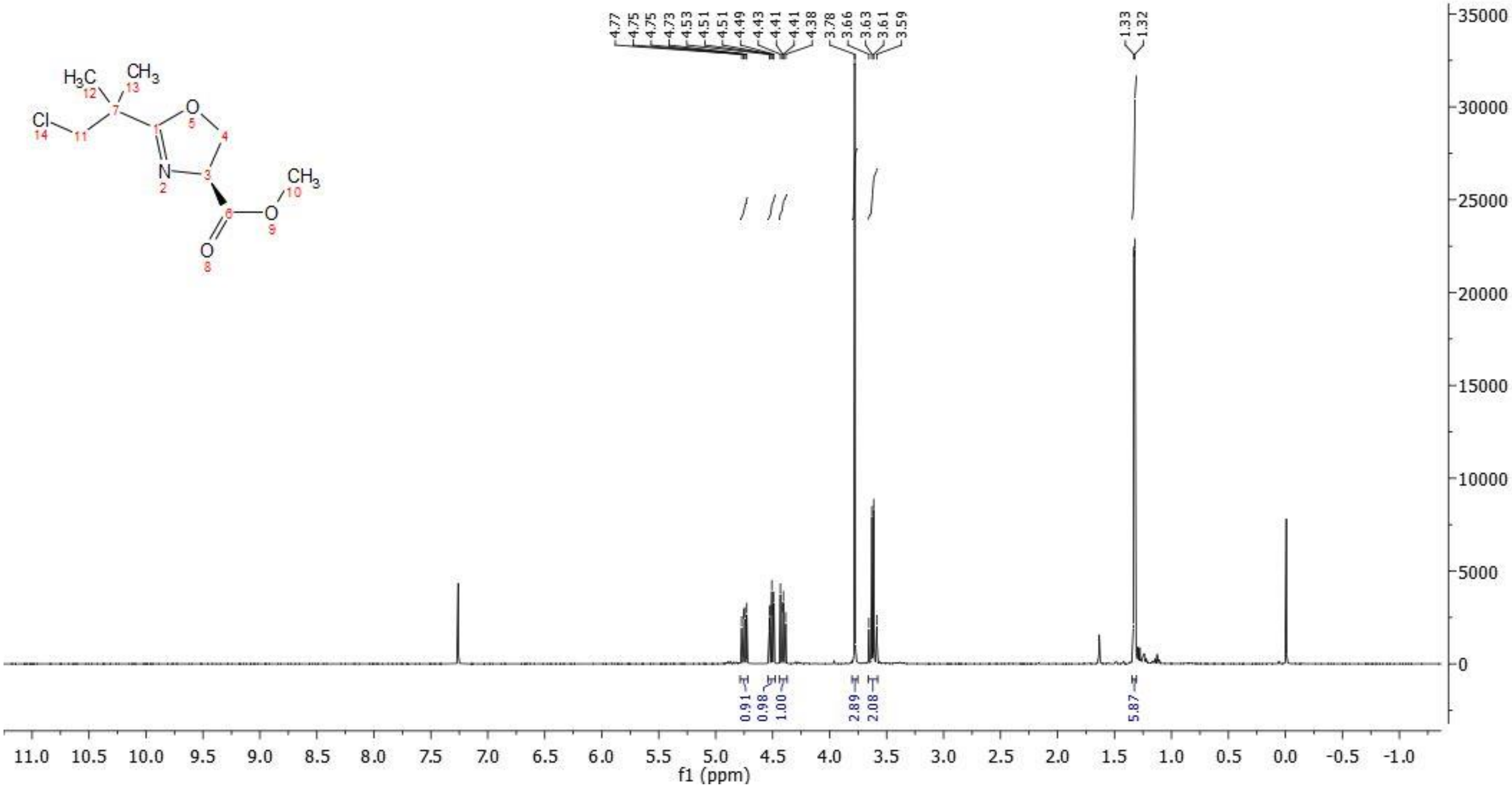

4.77
4.75
4.75
4.73
4.53
4.51
4.51
4.49
4.43
4.41
4.41
4.38
3.78
3.66
3.63
3.61
3.59
1.33
1.32
0.91
0.98
1.00
2.89
2.08
5.87
f1 (ppm)

^{13}C NMR 18

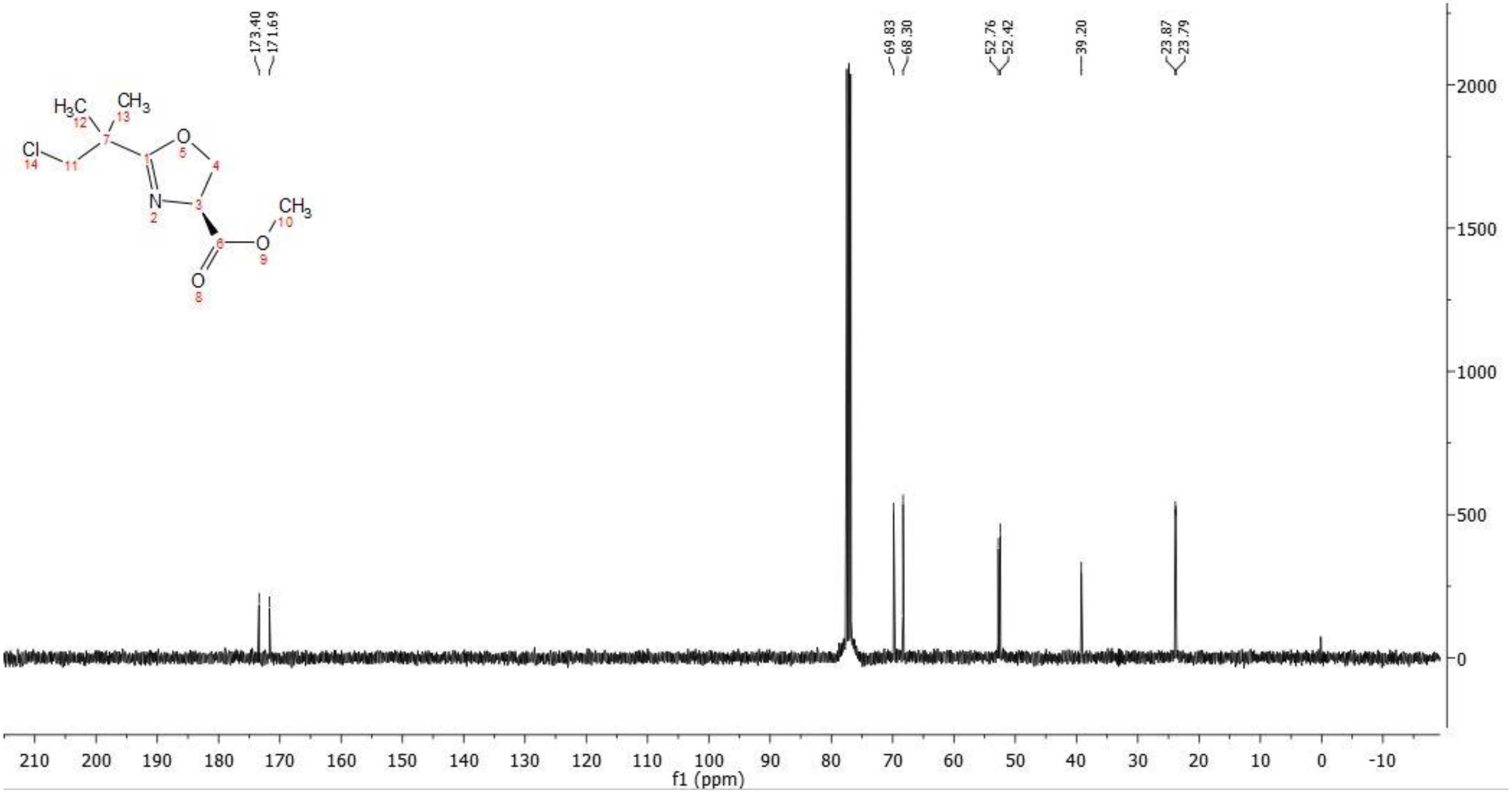
173.40
171.69
69.83
68.30
52.76
52.42
39.20
23.87
23.79
2000
1500
1000
500
0
210 200 190 180 170 160 150 140 130 120 110 100 90 80 70 60 50 40 30 20 10 0 -10
f1 (ppm)

^{1}H NMR 19

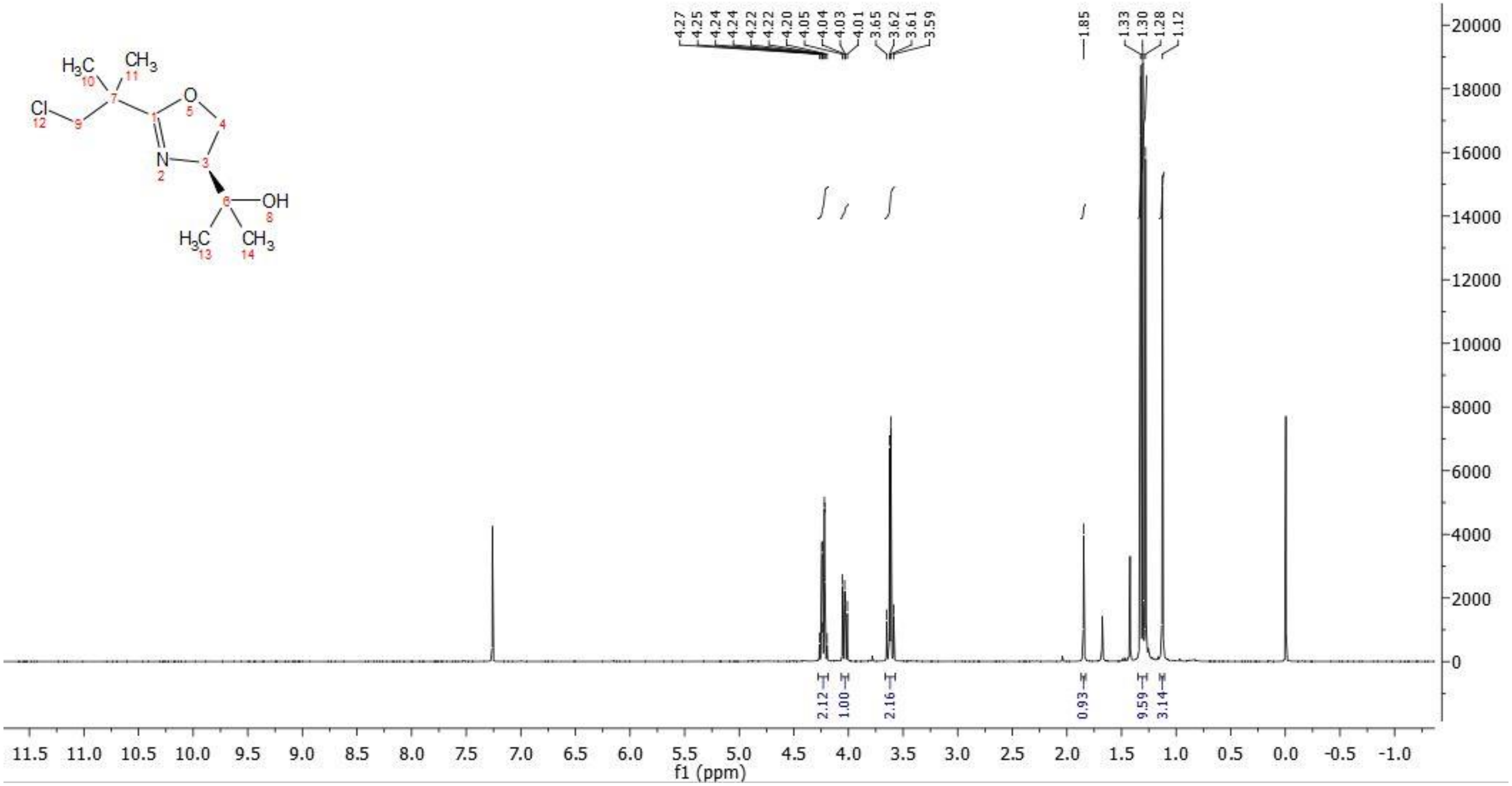

4.27
4.25
4.24
4.24
4.22
4.22
4.20
4.05
4.04
4.03
4.01
3.65
3.62
3.61
3.59
1.85
1.33
1.30
1.28
1.12
2.12
1.00
2.16
0.93
9.59
3.14
f1 (ppm)

^{13}C NMR 19

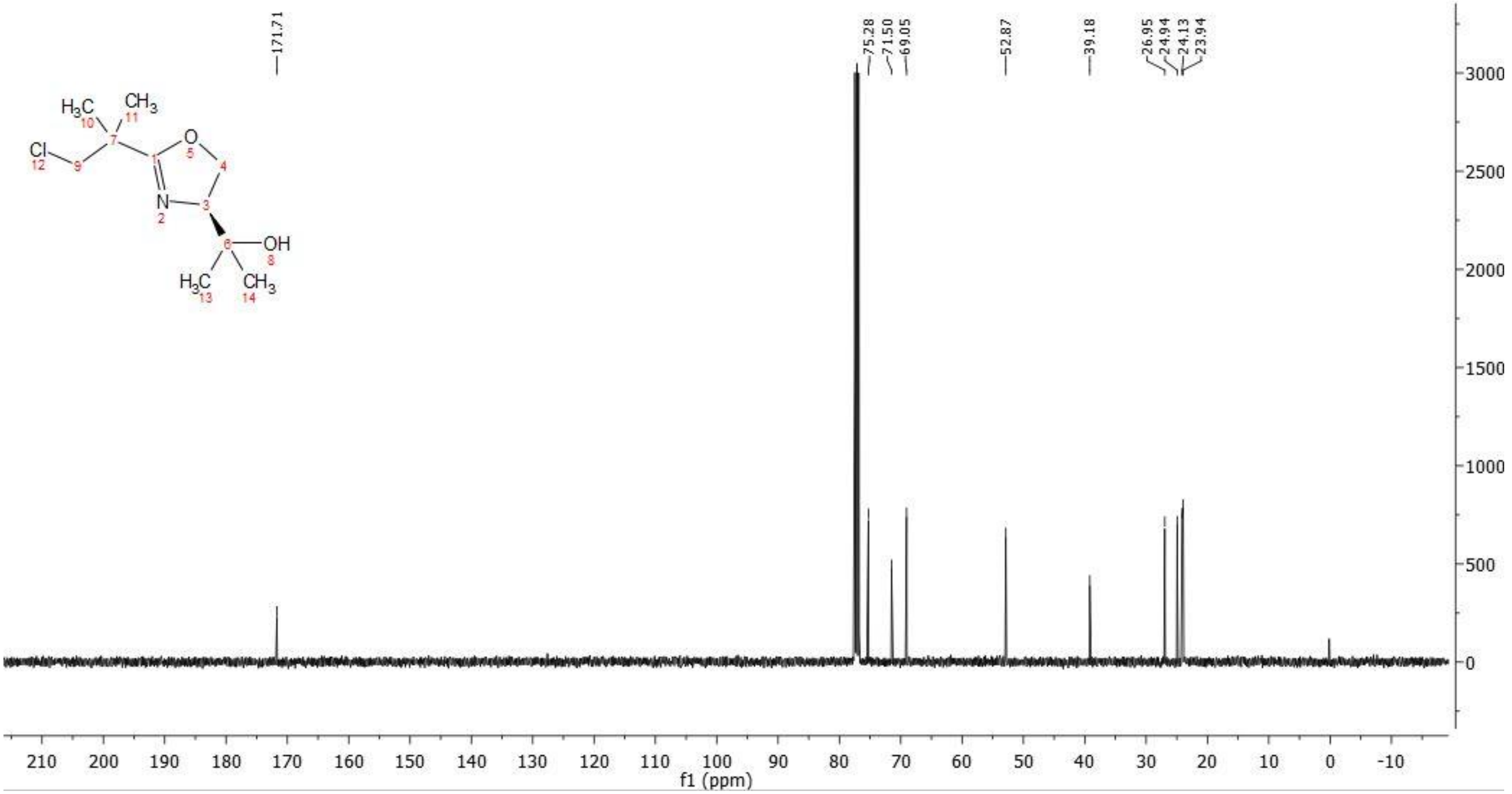
171.71
75.28
71.50
69.05
52.87
39.18
26.95
24.94
24.13
23.94
H3C
CH3
Cl
O
N
OH
H3C
CH3
3000
2500
2000
1500
1000
500
0
210
200
190
180
170
160
150
140
130
120
110
100
90
80
70
60
50
40
30
20
10
0
-10
f1 (ppm)

^{1}H NMR 20

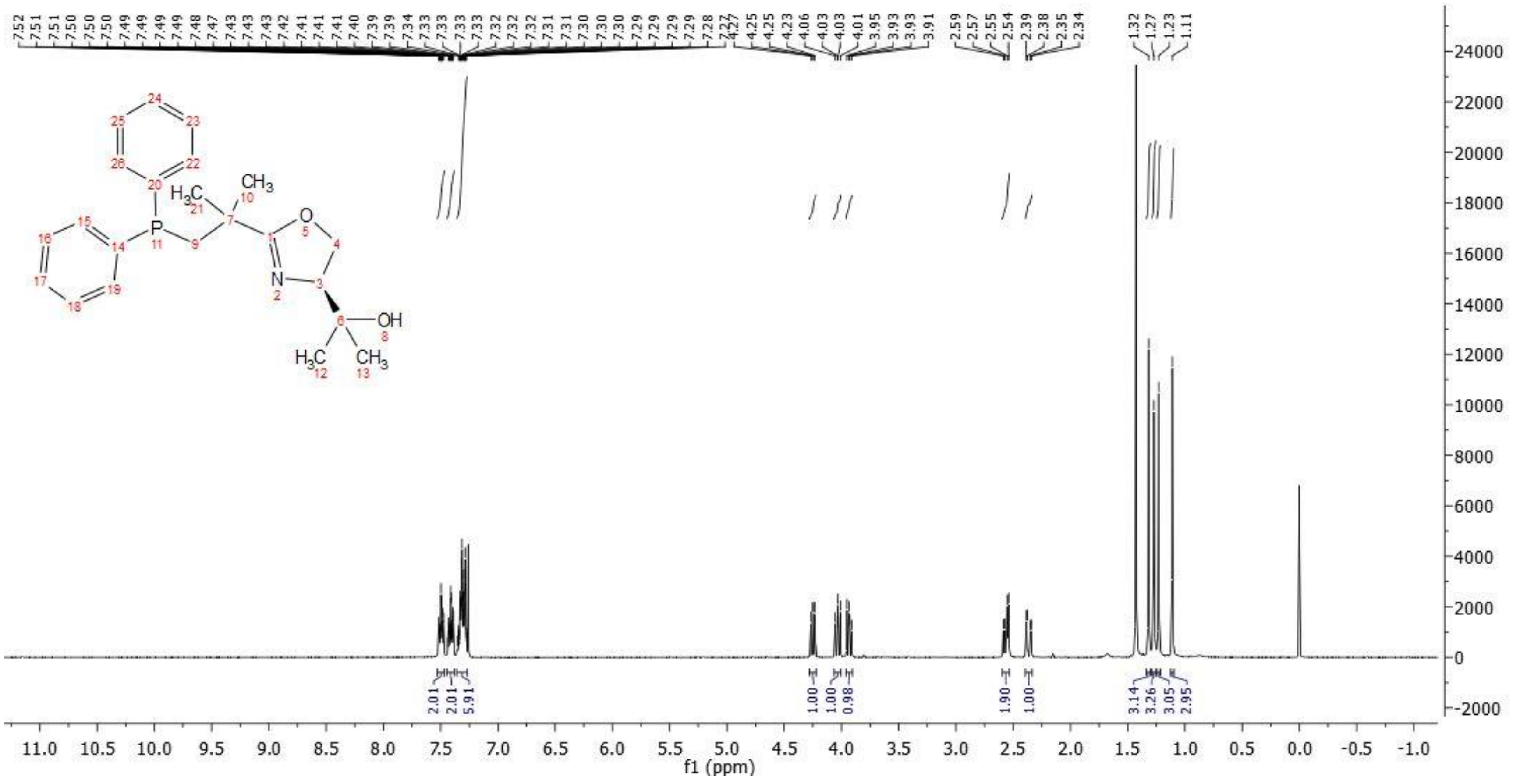

^{13}C NMR 20

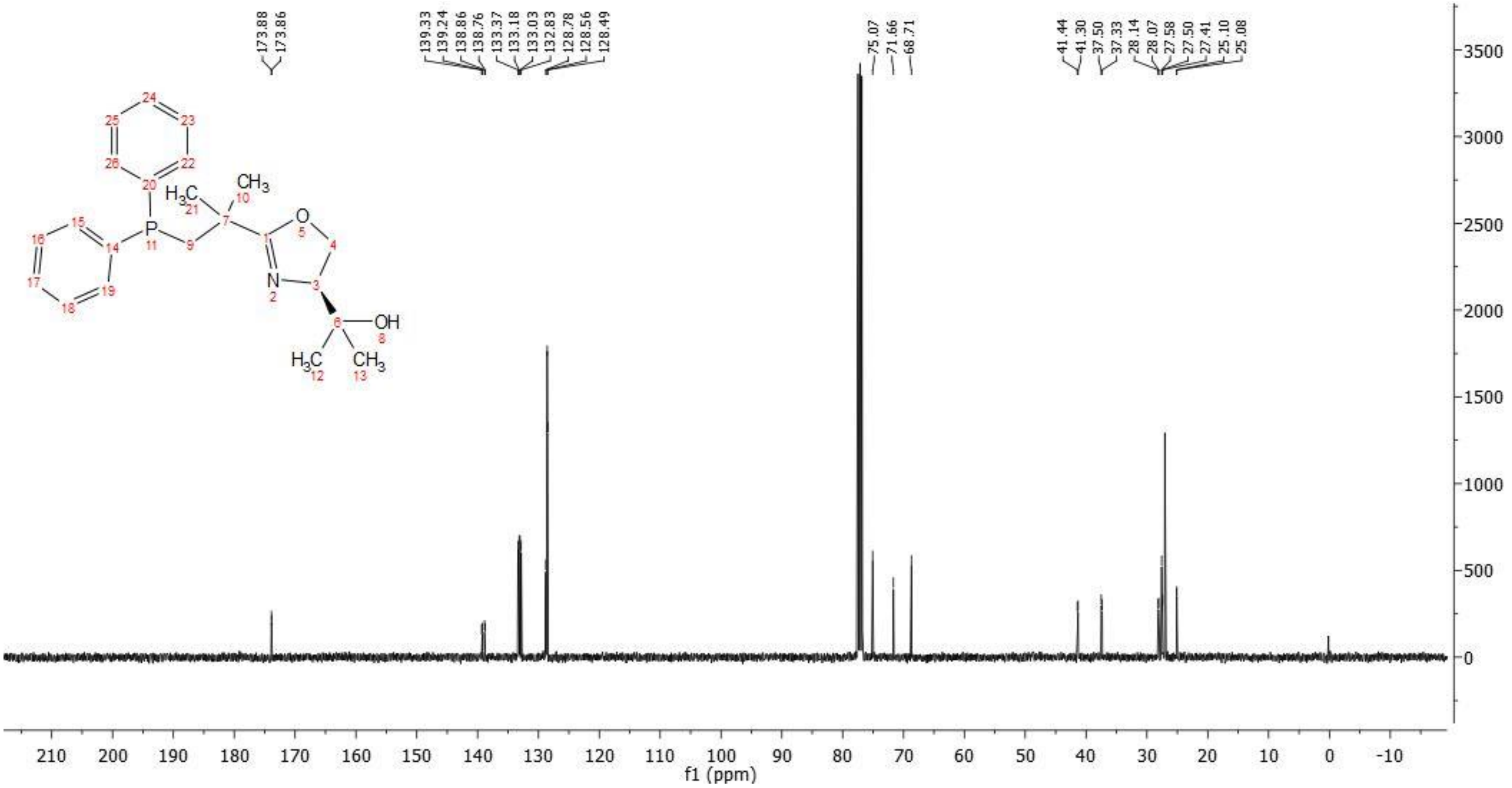

173.88
173.86
139.33
139.24
138.86
138.76
133.37
133.18
133.03
132.83
128.78
128.56
128.49
75.07
71.66
68.71
41.44
41.30
37.50
37.33
28.14
28.07
27.58
27.50
27.41
25.10
25.08
f1 (ppm)
